# Supplementary figures and images for: Optimal waist circumference cut-off points for predicting metabolic syndrome among females of reproductive age in Wakiso district, central Uganda
Source: PLOS Glob Public Health. 2025 Apr 9;5(4):e0003059. doi: 10.1371/journal.pgph.0003059 (PMC11981207; doi:10.1371/journal.pgph.0003059)

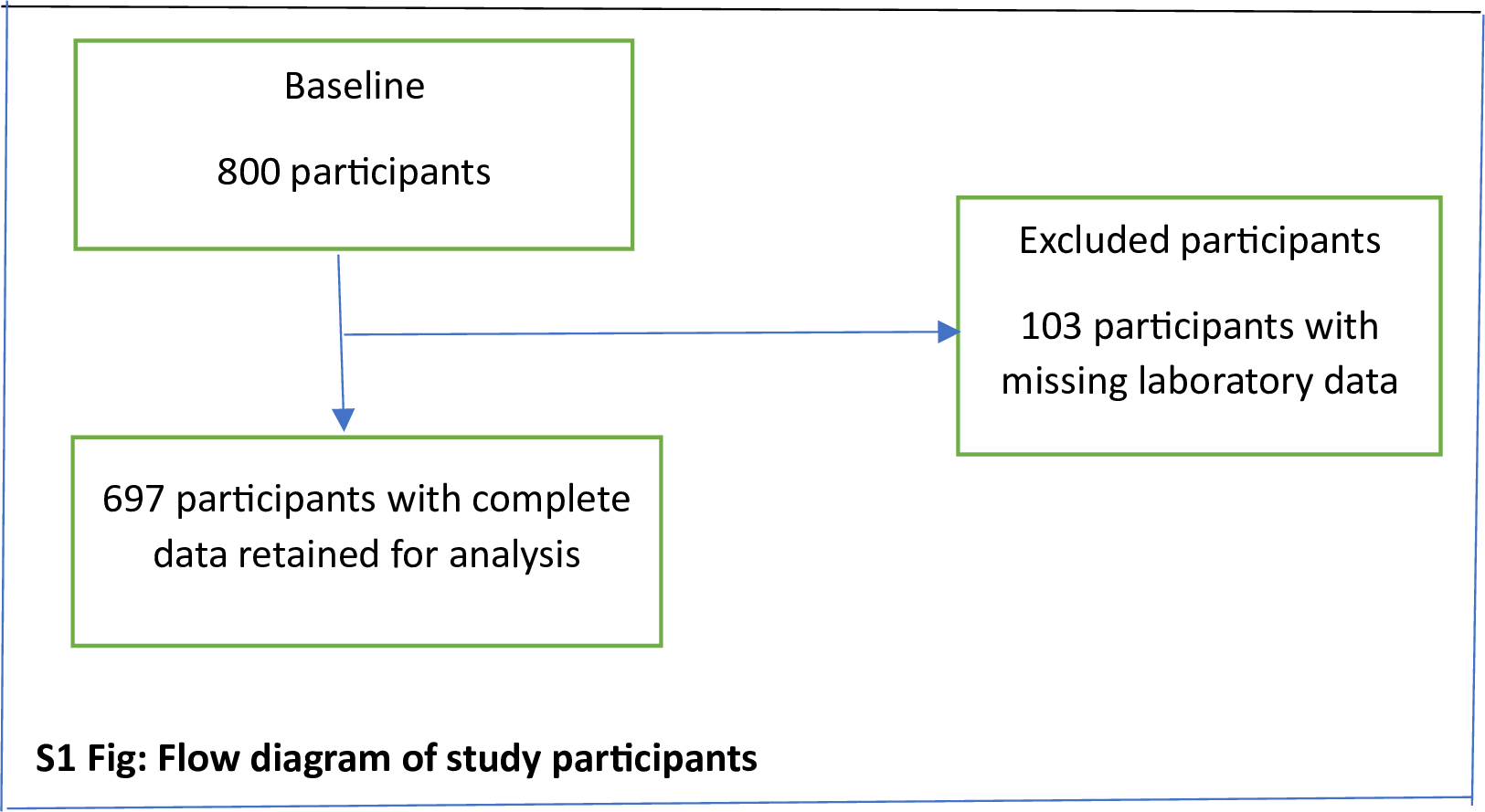

Supplement: S1 Fig — (TIF) [file pgph.0003059.s001.tif]

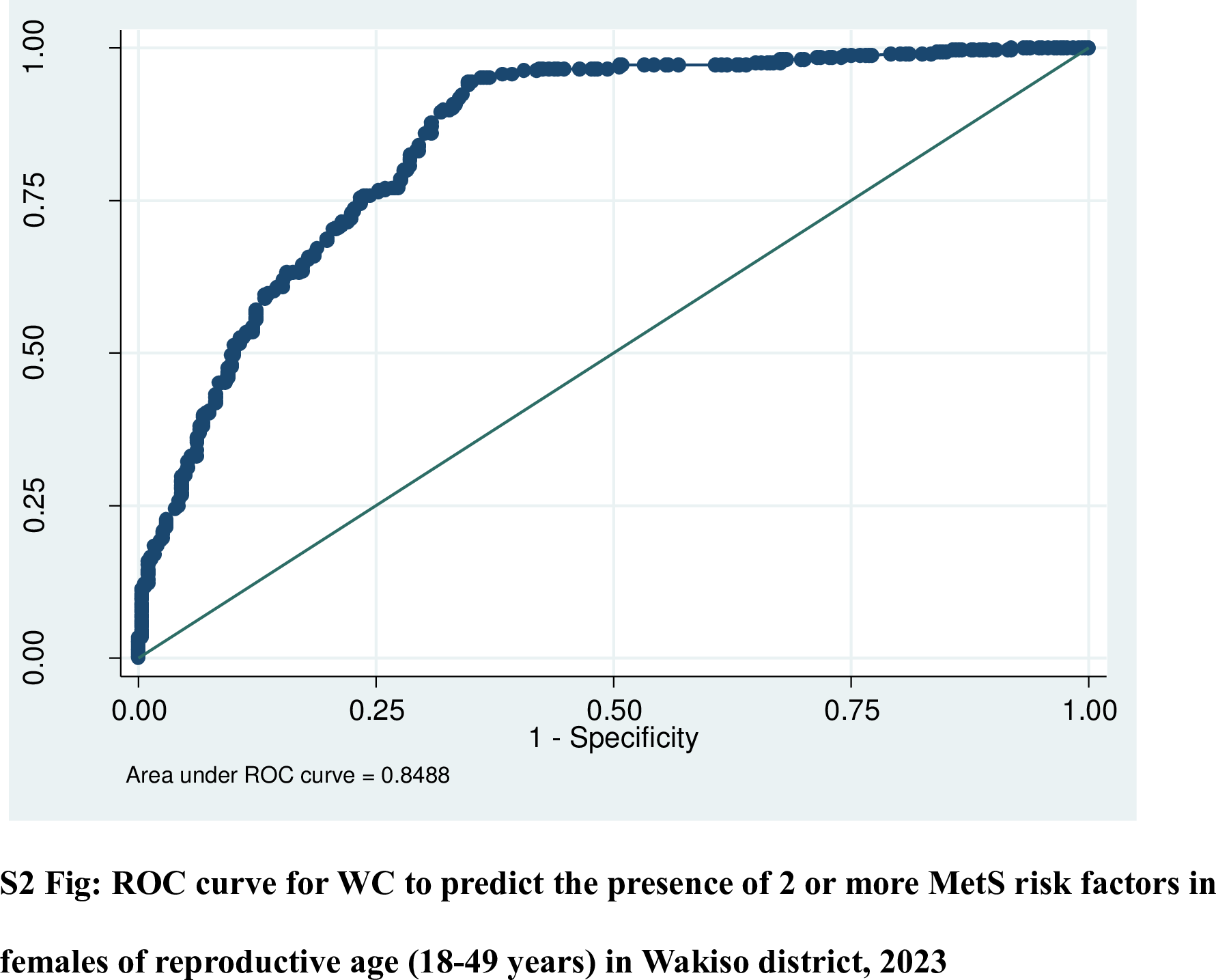

Supplement: S2 Fig — (TIF) [file pgph.0003059.s002.tif]

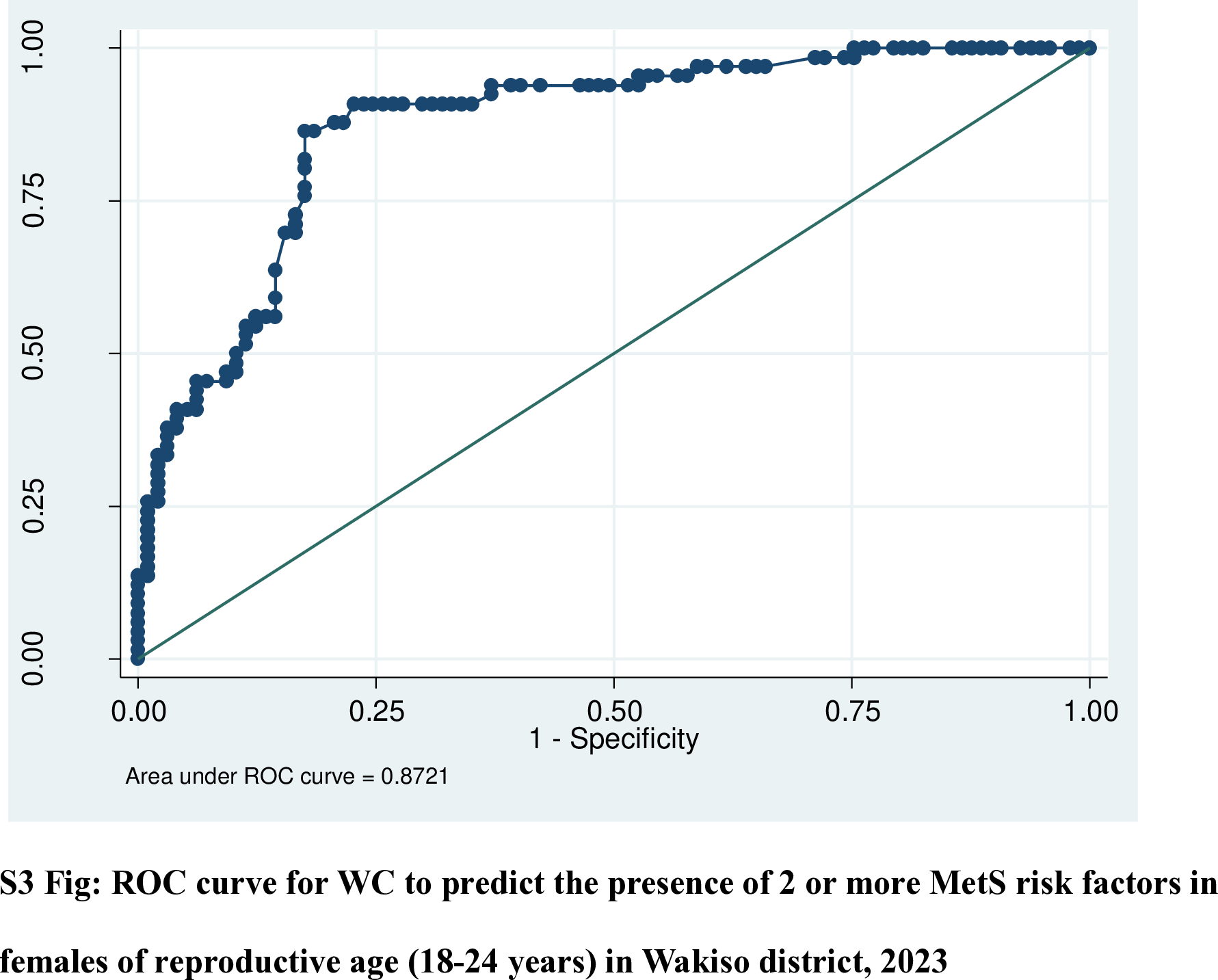

Supplement: S3 Fig — (TIF) [file pgph.0003059.s003.tif]

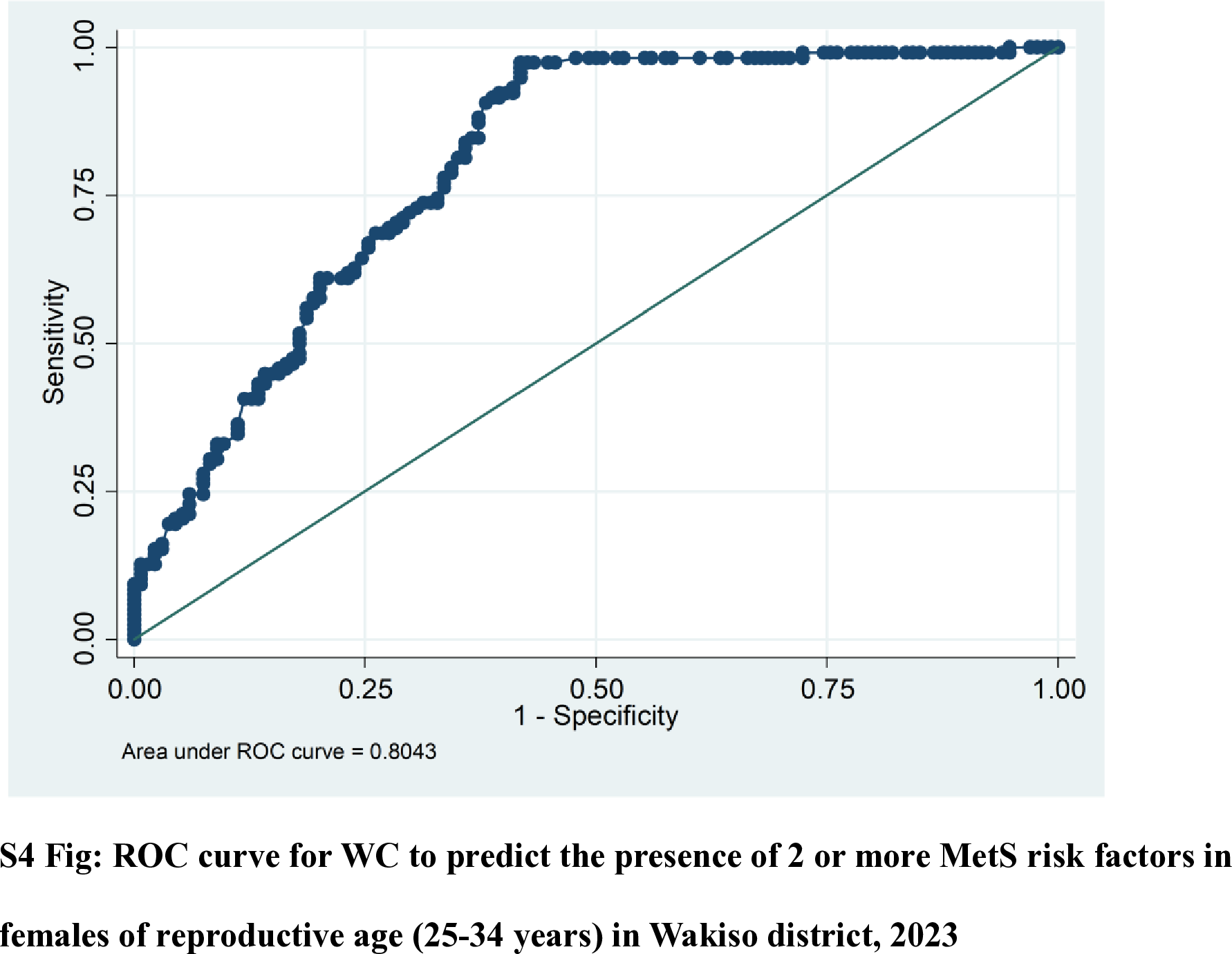

Supplement: S4 Fig — (TIF) [file pgph.0003059.s004.tif]

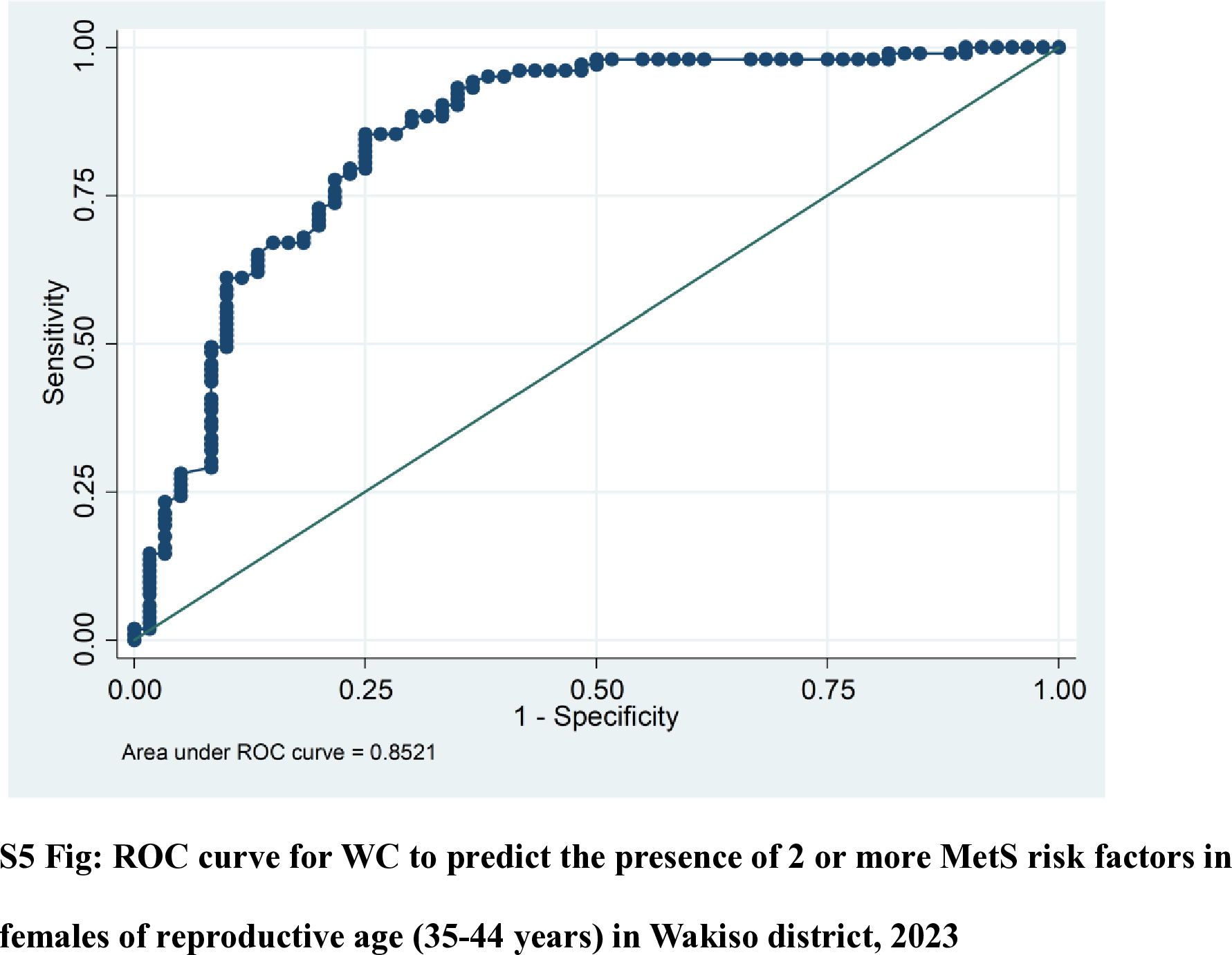

Supplement: S5 Fig — (TIF) [file pgph.0003059.s005.tif]

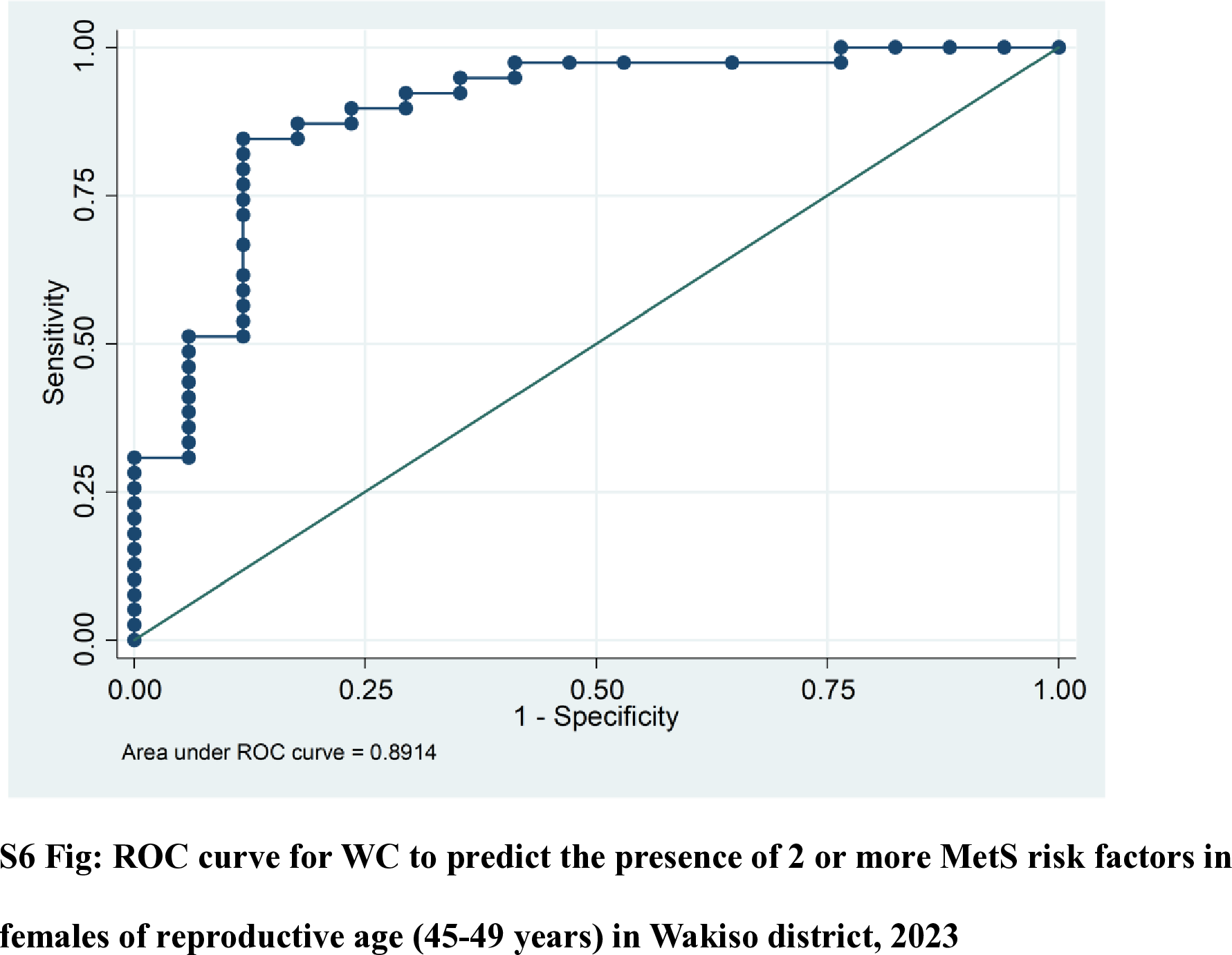

Supplement: S6 Fig — (TIF) [file pgph.0003059.s006.tif]
