# Supplementary material for: Optimal waist circumference cut-off points for predicting metabolic syndrome among females of reproductive age in Wakiso district, central Uganda
Source: PLOS Glob Public Health. 2025 Apr 9;5(4):e0003059. doi: 10.1371/journal.pgph.0003059 (PMC11981207; doi:10.1371/journal.pgph.0003059)
Supplement: Attachment — Submitted filename: 2. Response to Reviewers.pdf [file pgph.0003059.s011.pdf]

10th October 2024

Dear Editor and Reviewers,

We thank the Editor and the Reviewers for taking the time to assess our manuscript. We appreciate the detailed and insightful comments. A point-by-point response to the reviewer's comments is below

Response to Reviewer Comments

Abstract Corrections

Page 2, Lines 31-32:

The data collected were from a cross-sectional study conducted in Wakiso district, central Uganda, involving 697 randomly selected females aged 15 to 49, between June 9, 2021, and August 17, 2021.

Q1: Age Range Impact on Metabolic Syndrome

The study includes females aged 15 to 49, covering the reproductive age range. As metabolic risk increases with age, older participants are at higher risk. This broad age range could potentially dilute the overall prevalence of metabolic syndrome (MS) or alter the waist circumference (WC) cut-offs, as the younger age group typically has a lower risk compared to adults. Visceral fat distribution is significantly higher in adults than in teenagers or younger women, which may influence WC measurements and their association with MS. Additionally, hormonal changes and variations in body composition during different life stages, such as puberty and menopause, can affect fat distribution patterns, leading to varying MS risk profiles across age groups.

> ***Response**: Thank you. We agree with the reviewer, and we have narrowed the age range to 18-49 years in our calculations so that our results relate to other studies whose findings are comparable to adults. We also provided the 15-49 years for comparison purposes. For instance, page 2, lines 28 and page 3, line 45 and other areas in the manuscript as appropriate.*

Q2: Optimal WC Cut-offs

Page 2, Lines 39-41:

Variations in optimal WC thresholds were observed across different age groups: 79.4 cm for 15-24 years, 79.9 cm for 25-34 years, 85.6 cm for 35-44 years, and 91.1 cm for 45-54 years.

Q2a: It seems there may be a discrepancy; the correct value appears to be 79.4 cm rather than 97.4 cm.

> ***Response: We have corrected the discrepancy. The revised age range is 15-17 years, 18-24 years. 2-34 years, 35 -44 years, and 45 -49 years. We have also recalculated the respective optimal WC thresholds. The revised waist circumferences are 78.9 cm, 80.4 cm, 79.9cm, 85.6 cm and 91.1cm respectively. Page 2, lines 39-40.***

Q2b: Adulthood is generally defined as starting at 18 years of age. International standards set by the IDF, NCEP, and WHO provide waist circumference cut-offs primarily applicable to adults aged 18 years and older and do not include cut-offs for teenagers or adolescents. In this analysis, adolescents and adults aged 15-24 years were grouped together, which may affect the results for this group. Analyzing the 15-17 years age group separately could provide more accurate and relevant assessments.

> ***Response: We agree with the reviewer, and we have reanalysed the data and separated the adolescents (15-17 years) from the adults. The categorisation to 15-17 years yielded an optimal WC cut-off of 78.9 cm, while the optimal WC cut-off of the 15-24 years was 79.4 cm.***
>
> ***Interestingly, the overall optimal WC for 18-49 years did not change much remaining at 80.25 cm, with sensitivity of 94% and specificity of 65%. The overall optimal WC for 15-49 years was 80.25 cm, with a sensitivity of 94% and specificity of 67%. See page 14, table 2.***

Q3: Criteria for Metabolic Syndrome

Page 2, Lines 33-34:
MetS status was identified based on two or more MetS components excluding WC.

Q3a: Could you clarify the criteria used to exclude WC from MetS status? If you referred to any internal criteria, please provide the reference.

> ***Response: Thank you for the question. In this study, we excluded waist circumference from the MetS status criteria so that we could focus on its optimal cut-off points for diagnosing metabolic syndrome in the population under study. Including WC in the initial MetS classification would have introduced bias since we aimed to identify its specific contribution to metabolic risk independently of other established components. This approach allowed us to evaluate the WC diagnostic utility without confounding its role in MetS status. Our decision was guided by the internal study guidelines and literature that has used similar approaches. Please see page 2, lines 32-33 and a clarification on page 9, line 170-178.***

Q3b: According to Alberti et al. (2009), Metabolic Syndrome (MetS) should be classified based on the presence of any three abnormalities, including ethnic-specific waist circumference criteria. However, in your study, only two abnormalities were considered, and WC was excluded. This might lead to potential misclassification if an individual with a normal WC but two other abnormalities is classified as having MetS. Did you assess this potential misclassification by comparing your criteria with international standards?

> ***Response: Thank you for this question regarding the classification of MetS in our study. You are correct that in Alberti et al (2009), MetS is defined by the presence of any three abnormalities, out of the five., while using ethnic-specific WC criteria. However, in our study WC was excluded from the initial Mets criteria to allow for an objective determination of the optimal WC cut-off points specific to our population. The use of two abnormalities was part of our study design to focus on other metabolic components such as blood pressure, fasting blood glucose, triglycerides, and HDL cholesterol, while treating WC as the variable under investigation. This***

decision was taken to avoid circular reasoning, where WC would both define and be analysed in the same context.

We take note of the potential misclassification concern. Defining MetS based on the presence of two or more components, while excluding waist circumference, differs from established guidelines that require three or more components. This may lead to an overestimation of MetS prevalence in the population. We have acknowledged this fact in our study limitations. See page 22, lines 401-403.

"I recommend using the approach by Cook et al. (2003) and Xi et al. (2020), which defines central obesity in adolescents by the 90th percentile of waist circumference (WC). Analyzing adolescent data separately with this method could improve the accuracy of Metabolic Syndrome (MetS) risk assessment and provide valuable age-specific insights, enhancing the precision of your study's findings

Response:

Thank you for the references above. We were able to disaggregate our data and separate the 15-17 years age group from the adults (18 years and above). We analysed the adolescents separately and obtained their age-specific WC cut-off points as earlier indicated above. However, given that we had only one age category of adolescents (15-17 years), with the other four categories (18-24 years, 25-34 years, 35 -44 years and 45-49 years) in the adult category. We could not use the 90th percentile of waist circumference (WC), since most age categories are in the adult category. Thank you.

Q4: Enhancing the Introduction

Revised Text:
To enhance the depth and relevance of the introduction, consider including the following points:

1. "Obesity, with its associated metabolic and endocrine issues, can have a direct impact on women's reproductive health."
2. "Childhood obesity is a significant risk factor for developing metabolic diseases such as cardiovascular disease (CVD) and diabetes in later life."
3. "These conditions can exacerbate reproductive health issues, including polycystic ovary syndrome (PCOS), menstrual disturbances, and infertility."

Response: Thank you for these suggestions. We have incorporated them in our introduction. See page 5. Lines 94 to 99.

Q5: Inconsistency in MetS Criteria

Page 8, Lines 141-142:
Metabolic Syndrome was assessed by determining if an individual had three or more of the five components.

Response: Thank you for pointing out this. We have addressed it on page 9, line 170 to 178.

Page 2, Lines 33-34:
MetS status was identified based on two or more MetS components excluding WC.

Q5: The definitions of MetS in these two sentences differ, indicating an inconsistency. Please review and, if necessary, include a table highlighting the differences.

> ***Response: Thank you for pointing out this. We have addressed it on page 2 lines 32-33 and page 9, lines 170-178.***

Figures and Tables

Page 10, Line 181:

Q6. Is this figure S6 or S1?

> ***Response: Thank you. It is figure S1. We have corrected it on page 11, lines 207-208.***

Page 10, Lines 185-186: Table 1 indicates that almost half of the participants (49.9%) exhibited two or more risk factors linked to MetS (≥ 2 MetS factors).

Q7. Does this approach include Waist Circumference (WC) in the diagnosis, or was WC excluded?

> ***Response: This approach excludes waist circumference in the diagnosis of MetS. We have explained the reasons for this in our previous responses. Please refer to responses to Q3A and Q3B above.***

Table 2: There is a typographical error in the table for the age category 15-45.

Q8. Should this be 15-54?

> ***Response: Thank you for the question. We have corrected this to 18 to 49 following from your previous guidance. See Table 2 on page 14.***

Q9. Provide the number (n) for each category in the columns.

> ***Response: Thank you. The numbers are provided. Please refer to page 14 Table 2.***

Q10. Include the JIS and IDF criteria along with your current WC cut-off points (if any differences). Additionally, provide the prevalence percentage of each metabolic component overall and within each age category.

> ***Response: Thank you for this question. We agreed to include the IDF criteria definition in the introduction on page 4. However, we thought that our current WC cut-off points used 2 or more components for a definition of MetS, so we think that including the JIS and IDF which use 3 or more MetS components would not be appropriate.***

We have provided the overall prevalence percentage of each metabolic syndrome

Country [references]	n	Mean WC	Mean age of the study population	Cut-off point for women (cm
Uganda [24]	6136	77.5(MALE & FEMALE)	>=18 year	≥ 82 to ≥ 85
South Africa [19]	123	93.48±15.68	25–65 years	98
Benin [23]	452	88.6±13.6	25 to 60	94
**Angola [31]**	**615**	**83.9 ± 13.5**	**>20**	**80.5**
South Africa [18]	947	86.0 ± 13.4	>15 years	92
Benin [37]	541	87.6 ± 13.0	25- 60	90
South Africa [21]	152	-	24–65	94
South Africa [38]	1099	-	55–64 y	94
South Africa [22]	920	-	25–74-y	94.6
South Africa (present	998	-	≥ 18	≥ 89.45

component and within each age category on page 14, Table 2.

Optimal WC Cut-offs and Regional Variations in various African studies.

Page 14, Lines 222-226:

Our study has identified an optimal WC threshold value that closely approximates the recommended cut-off of 80.0 cm as specified by the International Diabetes Federation for diagnosing Metabolic Syndrome (MetS) (27). An optimal WC of 80.5 cm was found among female university employees in Angola (28).

Q11a: Except for the Angola study, optimal waist circumference (WC) cut-offs derived from various African regions suggest that the currently used 80 cm cut-off for women may overestimate abdominal obesity. Additionally, many of the studies included adults as volunteers, which may affect the results. It is advisable to recheck the analysis by either removing adolescents or providing a separate analysis for this group or do the age specific cutoff (use 90th percentile).

> ***Response: Thank you for the comment. We appreciate the concern on the overestimation of abdominal obesity using the current 80cm cut-off for women based on findings from various African regions. We re-analysed our data after removing the adolescents (15-17 years). The results of the group analysis to assess optimal WC for adults separately was reported and surprisingly, they did not differ much from the previous findings. See page 14, Table 2.***

Q11b It is my guess that the mean WC of your study population (86.4±13.8), including adolescents. The cut-off may be higher than the present 80.5 cm due to potential misclassification caused by the inclusion of adolescents. Please consider using age-specific categories and redoing the analysis. Kindly check.

> ***Response: Thank you. We confirm that we have reanalysed and checked as suggested. Page 14, Table 2.***

Elsewhere, some studies among a multiethnic Malaysian population (29) and in a Japanese population (30) found that 80.0 cm was an optimal WC for women.

Q12. However, these studies involved ethnically different populations compared to Africans. Comparing your findings with those of your ethnic population and justifying the differences by considering factors such as age, socioeconomic status, lifestyle, dietary patterns, physical activity, and urbanization could provide a more accurate assessment. In the future, conducting a meta-analysis could help derive ethnicity- and age-specific WC cut-offs for Africans.

> ***Response: We agree with the reviewer. We agreed to include a statement that the two cited studies were from an "ethnically different population compared to Africans". The rationale for retaining these studies in our work was to provide a broader perspective, as we have already cited studies from Africa and wanted to incorporate populations from outside the continent for comparison. See page 16. Line 273.***

Page 14, Lines 226-228:

The observed variation might be attributed to differences in factors such as genetics and body composition across different geographical regions. For instance, countries with higher prevalences of obesity, such as South Africa, often have higher optimal WC cut-off points (15, 33).

Q13. The variation in waist circumference (WC) cut-off points for defining central obesity across regions is influenced by genetics, body composition, and socio-environmental factors. For instance, countries with higher obesity rates, like South Africa, often have higher WC cut-offs due to greater body fat and different fat distribution. Economic conditions and urbanization also affect lifestyle and dietary patterns, impacting obesity rates and WC measurements. Including these factors in your discussion can enhance the understanding and interpretation of WC variations in your results.

> ***Response: Thank you for the insightful comments. We have incorporated the suggestions in the discussion. Please see page 17, lines 282-288.***

In Pages 15-18, Lines 249-250:

Probable reasons for the variation could be due to changes in body composition with age, resulting in reduced muscle mass and increased body fat, and a reduction in metabolic rate with age, leading to weight gain if dietary intake and physical activity remain the same. Do

you have data on physical activity and diet? If so, please include this information in your descriptive table. Add relevant references for these sentences.

In Pages 15-18, Lines 254-324:

> ***Response: Thank you for this comment. We have included the necessary reference on page 17, line 298. We have added data on physical activity and diet in the descriptive Table 1 as well.***

Q14. The discussion part repeats the results section, and the discussion points are limited. If your secondary objective is to focus on age and women health, then more information should be included in your manuscript. This will strengthen the discussion.

> ***Response: Thank you for the comment. We have beefed up the discussion points. See page 18, lines 290-295 and 19, lines 315-318 and***

We hope that the revised version of our manuscripts meets your expectations.

Kind regards

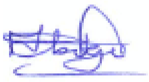

Dr David Lubogo

Corresponding Author.
