# Supplementary material for: Optimal waist circumference cut-off points for predicting metabolic syndrome among females of reproductive age in Wakiso district, central Uganda
Source: PLOS Glob Public Health. 2025 Apr 9;5(4):e0003059. doi: 10.1371/journal.pgph.0003059 (PMC11981207; doi:10.1371/journal.pgph.0003059)
Supplement: Attachment — Submitted filename: PGPH-D-24-00473_R1 with comments.pdf [file pgph.0003059.s010.pdf]

PLOS Global Public Health

Optimal waist circumference cut-off points for predicting Metabolic Syndrome among females of reproductive age in Wakiso district, central Uganda

--Manuscript Draft--

**Manuscript Number:**	PGPH-D-24-00473R1
**Article Type:**	Research Article
**Full Title:**	Optimal waist circumference cut-off points for predicting Metabolic Syndrome among females of reproductive age in Wakiso district, central Uganda
**Short Title:**	Optimal waist circumference cut-off points for prediction of Metabolic Syndrome in females
**Corresponding Author:**	David Lubogo, MBChB,MPH<br>Makerere University College of Health Sciences<br>Kampala, UGANDA
**Order of Authors:**	David Lubogo, MBChB,MPH
	Henry Wamani, MBChB, MPhil, PhD
	Roy William Mayega, MBChB, MPH, PhD
	Christopher Garimoi Orach, MBChB, DPH, MMed, MPH, PhD
**Keywords:**	Cut-off points; females; optimal; reproductive age; waist circumference
**Abstract:**	Background<br>Metabolic syndrome (MetS) poses a significant challenge to global public health, due to its strong association with Type 2 diabetes and cardiovascular disease. Waist circumference (WC) is a convenient metric for diagnosing MetS. Our study sought to establish waist circumference cut-off points that predict MetS among females of reproductive age in Wakiso district, central Uganda.<br>Methods:<br>The data collected were from a cross-sectional study conducted in Wakiso district, central Uganda, involving 697 randomly selected females aged 15 to 49, between 9th June 2021 and 17th August 2021. Data included MetS components: WC, High-Density Lipoprotein (HDL) Cholesterol, triglycerides, blood pressure, and fasting blood sugar. MetS status was identified based on two or more MetS components excluding WC. ROC analysis established the average optimal WC. The accuracy and performance of the cut-off points were evaluated through sensitivity, specificity, Positive likelihood ratio, and the Youden index.<br>Results:<br>Of the 697 participants, 50.1 % had two or more MetS risk factors. The average optimal WC cut-off for females aged 18-49 years was 80.3 cm. Variations in optimal WC thresholds were observed across different age groups: 78.9 cm for (15-17 years), 80.4 cm (18-24 years), 79.9 cm (25-34 years), 85.6 cm (35-44 years), and 91.1 cm (45-49 years) respectively. The area under the ROC curve at the cut point for these age groups ranged from 0.78 to 0.86, indicating good discriminatory capability. The sensitivity ranged from 85% to 97%, specificity from 58% to 88%, and the Youden Index from 0.557 to 0.729.<br>Conclusions:<br>A waist circumference of 80.3 cm was the optimal threshold for identifying metabolic syndrome in females between the ages of 18 and 49 years in the setting. This finding concurs with the guidance set forth by the International Diabetes Federation. Additionally, study participants had varied WC cut-offs ranging from 79.4 cm to 91.1 cm, depending on their age.
**Additional Information:**	
**Question**	**Response**
**Financial Disclosure**	DL received funding for this work from the Government of Uganda through the Makerere University Research and Innovation Fund (MakRIF) and the Strengthening Education and Training Capacity in Sexual and Reproductive Health and Rights (SET-

Powered by Editorial Manager® and ProduXion Manager® from Aries Systems Corporation

Enter a financial disclosure statement that describes the sources of funding for the work included in this submission and the role the funder(s) played. This includes grants and any commercial funding of the work or authors.<br><br>This statement will be typeset if the manuscript is accepted for publication.<br><br>*Please review the submission guidelines and the instructions link below for detailed requirements and guidance.*	SRHR) Project in Uganda. The funders had no role in study design, data collection and analysis, decision to publish, or preparation of the manuscript.
**Competing Interests**<br><br>On behalf of all authors, disclose any competing interests that could be perceived to bias this work.<br><br>This statement will be typeset if the manuscript is accepted for publication.<br><br>*Please review the instructions link below and PLOS Global Public Health's competing interests policy to determine what information must be disclosed at submission.*	The authors have no competing interests to declare.
**Data Availability**<br><br>Before publication, Authors are required to make fully available and without restriction all data underlying their findings. Please see our PLOS Data Policy page for detailed information on this policy.<br><br>A **Data Availability Statement**, detailing where the data can be accessed, is required at first submission. Insert your Data Availability Statement in the box below.	Data already provided as part of the submitted article.

Please see the data reporting section of our submission guidelines for instructions on what you need to include in your Data Availability Statement.<br><br>This statement will be typeset if the manuscript is accepted for publication.<br><br>*PLOS allows rare exemptions to address legal and ethical concerns. If you have legal or ethical restrictions, please use the box below to detail these in full sentences for the Journal team to consider.*	

Manuscript Click here to access/download;Manuscript;Manuscript.docx

Optimal waist circumference cut-off points for predicting Metabolic Syndrome among females of reproductive age in Wakiso district, central Uganda

Authors

David Lubogo[1*], Henry Wamani[1], Roy William Mayega[2], Christopher Garimoi Orach[1]

Affiliations

[1]Department of Community Health and Behavioural Sciences, School of Public Health, College of Health Sciences, Makerere University, Kampala, Uganda

[2]Department of Epidemiology and Biostatistics, School of Public Health, College of Health Sciences, Makerere University, Kampala, Uganda

* Corresponding author

Lubogodad@yahoo.com

Abstract

Background

Metabolic syndrome (MetS) poses a significant challenge to global public health, due to its strong association with Type 2 diabetes and cardiovascular disease. Waist circumference (WC) is a convenient metric for diagnosing MetS. Our study sought to establish waist circumference cut-off points that predict MetS among females of reproductive age in Wakiso district, central Uganda.

Methods:

The data collected were from a cross-sectional study conducted in Wakiso district, central Uganda, involving 697 randomly selected females aged 15 to 49, between 9th June 2021 and 17th August 2021. Data included MetS components: WC, High-Density Lipoprotein (HDL) Cholesterol, triglycerides, blood pressure, and fasting blood sugar. MetS status was identified based on two or more MetS components excluding WC. ROC analysis established the average optimal WC. The accuracy and performance of the cut-off points were evaluated through sensitivity, specificity, Positive likelihood ratio, and the Youden index.

Results:

Of the 697 participants, 50.1 % had two or more MetS risk factors. The average optimal WC cut-off for females aged 18-49 years was 80.3 cm. Variations in optimal WC thresholds were observed across different age groups: 78.9 cm for (15-17 years), 80.4 cm (18-24 years), 79.9 cm (25-34 years), 85.6 cm (35-44 years), and 91.1 cm (45-49 years) respectively. The area under the ROC curve at the cut point for these age groups ranged from 0.78 to 0.86, indicating good discriminatory capability. The sensitivity ranged from 85% to 97%, specificity from 58% to 88%, and the Youden Index from 0.557 to 0.729.

Given that the study's primary conclusion focuses on the 18–49 age range, could you clarify the purpose of including the 15–17 age group in your analysis?

Conclusions:

A waist circumference of 80.3 cm was the optimal threshold for identifying metabolic syndrome in females between the ages of 18 and 49 years in the setting. This finding concurs with the guidance set forth by the International Diabetes Federation.

Additionally, study participants had varied WC cut-offs ranging from 79.4 cm to 91.1 cm, depending on their age.

Keywords: Cut-off points, females, optimal, reproductive age, waist circumference

Introduction

Metabolic syndrome (MetS) corresponds to the combination of several disorders closely related to abdominal obesity (1), which is a strong predictor of adverse metabolic outcomes including insulin resistance, hypertension, and dyslipidemia (2, 3).

There are various definitions for MetS. However, according to the International Diabetes Federation (IDF), a diagnosis of MetS requires abdominal (central) obesity, measured by waist circumference, with ethnic-specific thresholds, ≥ 94cm and ≥ 90 cm for European and Asian men, respectively, and ≥ 80 cm for all ethnic groups in women, and at least two of the following four factors, reduced HDL Cholesterol, elevated triglycerides, high blood pressure, and elevated fasting blood sugar, (4-6).

Metabolic syndrome is a significant global public health challenge, contributing to high morbidity and mortality, and its occurrence is growing in Africa (7-10). The failure to address MetS significantly increases the chances of developing Type 2 diabetes and cardiovascular disease (11-13). Therefore, early identification of cases and management are key in preventing disease progression and the associated long-term health consequences (14).

The measurement of waist circumference (WC) serves as a simple and practical indicator for assessing abdominal obesity, which is a key component of metabolic syndrome (15, 16). The World Health Organization (WHO) and other scientific organizations strongly recommend using waist circumference measurement in screening, diagnosis, and routine clinical care to guide patient management (5, 17). This requires an acceptable waist circumference cut-off for risk stratification. However, establishing universally accepted WC cut-offs has been challenging due to differences in abdominal tissue composition among ethnic groups (18). This is because genetics, environmental, and lifestyle elements impact abdominal obesity (19) and overall body composition (20).

Waist circumference is a simple, useful and superior (21-23) predictor for MetS compared to body mass index. Its simplicity and ability to capture central obesity make it a widely used tool (24) in both clinical and public health practice. Abdominal adipose tissue positively correlates with waist circumference and waist-hip ratio and is associated with several metabolic abnormalities including adverse lipid profiles, decreased insulin sensitivity, and decreased glucose tolerance all of which are risk factors for T2DM and CVD.

The current waist circumference cut-off points for predicting metabolic syndrome vary by population and guidelines. For instance, according to the International Diabetes Federation (IDF) guidelines, the waist circumference cut-off for men is ≥ 94 cm for Europeans and ≥ 90 cm for South Asians, Chinese and Japanese. For women, the waist circumference is ≥80 cm for all ethnicities (24). The Sub-Saharan Africans use European waist circumference cut-offs (24). On the other hand, the WHO Expert Consultation (2008) cut-off points for men are ≥ 94 cm and ≥80 cm for women (25).

Metabolic syndrome is more prevalent among females than males (8, 11), directly affects their reproductive roles, and contributes to adverse maternal (26) and perinatal (27, 28) outcomes.

Women of reproductive age (15-49 years) experience distinct physiological and hormonal changes that influence their health and metabolic health (29). Hormonal fluctuations due to the menstrual cycle, pregnancy, and menopause, as well as reproductive health conditions such as polycystic ovary diseases (PCOS), can alter body fat distribution (30), leading to abdominal obesity. This abdominal obesity, along with its associated metabolic abnormalities, can directly affect women's reproductive health (31). Obesity in childhood can also lead to the development of metabolic diseases such as diabetes or cardiovascular disease later in life (32).

Uganda and other countries in sub-Saharan Africa use WC cut-offs endorsed by international scientific organizations including the IDF and WHO (4, 20). Nevertheless, it is still unclear whether established thresholds, such as the ≥ 80 cm suggested for females, are valid and

appropriate for the African population. Hence, it the necessary to conduct research on the diverse African population groupings including Uganda and to establish sex-specific WC cut-offs as recommended by WHO and others (5, 20).

To date, most studies on optimal waist circumference cut-off points have been conducted in high-income countries. In low-income countries such as Uganda, the optimal ethnic WC cut-off has not been established particularly for females of reproductive age. Given the unique metabolic challenges faced by women of reproductive age, determining optimal WC cut-off points that are sensitive to their specific physiological context is warranted to improve early detection and management of the condition.

Therefore, this study aimed at determining the average waist circumference and age-specific cut-off points that are optimal for predicting MetS among females of reproductive age in Wakiso district, central Uganda. This may assist in the early detection, prevention and management of metabolic syndrome. The hypothesis for the study was that the optimal waist circumference for females aged 15-49 years might not be equal to 80.0cm.

Methods

Study design

We carried out a cross-section study in Wakiso district between 9th June 2021 and 17th August 2021. Our study involved a random sample of female participants from 15 to 49 years of age.

Study population

The study population included females aged 15-49 years residing in Wakiso district, central Uganda. Participants were randomly selected from urban and rural communities to ensure diversity. Eligible participants had resided in Wakiso for at least a year and were identified from a database of 800 individuals, who had anthropometric, physical, and biochemical tests carried out on them to determine the prevalence of MetS in the district.

We excluded participants who were pregnant, those within six weeks after childbirth, **alcoholics, smokers,** those with chronic diseases such as diabetes mellitus, hypertension, hyperlipidaemia, allergic disorders, depression, and those with known carrier states of hepatitis B, hepatitis C, or human Immunodeficiency virus.

Study setting

The study was conducted in Wakiso district, situated in central Uganda, with an estimated total population of 3,105,700, based on the 2014 census. More than half (52.7%) of the residents are females. Of these, 793,770 are in the reproductive age bracket of 15-49 years (33). The district comprises two counties, namely Kyaddondo and Busiro, and four Municipal Councils - Entebbe, Nansana, Kira, and Makindye-Ssabagabo that together constitute eight sub-counties, eight town councils, 148 parishes, and 704 villages. The Uganda Bureau of Statistics provided the sampling frame for the study, and it included 32 randomly selected enumeration areas in the district. Twenty-eight (28) households were sampled per enumeration area.

Sample size calculation

The sample size was computed using the OpenEpi, Version 3.01, open-source calculator for a proportion or descriptive study (34). Using a population size for a finite population of 1,000,000, confidence limits of 5%, a design effect of 2.0., and a prevalence (p) of 40.2% +/-5 obtained from a study conducted among females in an urban population in Kenya to determine MetS prevalence (35). A sample size of 739 was obtained; after adjusting for a response rate of 90%, the sample size was 813. The final sample used in the study was 800 respondents. We estimated a MetS prevalence of 15% and expected 120 respondents to be with MetS out of the total sample size of 800. This sample was considered appropriate for assessing the sensitivity and specificity of WC as a predictor for MetS in this sample.

Measurements and variables

We used the population sample data set from a prevalence study to obtain participant data on anthropometric measurements (waist circumference), physical measurements (blood pressure), and biochemical measurements (fasting plasma glucose, triglycerides, and low HDL Cholesterol) collected as per the WHO STEP wise approach (36).

Quality control measures were undertaken to ensure the data for the prevalence study were of quality. They included the employment of well-trained data collectors/research assistants (female nurses and graduate nutritionists) who were knowledgeable of the study protocol and had skills in using the Open Data Kit (ODK) for data collection. The data collection tools were field tested from a district near the study district. The data collected in the field were entered in the ODK tool embedded with skip patterns and constraints to ensure data quality and reduce bias. Quantitative data (ODK data) was downloaded from the server and exported to Stata version 14 for cleaning. This ensured reasonable quality control and data management for this study.

Study variables

Dependent variable:

Metabolic syndrome is assessed by determining if an individual has three or more of the five components namely: low HDL cholesterol (<50 mg/dL or (1.3 mmol/l) in female) or treatment for it, high triglycerides (≥150 mg/dL (1.7 mmol/L) or treatment for it, elevated fasting blood sugar (≥ 100 mg/dL (5.6 mmol/l) or diabetes diagnosis, or diabetes treatment; high blood pressure (≥130/ ≥85 mmHg) or treatment for it, and increased waist circumference. These components are derived from the Joint Interim Statement (JIS) (5). This study assessed MetS using two of the four metabolic components, excluding waist circumference. This approach was used to evaluate the independent contribution of waist circumference to metabolic risk. Similar methods have been applied in previous studies (37-39).

Data analysis

Data were analysed using Stata statistical software (SE/14.0, StatCorp, College Station, TX, USA (40). Descriptive statistics were employed to summarise participant characteristics. Data on continuous variables were summarised using the independent sample T-test and categorical variables were analysed using the Chi-square test. The results were presented as means with standard deviation and frequencies/percentages.

Waist circumference cut-off points for various age groups (15-17, 18-24, 25-34, 35-44, 45-49, 15-49, 18-49, years) were evaluated using sensitivity and specificity techniques to identify the most effective predictive values for MetS within the study age groups.

The research investigated the Area under the Curve (AUC) for different components of MetS such as elevated fasting blood sugar, high triglycerides, high blood pressure and low HDL cholesterol. To conduct this analysis, an ROC curve was plotted for the association between waist circumference (WC) and two or more components of MetS.

The optimal cut-off points for WC, which achieve maximum accuracy, were determined by plotting sensitivity and (1-specificity) on a graph.

The study employed the Youden index method (41) expressed as [Maximum (sensitivity + specificity - 1)] to ascertain the optimal WC values and also calculated the positive predictive values (PPV) and negative predictive values (NPV).

Ethical considerations

We obtained approval from the Higher Degrees Research and Ethics Committee of the School of Public Health, College of Health Sciences, Makerere University (Protocal number: MakSPH-REC, 071) and the National Council for Science and Technology of Uganda (Registration number: UNCST, HS1281ES). All study participants provided written informed consent and were assured of the confidentiality of their information. Written informed consent was obtained from parents or guardians of participants under 18 years of age.

Results

Flow of participants

The figure in S1 Fig shows the flow diagram of the study participants.

S1 Fig: Flow diagram of study participants

Characteristics of study participants

The study involved 697 women of reproductive age with a mean age of 29.7 ± 9.07 years. Most participants (38.7%) completed primary school, and 51.5% were employed. The mean body mass index (BMI) was 26.9 (±5.8), 43% of the participants had a normal BMI, and 27.6% were obese. See Table 1.

Table 1: Characteristics of women of reproductive age in Wakiso district (N=697)

Kindly give the mean and SD for all the age categories (15-17,18-24,25-34,35-44,45-49)

Characteristics	N	Percent
All participants	697	100.0
Age in years (mean, SD)	29.7 (SD=9.07)	
Level of Education attained		
Primary not completed	166	23.8
Primary School	270	38.7
0-Level	154	22.1
A-Level or above	107	15.4
Employment Status		
Paid and employed	359	51.5
Unpaid and Unemployed	338	48.5
BMI[a] (kg/m^2) (Mean, SD)	26.9 (SD=5.8)	
< 18.5	20	2.9
18.5–24.9	300	43.0
25.0–29.9	185	26.5
≥ 30	192	27.6
Fruits and vegetables consumed per day		
< 5 servings	488	70.0
≥ 5 servings	209	30.0
Physical Activity		
Insufficient physical activity	550	78.9
Moderate physical activity	61	8.8
Vigorous physical activity	86	12.3
Current alcohol users (Past 30 days)		
Non-current drinker	605	86.8
Lower end level (< 2 drinks /occasion)	27	3.9
Intermediate end level (2 - < 4 drinks per occasion)	52	7.4
High-end level ≥4 drinks per occasion)	13	1.9
Tobacco Use		
Non-smokers (past 30 days)	689	98.9
Smokers (past 30 days)	8	1.1
Dietary Salt		
Adding salt or salty sauce at meal to food before or when eating		
Sometimes - never	571	82.0
Always or often	126	18.0

Please provide some foot-note for the level of education, - A level and physical activity definition

Physical Activity Levels: Specify the classification method, e.g., WHO guidelines, PAL, or GPAQ.
Alcohol Use Levels: Define low, intermediate, and high levels, and mention the measurement standard used.
BMI Classification: Use standard WHO categories. Since adolescents are included in Table 1, apply age- and sex-specific BMI percentiles for them per WHO guidelines or consider removing adolescents from the analysis. In Table 2, for ages 15–17, the AUC of 0.81 suggests good performance, though the wide confidence interval (0.690–0.937) may reflect the small sample size (n=63). Sensitivity (87%) is high, but specificity (75%) and the Youden index (0.620) are moderate. Larger sample sizes in future studies could enhance these results.

Age-specific evaluation of optimal thresholds for waist circumference using different classification metrics and prevalence of MetS components

For the age group 15-45 years, the optimal threshold was 80.25 cm. The Youden index, specificity and sensitivity were 0.604, 67% and 94% respectively, and the AUC curve was 0.848 (95% CI 0.819 - 0.877), while for 18-45 years, the optimal threshold was 80.25 cm. The Youden index, specificity and sensitivity were 0.591, 65% and 94% respectively, and the AUC curve was 0.843 (95% CI 0.812 – 0.875).

The optimal WC cut-off points varied among the various age groups, ranging from 78.9 cm for females aged 15 to 17 years, to 91.1 cm for those aged 45 to 49 years. Across different age groups, the area under the ROC curve at cut-point values fluctuated between 0.78 and 0.86, with the 45- 49-year-old age group showing the highest AUC of 0.86. The 45-49 years age group showed the highest discriminative power (AUC =0.891), with specificity (88 %), Youden index (0.729), and a likelihood ratio of (7.08).

The prevalence of various MetS components increased with age, particularly elevated waist circumference and blood pressure. Reduced HDL cholesterol appeared consistent across all age groups as shown in Table 2.

Table 2: Age-stratified waist circumference cut-off points, MetS risk classification metrics, and prevalence of MetS components by age group.

**Variable**	**Age group**						
	**15-17 years(n=63)**	**18-24 years (n=163)**	**25-34 years (n=252)**	**35-44 years (n=163)**	**45-49 years(n=56)**	**15-49 years(n=697)**	**18-49 years(n=634)**
**MetS risk Classification metrics**							
Empirical optimal WC cut-off point (cm)	78.9	80.4	79.9	85.6	91.1	80.3(80.25)	80.25
Area under the ROC curve at the cut point	0.81	0.84	0.78	0.80	0.86	0.80	0.80
Area under ROC curve (95% CI)	0.814 (0.690 - 0.937)	0.872 (0.817 -0.927)	0.804 (0.750 - 0.858)	0.852 (0.786 - 0.918)	0.891 (0.790 - 0.993)	0.848 (0.819 - 0.877)	0.849 (0.819 - 0.879)
Sensitivity at cut point (%)	87	86	97	85	85	94	94
Specificity at cut point (%)	75	82	58	75	88	67	65
Youden index (J)	0.620	0.688	0.557	0.604	0.729	0.604	0.597
LR+ = sensitivity / (1 - specificity)	3.480	4.778	2.3095	3.40	7.08	2.85	2.66
Presence of 2 or more metabolic syndrome risk factors (%)	36.5	40.5	46.8	63.2	69.6	50.1	51.4
**Metabolic syndrome components**							
Elevated waist circumference (%)	35.9	48.0	66.8	77.3	82.8	63	66.1
Elevated triglycerides (%)	10.1	13.1	12.3	15.9	24.4	14.1	14.6
Reduced HDL Cholesterol (%)	44.7	48.9	48.6	47.0	41.2	47.3	47.6
Elevated blood pressure (%)	14.0	21,0	19.5	35.2	42.8	25.0	26.2
Elevated fasting blood sugar (%)	4.1	11.7	5.0	6.8	6.7	7.1	7.4

ROC curve analysis, for waist circumference in reproductive-age women in Wakiso district, for detecting MetS risk factors per age group

Figures in S2-S7 Figs show the ROC curves for WC to predict the presence of 2 or more **MetS risk factors among females of reproductive age for the various age groups.**

S2 Fig: ROC curve for 18-49 years old females in Wakiso district, 2023

S3 Fig: ROC curve for 15-17 years old females in Wakiso district, 2023

S4 Fig: ROC curve for 18-24 years old females in Wakiso district, 2023

S5 Fig: ROC curve for 25-34 years old females in Wakiso district, 2023

S6 Fig: ROC curve for 35-44 years old females in Wakiso district, 2023

S7 Fig: ROC curve for 45-49 years old females in Wakiso district, 2023

Discussion

The rise in the global prevalence of MetS calls for more research towards early and accurate diagnosis of MetS to provide preventive measures and timely intervention for MetS.

Our study revealed that the mean waist circumference cut-off points of 80.3 cm accurately predicted MetS in females aged 18-49 years in an urban population in Uganda. However, optimal waist circumference varied by age groups (15-17 years, 18-24 years, 25-34 years, 35-44 years, and 45-49 years) being 78.9 cm, 80.4 cm, 79.9 cm, 85.6 cm, and 91.1 cm respectively. This study is the first to use the Joint Interim Statement to establish these cut-off thresholds in Uganda.

The findings showed a strong link between age and the prevalence of several MetS components, particularly elevated waist circumference and blood pressure, both of which increased with age. Reduced HDL cholesterol appeared consistent across all age groups. Public health interventions for preventing metabolic syndrome should consider targeting abdominal obesity and hypertension, especially for older individuals within the reproductive age range. Research indicates that obesity (42) and hypertension (43) impact maternal and neonatal health.

Have you conducted inferential statistical tests, such as ANOVA, for Table 2? This analysis is essential to validate the findings

Our study has identified an optimal WC threshold value that closely approximates the recommended cut-off of 80.0 cm as specified by the International Diabetes Federation for diagnosing metabolic syndrome (MetS) (44). Similar results were found in prior studies conducted across Africa and other locations. An optimal WC of 80.5 cm was found among female university employees in Angola (45). Elsewhere, some studies among a multiethnic Malaysian population (46) and in the Japanese population (47) found that 80.0 cm was an optimal WC for women. However, it should be noted that these two studies were conducted among ethnically different populations compared to Africans. The consistency of our findings with those of other researchers strengthens the validity of our findings and reinforces the global

relevance of the established diagnostic criteria. From a public health perspective, policymakers and healthcare professionals may utilize these findings to implement cost-effective and efficient screening programs to enable the early detection and management of MetS.

Our findings, however, differed from a study in Ethiopia, which revealed the optimal WC at 78.0 cm (48). Higher optimal WC cut-off points of ≥ 82.3 cm and 92.0 cm were found in studies conducted among women in Botswana and South Africa, respectively (15, 49). These variations highlight the importance of considering region-specific factors while establishing WC cutoff points for assessing women's health. The observed variation might be attributed to differences in factors such as genetics, body composition, and socio-environmental factors across different geographical regions. For instance, countries with higher prevalences of obesity, such as South Africa, often have higher optimal WC cut-off points (15, 50) due to greater fat and different fat distribution. Economic conditions and urbanisation also affect lifestyle and dietary patterns (51), impacting obesity rates and waist circumference measurements.

This study also revealed varying age-specific WC thresholds for various age groups: 78.9 cm for 15-17 years, 80.4 cm for people aged 18-24 years, 79.9 cm for those aged 25-34 years, 85.6 cm for the 35-44 years age group, and 91.1 cm for those aged 45-49 years respectively. The age-specific cut-off points highlight the variability of MetS risk across various age groups. The younger individuals showed risk factors even with slightly lower waist circumference levels. This emphasizes the importance of early detection and timely intervention in this group. Conversely, elderly individuals exhibited higher waist circumference cut-off points. This is in agreement with other studies (52). Probable reasons for this could be due to changes in body composition with age resulting in reduced muscle mass and gain in body fat, and a reduction in metabolic rate (53) which occurs with age resulting in weight gain if dietary intake and physical activity remained constant.

Therefore, to effectively combat the rising burden of cardiovascular diseases, healthcare providers and policymakers should include age-specific thresholds for waist circumference in screening and intervention protocols for metabolic syndrome.

Diagnostic accuracy of the waist circumference cut-off points to detect MetS risk factors

Different metrics were employed to assess the precision of detecting metabolic syndrome, including AUC, PPV, specificity and sensitivity, with reference to a specific waist circumference threshold.

When we measured the performance of the AUC values within the 15-17 years and the 18-24 age range, the AUC reached 0.81 and 0.84, respectively. This data suggests that the WC cut-off point can serve as a reasonably accurate indicator of MetS risk in individuals within this age range. For the 25-34 years age group, the AUC was 0.78, this demonstrates moderate performance. In the 35-44 years age category, the AUC was 0.80, this suggests a relatively good ability to distinguish those at risk for MetS. In the 45-49 years age group, the AUC was 0.86. This age group exhibited the highest AUC values, and thus the most robust discriminative power. This suggests that this group may be the most appropriate target for MetS screening using WC. Considering all individuals aged 15-49 years or 18-49 years, the overall AUC was 0.80, indicating good performance in identifying MetS. Normally, an AUC of 1 indicates a perfect classifier with the model yielding a 100% true positive rate and 0% false positive rate across all possible threshold values. An AUC of 0 shows that the classifier performs no better than chance, is not able to distinguish between two classes and yields a 0% true positive rate and 100% false positive rate (54). Most classifiers aim for an AUC above 0.5, and that indicates predictive power.

These findings hold important implications for healthcare professionals and researchers, as they provide valuable guidance for developing protocols for screening MetS that are specific to different age groups.

Effectiveness of waist circumference cut-off points in terms of sensitivity and specificity

For the 15-17 age group, a waist circumference of 78.9 cm was associated with 87% sensitivity and 75% specificity. The high sensitivity suggests that it is effective in identifying metabolic syndrome. However, the moderate specificity indicates some potential for false positives in that age group.

Considering sensitivity and specificity values, in the 18-24 age group, a WC of 80.4 cm was obtained with 86% sensitivity and 82% specificity. The high sensitivity also suggests that it is effective in identifying metabolic syndrome.

The 25-34 age group showed a WC cut-off of 79.9 cm and had the highest sensitivity of 97%. This means that nearly all individuals with metabolic syndrome in this age group are accurately identified. However, a lower 58% specificity may imply concerns about potential false positives, and thus there is a need to do confirmation tests.

The 35-44 years age category had an 85.6 cm cut-off, and balanced sensitivity (85%) and specificity (75%). This implies that it is reasonably practical in identifying those with metabolic syndrome and can minimize the risk of false positives, making it a valuable tool for screening.

In the 45-49 age group, there was a 91.1 cm cut-off point, with an 85% sensitivity and 88% specificity. This implies that this cut-off can accurately identify individuals at risk and can minimise false positives. For the age range of 15-49 years, and 18-49 years, we obtained an 80.25 cm cut-off point, with 94% sensitivity and 67% specificity, and 94% sensitivity and 65% specificity, respectively. This shows that has the potential to serve as an efficient screening

tool. However, the lower specificity of 65% and 67% calls for further evaluation to confirm diagnoses.

Generally, these age-specific WC cut-off points can be used to offer tailored strategies for MetS risk assessment and highlight the importance of age in risk prediction and patient management. Further research may be needed to validate and expand these findings for broader clinical and public health applications.

Youden Index and Positive Likelihood Ratios (LR+)

Our study evaluated age-specific waist circumference (WC) cut-off points and their associated Youden index (J) and Positive Likelihood Ratios (LR+) to determine the diagnostic performance across different age groups.

Our study identified distinctions among the different age categories. The 45-49-year-old age group showed the highest Youden index (J) of 0.729, indicating a higher overall accuracy than the other age groups. A value of one (1) indicates perfect accuracy, and the closer J is to 1, the better the test's overall performance (55). Furthermore, it showed the highest LR+ value of 7.08. This implies that among individuals within this age group, a positive test result is about 7.08 times more likely to occur in individuals with metabolic syndrome compared to those without the syndrome. This makes it a robust diagnostic tool.

The 25-34 years age group showed a slightly lower Youden index (J) of 0.557 (indicating slightly lower overall accuracy) compared to the 18-24 years group with a Youden index (J) of 0.688. The 25-34 years age group showed an LR+ of 2.3095, demonstrating valuable diagnostic utility. A LR+ of 2.3095 suggests that individuals diagnosed with metabolic syndrome have a waist circumference above the cut-off point approximately 2.3 times more often than those without metabolic syndrome. Although this LR+ was slightly lower than the 18-24 years group LR+ (Likelihood Ratio Positive) of 4.788, this LR+ is still a valuable indicator of diagnostic

strength. The 35-44 years and the broader 15-49 years age range both demonstrated intermediate Youden index (J) values of 0.604, with corresponding LR+ values of 3.4 and 2.85, respectively.

The study demonstrates that for urban Ugandan females aged 18-49 years, 80.3 cm waist circumference is the mean optimal threshold for MetS prediction. However, these findings were based on data from one district and should be interpreted cautiously when generalizing to other populations. Furthermore, waist circumference cut-off points varied depending on age group as indicated; 78.9 cm (15-17 years),80.4 cm (18-24 years), 79.9 cm (25-34 years), 85.6 cm (35-44 years), and 91.1 cm (45-49 years). Our findings provide useful information for healthcare professionals when considering age-tailored risk assessment strategies for metabolic syndrome to enhance the clinical relevance of WC measurements.

Study limitations

We used a cross-sectional study design. MetS is a condition that develops over time, and the measurement of WC in a single time point may not fully capture its dynamic nature. A longitudinal study would be more appropriate for confirming the temporal relationships observed in the study findings.

Our study focused exclusively on females of reproductive age in Wakiso district, central Uganda. Consequently, while the findings are relevant to women in a similar setting, the results may not be generalizable to the entire Ugandan population. Conducting nationally representative studies on optimal waist circumference in the future would help address this limitation and provide a better understanding of MetS in Uganda.

Our study sample was randomly sampled from an existing database. While random sampling ensures diversity, it may miss out on segments of the population not included in the database. This may have led to a potential selection bias.

In the computation of the sample size for our study, we used a prevalence of MetS obtained from a study among females in an urban population in Kenya. This may not accurately reflect the current study population characteristics. However, given that our population is female and in an urban setting, we think this limitation was minimised.

Defining MetS based on the presence of 2 or more components while excluding waist circumference, unlike using 3 or more components as in established guidelines can result in overestimation of the prevalence of MetS in the population.

Conclusions and recommendations

We found that a waist circumference of 80.3 cm was the mean optimal threshold for identifying metabolic syndrome in females between the ages of 18 and 49 years in Wakiso district, central Uganda. Furthermore, participants exhibited waist circumference cut-offs ranging from 78.9 cm to 91.1 cm based on age group. This study highlights the significance and precision of the existing waist circumference guidelines in healthcare. This study calls for the utilization of a waist circumference cut-off threshold to predict metabolic syndrome among reproductive-age females in Uganda, based on age group. The findings of this research may be relevant to healthcare providers, policymakers, public health practitioners and researchers. Further research in a wider range of populations encompassing both rural and urban settings is needed to validate these waist circumference cut-off points.

Acknowledgements

We appreciate all the study participants and research assistants who supported the data collection process. We thank all the local leaders and the district health management team members in Wakiso district who facilitated the research teams. We appreciate Dr Arthur Bagonza (PhD) and Dr Freddie Kitutu (PhD) for reviewing the manuscript.

References

1. Paley CA, Johnson MI. Abdominal obesity and metabolic syndrome: exercise as medicine? BMC Sports Science, Medicine and Rehabilitation. 2018;10:1-8.
2. Bovolini A, Garcia J, Andrade MA, Duarte JA. Metabolic syndrome pathophysiology and predisposing factors. International Journal of Sports Medicine. 2021;42(03):199-214.
3. Wang HH, Lee DK, Liu M, Portincasa P, Wang DQ-H. Novel insights into the pathogenesis and management of the metabolic syndrome. Pediatric gastroenterology, hepatology & nutrition. 2020;23(3):189.
4. Alberti KGMM, Zimmet P, Shaw J. Metabolic syndrome—a new world-wide definition. A consensus statement from the international diabetes federation. Diabetic medicine. 2006;23(5):469-80.
5. Alberti K, Eckel RH, Grundy SM, Zimmet PZ, Cleeman JI, Donato KA, et al. Harmonising the Metabolic Syndrome a Joint Interim Statement of the International Diabetes Federation Task Force on Epidemiology and Prevention; National Heart, Lung, and Blood Institute; American Heart Association; World Heart Federation; International Atherosclerosis Society; and International Association for the study of Obesity. Circulation. 2009;120(16):1640-5.
6. Alberti G, Zimmet P, Shaw J, Grundy SM. The IDF consensus worldwide definition of the metabolic syndrome. Brussels: International Diabetes Federation. 2006:1-23.
7. Okafor CI. The metabolic syndrome in Africa: Current trends. Indian journal of endocrinology and metabolism. 2012;16(1):56-66.
8. Bowo-Ngandji A, Kenmoe S, Ebogo-Belobo JT, Kenfack-Momo R, Takuissu GR, Kengne-Nde C, et al. Prevalence of the metabolic syndrome in African populations: A systematic review and meta-analysis. PloS one. 2023;18(7):e0289155.

9. Saklayen MG. The global epidemic of the metabolic syndrome. Current hypertension reports. 2018;20(2):1-8.

10. Noubiap JJ, Nansseu JR, Lontchi-Yimagou E, Nkeck JR, Nyaga UF, Ngouo AT, et al. Geographic distribution of metabolic syndrome and its components in the general adult population: A meta-analysis of global data from 28 million individuals. Diabetes research and clinical practice. 2022;188:109924.

11. Faijer-Westerink HJ, Kengne AP, Meeks KA, Agyemang C. Prevalence of metabolic syndrome in sub-Saharan Africa: A systematic review and meta-analysis. Nutrition, Metabolism and Cardiovascular Diseases. 2020;30(4):547-65.

12. Hwang Y-C, Jee J-H, Oh EY, Choi Y-H, Lee M-S, Kim K-W, et al. Metabolic syndrome as a predictor of cardiovascular diseases and type 2 diabetes in Koreans. International journal of cardiology. 2009;134(3):313-21.

13. Mohamed SM, Shalaby MA, El-Shiekh RA, El-Banna HA, Emam SR, Bakr AF. Metabolic syndrome: risk factors, diagnosis, pathogenesis, and management with natural approaches. Food Chemistry Advances. 2023;3:100335.

14. Handelsman Y, Butler J, Bakris GL, DeFronzo RA, Fonarow GC, Green JB, et al. Early intervention and intensive management of patients with diabetes, cardiorenal, and metabolic diseases. Journal of Diabetes and its Complications. 2023;37(2):108389.

15. Crowther NJ, Norris SA. The current waist circumference cut point used for the diagnosis of metabolic syndrome in sub-Saharan African women is not appropriate. PloS one. 2012;7(11):e48883.

16. Fang H, Berg E, Cheng X, Shen W. How to best assess abdominal obesity. Current opinion in clinical nutrition and metabolic care. 2018;21(5):360.

17. Ross R, Neeland IJ, Yamashita S, Shai I, Seidell J, Magni P, et al. Waist circumference as a vital sign in clinical practice: a Consensus Statement from the IAS and ICCR Working Group on Visceral Obesity. 2020. Report No.: 1759-5029 Contract No.: 3.

18. Misra A, Wasir JS, Vikram NK. Waist circumference criteria for the diagnosis of abdominal obesity are not applicable uniformly to all populations and ethnic groups. Nutrition. 2005;21(9):969-76.

19. Nicolaidis S. Environment and obesity. Metabolism. 2019;100:153942.

20. WHO. Waist circumference and waist-hip ratio: report of a WHO expert consultation, Geneva, 8-11 December 2008. Geneva; 2011.

21. Bener A, Yousafzai MT, Darwish S, Al-Hamaq AO, Nasralla EA, Abdul-Ghani M. Obesity index that better predict metabolic syndrome: body mass index, waist circumference, waist hip ratio, or waist height ratio. Journal of obesity. 2013;2013.

22. Gharipour M, Sarrafzadegan N, Sadeghi M, Andalib E, Talaie M, Shafie D, et al. Predictors of metabolic syndrome in the Iranian population: waist circumference, body mass index, or waist to hip ratio? Cholesterol. 2013;2013:198384.

23. Beydoun MA, Kuczmarski MTF, Wang Y, Mason MA, Evans MK, Zonderman AB. Receiver-operating characteristics of adiposity for metabolic syndrome: the Healthy Aging in Neighborhoods of Diversity across the Life Span (HANDLS) study. Public health nutrition. 2011;14(01):77-92.

24. Alberti G, Zimmet P, Shaw J, Grundy SM. The IDF consensus worldwide definition of the metabolic syndrome. Brussels: International Diabetes Federation. 2006;23(5):469-80.

25. Consultation WE. Waist circumference and waist-hip ratio. Report of a WHO Expert Consultation Geneva: World Health Organization. 2008;2008:8-11.

26. Grieger JA, Bianco-Miotto T, Grzeskowiak LE, Leemaqz SY, Poston L, McCowan LM, et al. Metabolic syndrome in pregnancy and risk for adverse pregnancy outcomes: A prospective cohort of nulliparous women. PLoS medicine. 2018;15(12):e1002710.

27. dos Prazeres Tavares H, Dos Santos DCDM, Abbade JF, Negrato CA, De Campos PA, Calderon IMP, et al. Prevalence of metabolic syndrome in non-diabetic, pregnant Angolan women according to four diagnostic criteria and its effects on adverse perinatal outcomes. Diabetology & Metabolic Syndrome. 2016;8:1-12.

28. Bo S, Menato G, Gallo M-L, Bardelli C, Lezo A, Signorile A, et al. Mild gestational hyperglycemia, the metabolic syndrome and adverse neonatal outcomes. Acta obstetricia et gynecologica Scandinavica. 2004;83(4):335-40.

29. Clayton GL, Borges MC, Lawlor DA. The impact of reproductive factors on the metabolic profile of females from menarche to menopause. Nature Communications. 2024;15(1):1103.

30. Mazza E, Troiano E, Ferro Y, Lisso F, Tosi M, Turco E, et al. Obesity, Dietary Patterns, and Hormonal Balance Modulation: Gender-Specific Impacts. Nutrients. 2024;16(11):1629.

31. Zheng L, Yang L, Guo Z, Yao N, Zhang S, Pu P. Obesity and its impact on female reproductive health: Unraveling the connections. Frontiers in Endocrinology. 2024;14:1326546.

32. Araújo J, Ramos E. Paediatric obesity and cardiovascular risk factors–A life course approach. Porto biomedical journal. 2017;2(4):102-10.

33. UBOS. Population and Censuses Kampala: UBOS; 2020 [Available from: https://www.ubos.org/explore-statistics/20/.

34. Sullivan K, Dean A. OPENEPI: A Web-based epidemiologic and statistical calculator for Public Health. 2009; 124: 471–474. 2009.

35. Kaduka LU, Kombe Y, Kenya E, Kuria E, Bore JK, Bukania ZN, et al. Prevalence of metabolic syndrome among an urban population in Kenya. Diabetes Care. 2012;35(4):887-93.

36. WHO. WHO STEPS surveillance manual: the WHO STEPwise approach to chronic disease risk factor surveillance. Geneva: World Health Organization; 2005. Report No.: 9241593830.

37. Petermann-Rocha F, Martínez-Sanguinetti MA, Ho FK, Celis-Morales C, Pizarro A. Optimal cut-off points for waist circumference in the definition of metabolic syndrome in Chile. Public Health Nutrition. 2020;23(16):2898-903.

38. Owolabi EO, Ter Goon D, Adeniyi OV, Ajayi AI. Optimal waist circumference cut-off points for predicting metabolic syndrome among low-income black South African adults. BMC research notes. 2018;11:1-5.

39. Nguyen KA, Peer N, de Villiers A, Mukasa B, Matsha TE, Mills EJ, et al. Optimal waist circumference threshold for diagnosing metabolic syndrome in African people living with HIV infection. Plos one. 2017;12(9):e0183029.

40. StataCorp L. StataCorp stata statistical software: Release 14. StataCorp LP: College Station, TX, USA. 14.1 ed2015.

41. Fluss R, Faraggi D, Reiser B. Estimation of the Youden Index and its associated cutoff point. Biometrical Journal: Journal of Mathematical Methods in Biosciences. 2005;47(4):458-72.

42. Andreu A, Casals G, Vinagre I, Flores L. Obesity management in women of reproductive age. Endocrinología, Diabetes y Nutrición (English ed). 2023;70:85-94.

43. Grover S, Brandt J, Reddy U, Ananth C. Chronic hypertension, perinatal mortality and the impact of preterm delivery: a population-based study. BJOG: An International Journal of Obstetrics & Gynaecology. 2022;129(4):572-9.

44. Alberti, Kurt George Matthew Mayer, Zimmet Paul, Shaw. J. Metabolic Syndrome—a new world-wide definition. A consensus statement from the International Diabetes Federation. Diabetic medicine. 2006;23(5):469-80.

45. Magalhaes P, Capingana DP, Mill JG. Prevalence of the metabolic syndrome and determination of optimal cut-off values of waist circumference in university employees from Angola. South African Journal of Diabetes and Vascular Disease. 2015;12(1):35-40.

46. Cheong KC, Ghazali SM, Hock LK, Yusoff AF, Selvarajah S, Haniff J, et al. Optimal waist circumference cut-off values for predicting cardiovascular risk factors in a multi-ethnic Malaysian population. Obesity research & clinical practice. 2014;8(2):e154-e62.

47. Ogawa D, Kahara K, Shigematsu T, Fujii S, Hayakawa N, Okazaki M, et al. Optimal cut-off point of waist circumference for the diagnosis of metabolic syndrome in Japanese subjects. Journal of Diabetes Investigation. 2010;1(3):117-20.

48. Sinaga M, Worku M, Yemane T, Tegene E, Wakayo T, Girma T, et al. Optimal cut-off for obesity and markers of metabolic syndrome for Ethiopian adults. Nutrition journal. 2018;17(1):1-12.

49. Tladi D, Mokgatlhe L, Shaibu S, Nell T, Mitchell R, Mokgothu C, et al. Determination of optimal cut-off values for waist circumferences used for the diagnosis of the metabolic syndrome among Batswana adults (ELS 32). Cardiovascular Journal of Africa. 2020;31(6):314-8.

50. Motala AA, Esterhuizen T, Pirie FJ, Omar MA. The prevalence of metabolic syndrome and determination of the optimal waist circumference cutoff points in a rural South African community. Diabetes care. 2011;34(4):1032-7.

51. Casari S, Di Paola M, Banci E, Diallo S, Scarallo L, Renzo S, et al. Changing dietary habits: the impact of urbanization and rising socio-economic status in families from Burkina Faso in Sub-Saharan Africa. Nutrients. 2022;14(9):1782.

52. Subramoney S, BjöRkeluND C, Guo X, Skoog I, Bosaeus I, Lissner L. Age-related differences in recommended anthropometric cut-off point validity to identify cardiovascular risk factors in ostensibly healthy women. Scandinavian journal of public health. 2014;42(8):827-33.

53. St-Onge M-P, Gallagher D. Body composition changes with aging: the cause or the result of alterations in metabolic rate and macronutrient oxidation? Nutrition. 2010;26(2):152-5.

54. Kohn MA, Newman TB. The walking man approach to interpreting the receiver operating characteristic curve and area under the receiver operating characteristic curve. Journal of Clinical Epidemiology. 2023;162:182-6.

55. Shan G. Improved confidence intervals for the Youden index. PloS one. 2015;10(7):e0127272.

Supporting Information

S2 Fig: ROC curve for 18-49 years old females in Wakiso district, 2023

S3 Fig: ROC curve for 15-17 years old females in Wakiso district, 2023

S4 Fig: ROC curve for 18-24 years old females in Wakiso district, 2023

S5 Fig: ROC curve for 25-34 years old females in Wakiso district, 2023

S6 Fig: ROC curve for 35-44 years old females in Wakiso district, 2023

S7 Fig: ROC curve for 45-49 years old females in Wakiso district, 2023

Click here to access/download;Figure;S7 Fig.tif

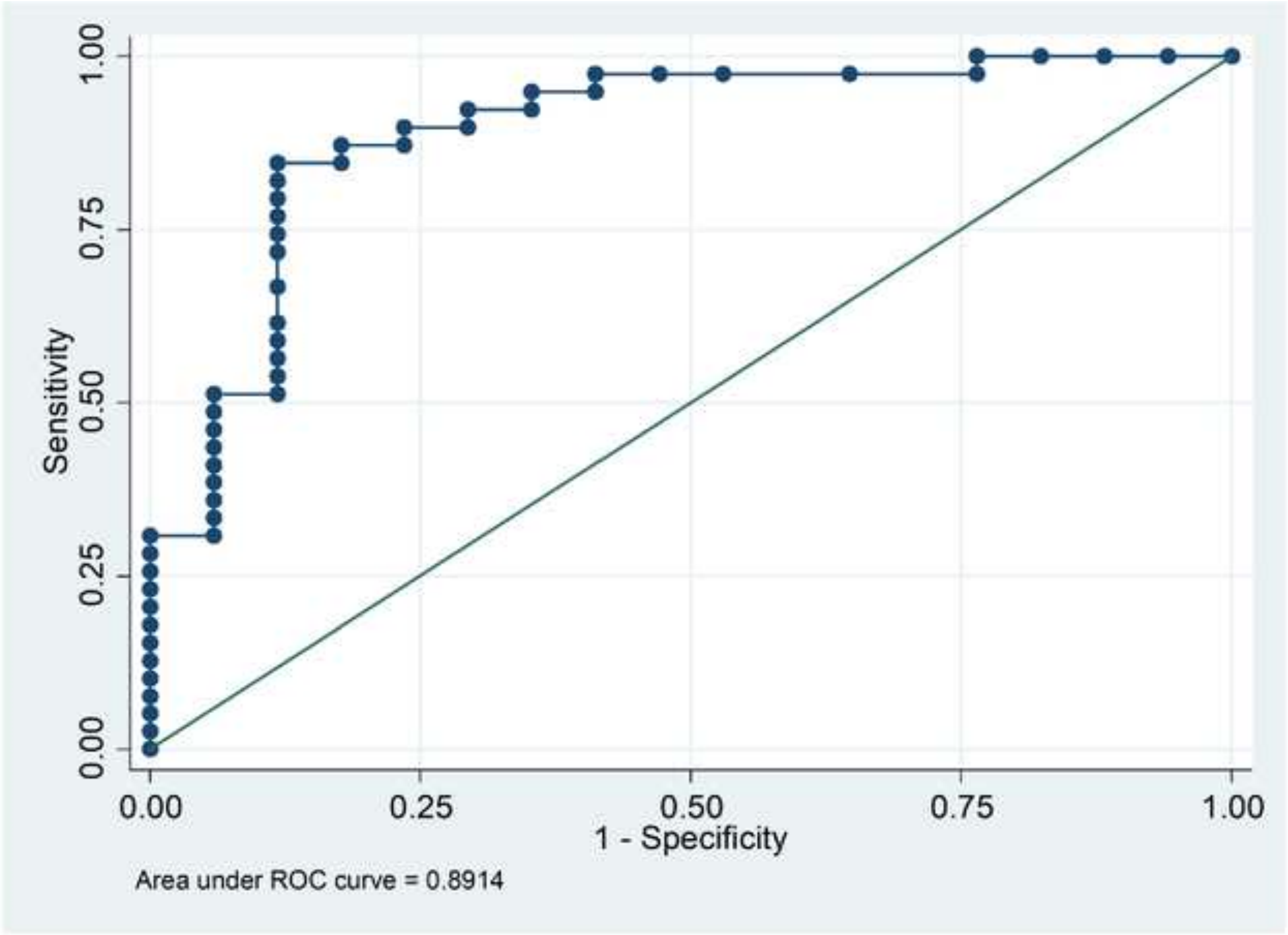

S7 Fig: ROC curve for WC to predict the presence of 2 or more MetS risk factors in females of reproductive age (45-49 years) in Wakiso district, 2023

Click here to access/download;Figure;S2 Fig.tif

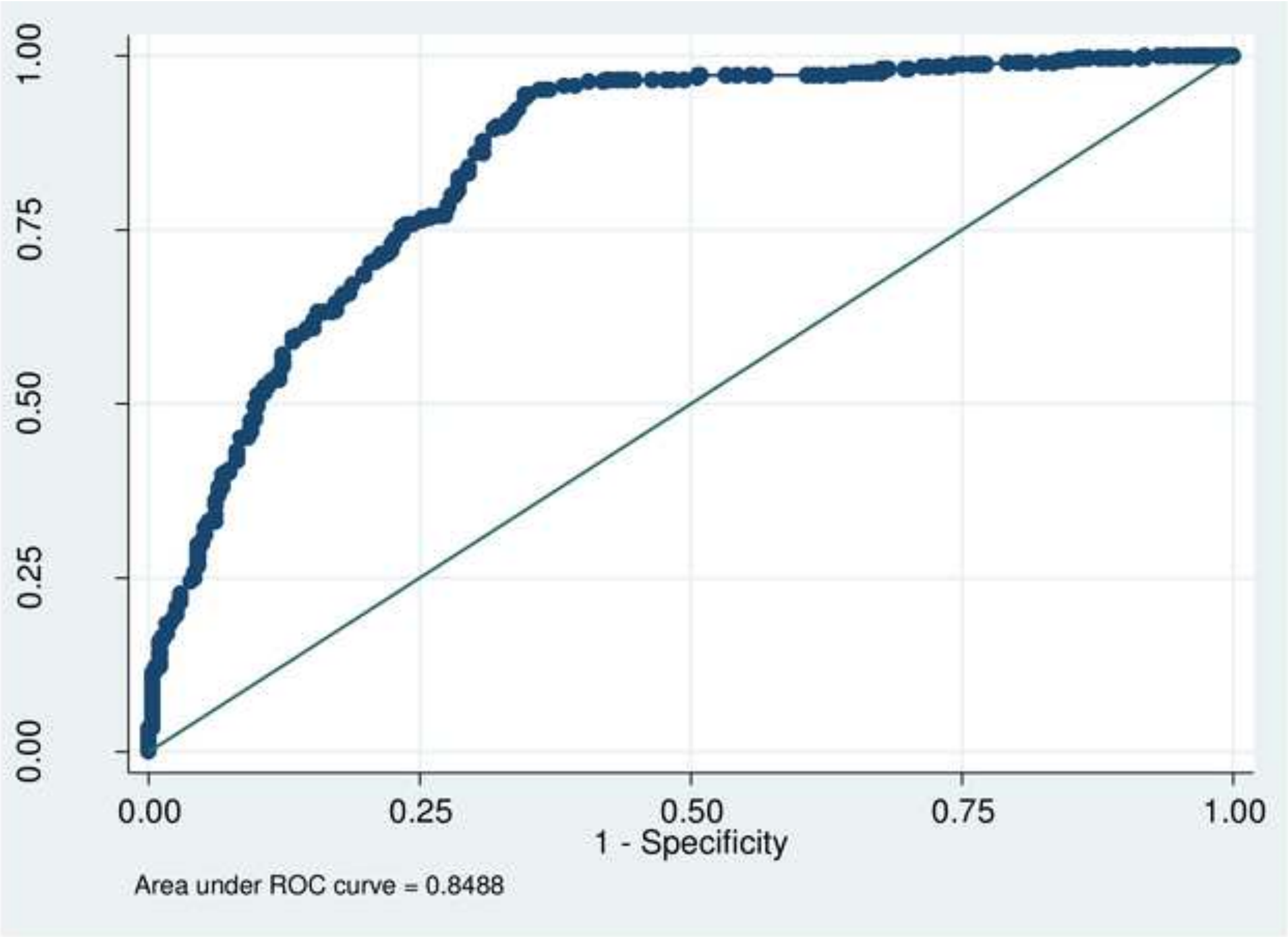

S2 Fig: ROC curve for WC to predict the presence of 2 or more MetS risk factors in females of reproductive age (18-49 years) in Wakiso district, 2023

Click here to access/download;Figure;S4 Fig.tif

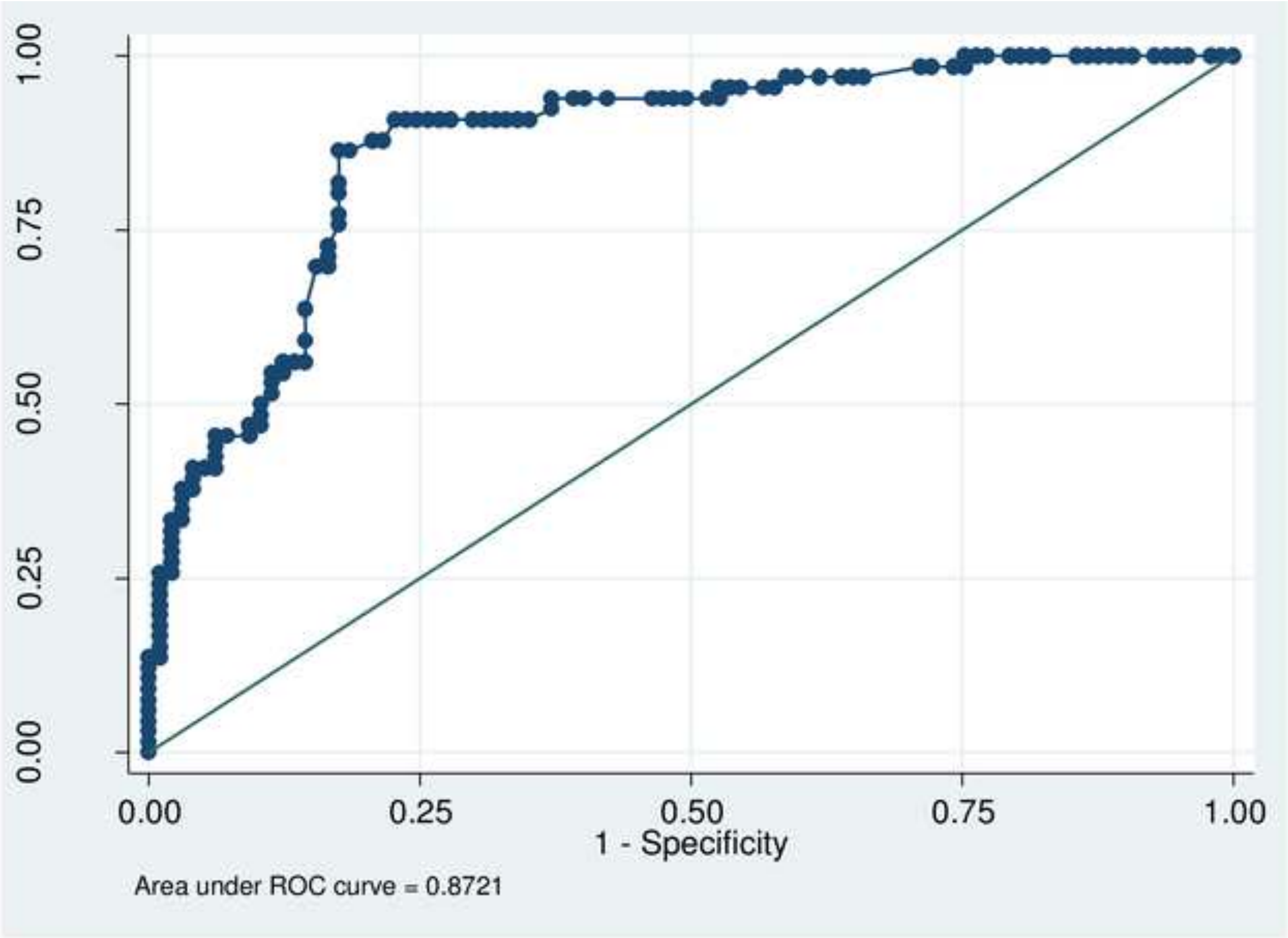

S4 Fig: ROC curve for WC to predict the presence of 2 or more MetS risk factors in females of reproductive age (18-24 years) in Wakiso district, 2023

S1 Fig Click here to access/download;Figure;S1 Fig.tif

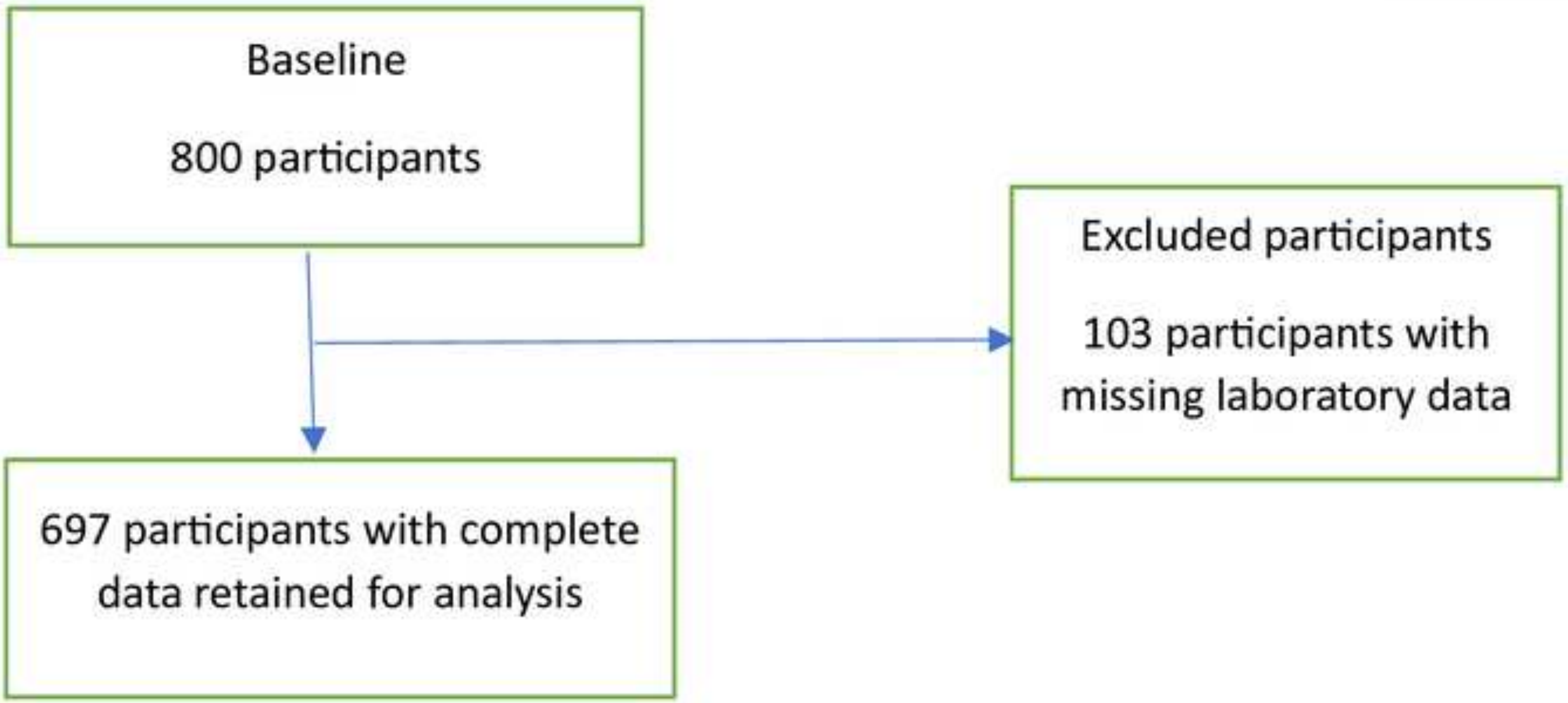

S1 Fig: Flow diagram of study participants

Click here to access/download;Figure;S3 Fig.tif

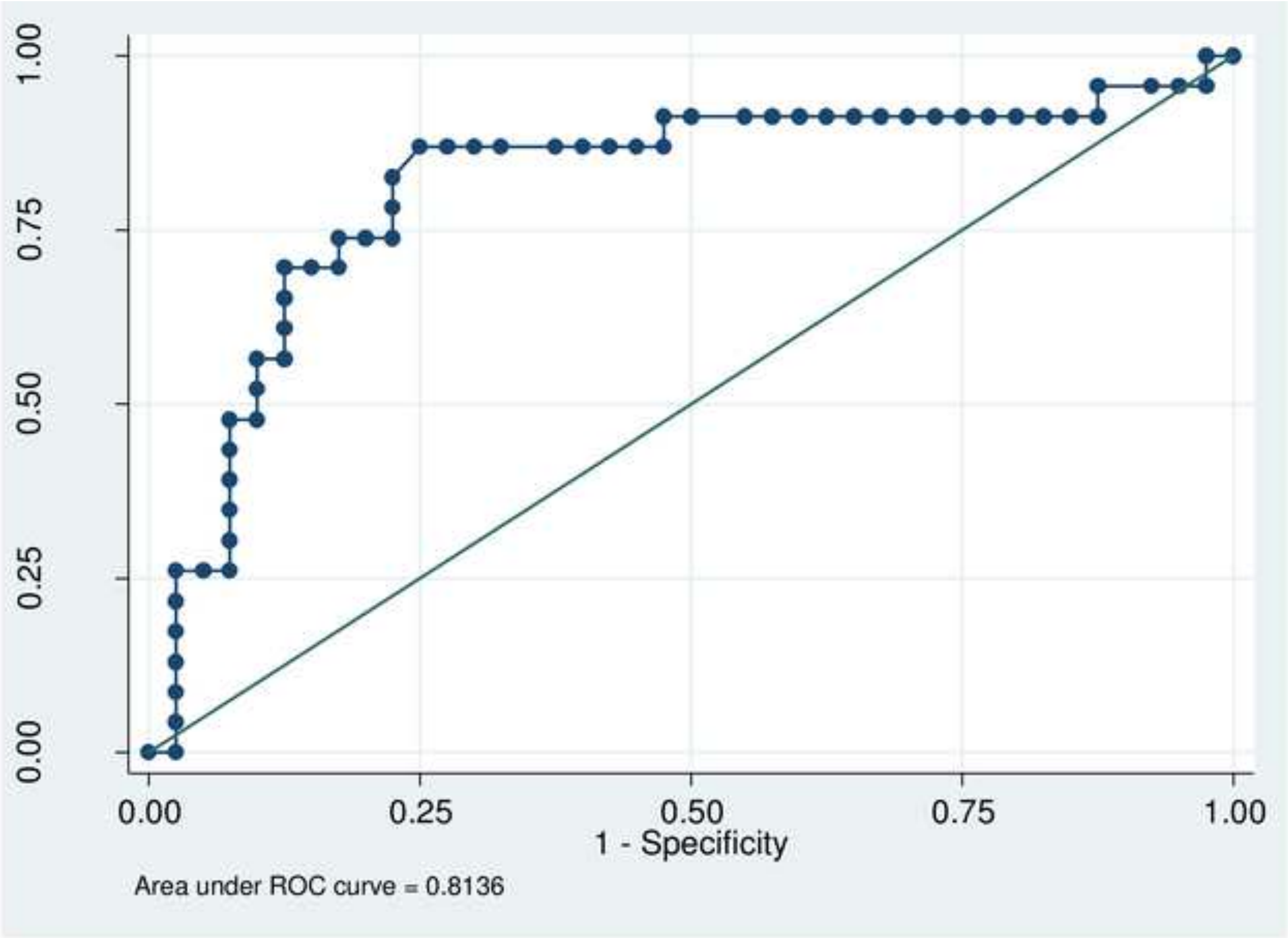

S3 Fig: ROC curve for WC to predict the presence of 2 or more MetS risk factors in females of reproductive age (15-17 years) in Wakiso district, 2023

Click here to access/download;Figure;S5 Fig.tif

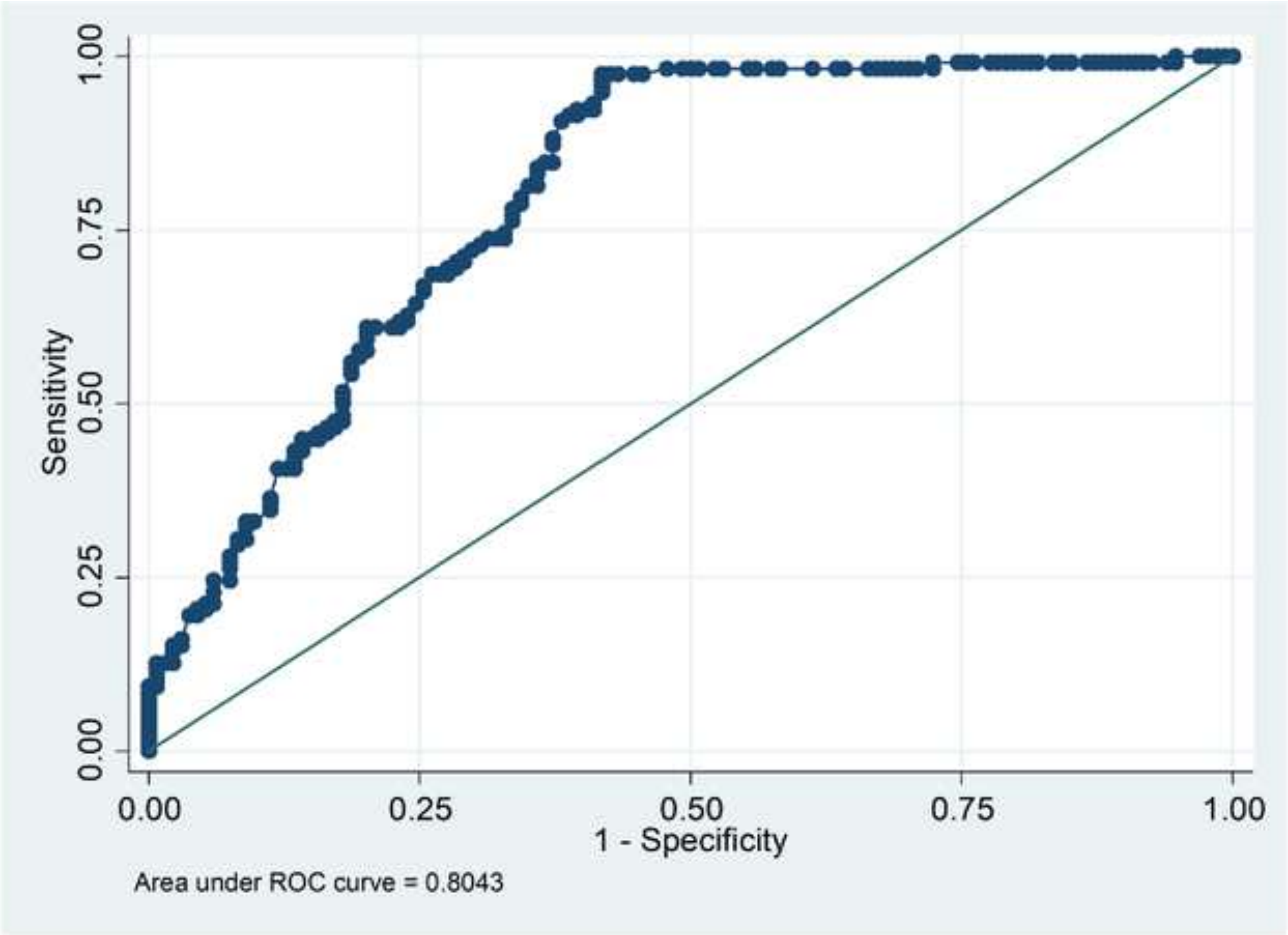

S5 Fig: ROC curve for WC to predict the presence of 2 or more MetS risk factors in females of reproductive age (25-34 years) in Wakiso district, 2023

Click here to access/download;Figure;S6 Fig.tif

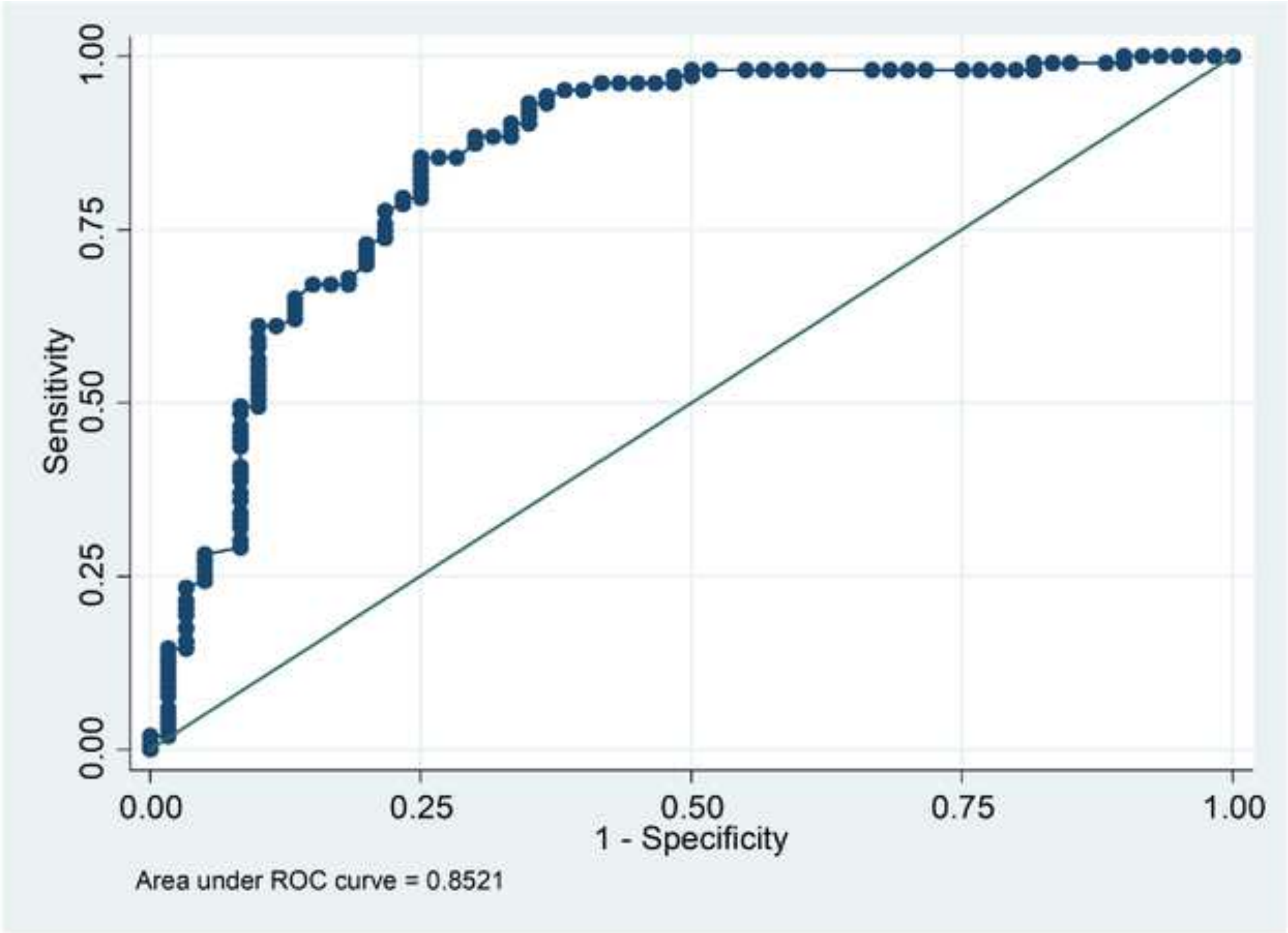

S6 Fig: ROC curve for WC to predict the presence of 2 or more MetS risk factors in females of reproductive age (35-44 years) in Wakiso district, 2023

Data set for optimal WC cut-off points

Click here to access/download

Supporting Information

Data for optimal WC cut off points.xlsx

Optimal waist circumference cut-off points for predicting Metabolic Syndrome among females of reproductive age in Wakiso district, central Uganda

Authors

David Lubogo[1*], Henry Wamani[1], Roy William Mayega[2], Christopher Garimoi Orach[1]

Affiliations

[1]Department of Community Health and Behavioural Sciences, School of Public Health, College of Health Sciences, Makerere University, Kampala, Uganda

[2]Department of Epidemiology and Biostatistics, School of Public Health, College of Health Sciences, Makerere University, Kampala, Uganda

* Corresponding author

Lubogodad@yahoo.com

Abstract

Background

Metabolic Syndrome (MetS) poses a significant challenge to global public health, due to its strong association with Type 2 diabetes and cardiovascular disease. Waist circumference (WC) is a convenient metric for diagnosing MetS. Our study sought to establish waist circumference cut-off points that predict MetS among females of reproductive age in Wakiso district, central Uganda.

Methods:

The data collected were from a cross-sectional study conducted in Wakiso district, central Uganda, involving 697 randomly selected females aged 15 to 49, between 9th June 2021 and 17th August 2021. Data included MetS components: WC, High-Density Lipoprotein (HDL) Cholesterol, triglycerides, blood pressure, and fasting blood sugar. MetS status was identified based on two or more MetS components excluding WC. ROC analysis established the average optimal WC. The accuracy and performance of the cut-off points were evaluated through sensitivity, specificity, Positive likelihood ratio, and the Youden index.

Results:

Of the 697 participants, 49.9% had two or more MetS risk factors. For females aged 18~~5~~-49 years, the average optimal WC cut-off was 80.3 cm. Variations in optimal WC thresholds were observed across different age groups: 78.9 ~~97.4~~ cm for (15-17~~24~~ years), 80.4 cm (18-24 years), 79.9 cm (25-34 years), 85.6 cm (35-44 years), and 91.1 cm (45-49~~54~~ years) respectively. The area under the ROC curve at cut point for these age groups ranged from 0.78 to 0.86, indicating good discriminatory capability. The sensitivity ranged from 85% to 97%, specificity from 58% to 88%, and the Youden Index from 0.557 to 0.729.

Conclusions:

A waist circumference of 80.3 cm was the optimal threshold for identifying Metabolic Syndrome in females between the ages of 15 and 49 years in the setting. This finding concurs with the guidance set forth by the International Diabetes Federation.

Additionally, study participants had varying WC cut-offs ranging from 79.4 cm to 91.1 cm, depending on their age.

Keywords: Cut-off points, females, optimal, reproductive age, waist circumference

Introduction

Metabolic Syndrome (MetS) corresponds to the combination of several disorders closely related to abdominal obesity (1), a strong predictor of adverse metabolic outcomes including insulin resistance, hypertension, and dyslipidemia ~~refers to the accumulation of various risk factors related to cardiac and metabolic health~~ (2, 3).

There are various definitions for MetS. However, according to the International Diabetes Federation (IDF), a diagnosis of MetS requires abdominal (central) obesity, measured by waist circumference, with ethnic-specific thresholds, ≥ 94cm and ≥ 90 cm for European and Asian Men, respectively, and ≥ 80 cm for all ethnic groups in women, and at least two of the following four factors, ~~Among these risk factors is the presence of abdominal obesity,~~ reduced ~~low~~ HDL Cholesterol, elevated triglycerides , high blood pressure, and elevated fasting blood sugar, (4-6).

MetS is a significant global public health challenge, contributing to high morbidity and mortality, and ~~additionally~~, its occurrence is growing in Africa (7-10). The failure to tackle MetS greatly raises the chances of developing Type 2 diabetes and cardiovascular disease (11-13). Thus early identification of cases and management are key in preventing disease progression and the associated long-term health consequences(14).

~~MetS is more prevalent among females than males (8, 11), directly affects their reproductive role, and contributes to adverse perinatal (15, 16) and maternal outcomes(17).~~

The measurement of waist circumference (WC) serves as a simple and practical indicator for assessing abdominal obesity, which is a key component of Metabolic Syndrome (18, 19). The World Health Organization (WHO) and other scientific organizations strongly recommend using waist circumference measurement in screening, diagnosis, and routine clinical care to guide patient management (5, 20). This requires an acceptable waist circumference cut-off for risk stratification. However, establishing universally accepted WC cut-offs has been

Formatted: Font: (Default) Times New Roman

Formatted: Font: (Default) Times New Roman

Formatted: Highlight

Formatted: Highlight

Field Code Changed

Formatted: Highlight

Formatted: Highlight

Field Code Changed

Formatted: Highlight

Formatted: Highlight

Formatted: Highlight

challenging due to differences in abdominal tissue composition among ethnic groups (21). This is because genetics, environmental, and lifestyle elements could impact abdominal obesity (22) and overall body composition (23).

(24)Waist circumference is considered to be a simple, useful and superior (25-27) predictor for MetS. Its simplicity and ability to capture central obesity make it a widely used tool (24) in both clinical and public health practice. Abdominal adipose tissue positively correlates with waist circumference and waist-hip ratio and is associated with several metabolic abnormalities including adverse lipid profiles, decreased insulin sensitivity, and decreased glucose tolerance all of which are risk factors for T2DM and CVD.

The current waist circumference cut-off points for predicting Metabolic Syndrome vary by population and guidelines. For instance, according to the International Diabetes Federation (IDF) guidelines, the waist circumference cut-off for men is≥ 94 cm for Europeans and ≥ 90 cm for South Asians, Chinese and Japanese. For women, the waist circumference is ≥80 cm for all ethnicities (28). The Sub-Saharan Africans use European waist circumference cut-offs(28). On the other hand, the **WHO Expert Consultation (2008)** cut-off points for men are ≥ 94 cm and ≥80 cm for women (29).

Metabolic syndromeS is more prevalent among females than males (8, 11), directly affects their reproductive roles, and contributes to adverse maternal (17) and perinatal (15, 16) outcomes. Women of reproductive age (15-49 years), experience distinct physiological and hormonal changes that influence their health and metabolic health(30). Hormonal fluctuations due to menstrual cycle, pregnancy, and menopause in addition to reproductive health conditions such as polycystic ovary diseases (PCOS), may alter body fat distribution (31) leading to abdominal obesity, a key component of Mets. Abdominal obesity with its associated metabolic abnormalities can directly impact on women's reproductive health (32). Obesity in childhood can also lead to the development of metabolic diseases such as diabetes or cardiovascular disease later in life (33).

MetS is more prevalent among females than males (8, 11), directly affects their reproductive role, and contributes to adverse perinatal (34) and maternal outcomes(17).

Formatted: Font: (Default) Times New Roman, 12 pt

Formatted: Not Highlight

Formatted: Not Highlight

Formatted: Not Highlight

Formatted: Not Highlight

Formatted: Not Highlight

Formatted: Not Highlight

Formatted: Not Highlight

Formatted: Not Highlight

Formatted: Not Highlight

Field Code Changed

Formatted: Not Highlight

Formatted: Not Highlight

Formatted: Not Highlight

Formatted: Not Highlight

Formatted: Not Highlight

In the interim, Uganda and other countries in sub-Saharan Africa use WC cut-offs endorsed by international scientific organizations including the IDF and WHO (4, 23). Nevertheless, it is still unclear whether established thresholds, such as the ≥ 80 cm suggested for females, are valid and appropriate for the African population. Hence, it the necessary to conduct research on the diverse African population groupings including Uganda and to establish sex-specific WC cut-offs as recommended by WHO and others (5, 23).

To date, most studies on optimal waist circumference cut-off points have been conducted in high-income countries. In low-income countries such as Uganda, the optimal ethnic WC cut-off has not been established.particularly for females of reproductive age. Given the unique metabolic challenges faced by women of reproductive age, determining optimal WC cut-off points that are sensitive to their specific physiological context is warranted to improve early detection and management of the condition.

Formatted: Left

Therefore, this study aimed at determining the average waist circumference and age-specific cut-off points that are optimal for predicting MetS among females of reproductive age in Wakiso district, central Uganda. This may assist in the early detection, prevention and management of metabolic syndrome. The hypothesis for the study was that the optimal waist circumference for females aged 15-49 years might not be equal to 80.0cm.

Methods

Formatted: Normal

Study design

We carried out a cross-section study in Wakiso district between 9th June 2021 and 17th August 2021. Our study involved a random sample of female participants from 15 to 49 years of age.

Study population

The study population included females aged 15-49 years residing in Wakiso district, central Uganda. Participants were randomly selected from urban and rural communities to ensure diversity. Eligible participants had resided in Wakiso for at least a year and were identified from a database of 800 individuals, who had anthropometric, physical, and biochemical tests carried out on them to determine the prevalence of MetS in the district.

We excluded participants who were pregnant, those within six weeks after childbirth, alcoholics, smokers, those with chronic diseases such as diabetes mellitus, hypertension, hyperlipidaemia, allergic disorders, depression, and those with known carrier states of hepatitis B, hepatitis C, or human Immunodeficiency virus.

Study setting

The study was conducted in Wakiso district, situated in central Uganda, with an estimated total population of 3,105,700, based on the 2014 census. More than half (52.7%) of the residents are females. Of these, 793,770 are in the reproductive age bracket of 15-49 years (35). The district comprises two counties, namely Kyaddondo and Busiro, and four Municipal Councils - Entebbe, Nansana, Kira, and Makindye-Ssabagabo that together constitute eight sub-counties, eight town councils, 148 parishes, and 704 villages. The Uganda Bureau of Statistics provided the sampling frame for the study and it included 32 randomly selected enumeration areas in the district. Twenty-eight (28) households were sampled per enumeration area.

Sample size calculation

The sample size was computed using the OpenEpi, Version 3.01, open-source calculator for a proportion or descriptive study (36). Using a population size for a finite population of 1,000,000, confidence limits of 5%, a design effect of 2.0., and a prevalence (p) of 40.2% +/-5 obtained from a study conducted among females in an urban population in Kenya to determine MetS prevalence (37).

A sample size of 739 was obtained; after adjusting for a response rate of 90%, the sample size was 813. The final sample used in the study was 800 respondents. We estimated a MetS prevalence of 15% and expected 120 respondents to be with MetS out of the total sample size of 800. This sample was considered appropriate for assessing the sensitivity and specificity of WC as a predictor for MetS in this sample.

Measurements and variables

We used the population sample data set from a prevalence study to obtain participant data on anthropometric measurements (waist circumference), physical measurements (blood pressure), and biochemical measurements (fasting plasma glucose, triglycerides, and low HDL Cholesterol) collected as per the WHO STEP wise approach (38).

Quality control measures were undertaken to ensure the data for the prevalence study were of quality. They included the employment of well-trained data collectors/research assistants (female nurses and graduate nutritionists) who were knowledgeable of the study protocol and had skills in using the Open Data Kit (ODK) for data collection. The data collection tools were field tested from a district near the study district. The data collected in the field were entered in the ODK tool embedded with skip patterns and constraints to ensure data quality and reduce bias. Quantitative data (ODK data) was downloaded from the server and exported to Stata

version 14 for cleaning. This ensured reasonable quality control and data management for this study.

Study variables

Dependent variable:

Metabolic Syndrome is ~~was~~ assessed by determining if an individual has~~d~~ three or more of the five components namely: low HDL cholesterol *(<50 mg/dL or (1.3 mmol/l) in female)* or treatment for it, high triglycerides (≥150 mg/dL (1.7 mmol/L) or treatment for it, elevated fasting blood sugar (≥ 100 mg/dL (5.6 mmol/l) or diabetes diagnosis, or diabetes treatment; high blood pressure (≥130/ ≥85 mmHg) or treatment for it, and increased waist circumference, as defined in the Joint Interim Statement (JIS) (5) .This study assessed MetS using two of the four metabolic components, excluding waist circumference. This approach was used to evaluate the independent contribution of waist circumference to metabolic risk. Similar methods have been applied in previous studies(39-41).

Data analysis

Data were analysed using Stata statistical software (SE/14.0, StatCorp, College Station, TX, USA (42). Descriptive statistics were employed to summarise participant characteristics. Data on continuous variables were summarized using the independent sample T-test and categorical variables were analyzed using the Chi-square test. The results were presented as means with standard deviation and frequencies/percentages, respectively.

Waist circumference cut-off points for various age groups (15-45, 18-45, 15-17, 18-24, 25-34, 35-44, 45-54 years) were evaluated using sensitivity and specificity techniques to identify the most effective predictive values for MetS within the study age groups.

The research investigated the Area under the Curve (AUC) for different components of MetS such as elevated fasting blood sugar, high triglycerides, high blood pressure and low HDL cholesterol. To conduct this analysis, an ROC curve was plotted for the association between waist circumference (WC) and two or more components of MetS.

The optimal cut-off points for WC, which achieves maximum accuracy, were determined by plotting sensitivity and (1-specificity) on a graph.

The study employed the Youden index method (43) expressed as [Maximum (sensitivity + specificity - 1)] to ascertain the optimal WC values and also calculated the positive predictive values (PPV) and negative predictive values (NPV).

Ethical considerations

We obtained approval from the Higher Degrees Research and Ethics Committee of the School of Public Health, College of Health Sciences, Makerere University (Protocal number: MakSPH-REC, 071) and the National Council for Science and Technology of Uganda (Registration number: UNCST, HS1281ES). All study participants provided written informed consent and were assured of the confidentiality of their information. Written informed consent was obtained from parents or guardians of each participant under 18 years of age.

Results

Flow of participants

The figure in S1 Fig shows the flow diagram of the study participants

S1 Fig: Flow diagram of study participants

Characteristics of study participants

Formatted: Left, Space After: 8 pt

~~**Clinical and biochemical characteristics of the study participants**~~

The study involved 697 women of reproductive age with a mean age of 29.7 ± 9.07 years. The majority had completed primary school (38.7%), with 51.5% employed. ~~Participants with incomplete data were not included in the analysis (S6 Fig). On average, they were 29.7 years old (± 9.07), with a mean waist circumference of 86.4 cm (± 13.8) and t~~The mean body mass index (BMI) was~~of~~ 26.9 (±5.8), with 43% in the normal range and 27.6% classified as obese. ~~Almost half of the participants (49.9%) exhibited two or more risk factors linked to MetS (≥ 2 MetS factors)~~, as indicated in Table 1.

Table 1: Characteristics of women of reproductive age in Wakiso district (N=697)

**Characteristics**	**N**	**Percent**
All participants	697	100.0
Age in years (mean, SD)	29.74 (SD=9.07)	
Level of Education attained		
Primary not completed	166	23.8
Primary School	270	38.7
0-Level	154	22.1
A-Level or above	107	15.4
Employment Status		
Paid and employed	359	51.5
Unpaid and Unemployed	338	48.5
BMI[a] (kg/m^2) (Mean, SD)	26.9 (SD=5.8)	
< 18.5	20	2.9
18.5–24.9	300	43.0
25.0–29.9	185	26.5

≥ 30	192	27.6
Fruits and vegetables consumed per day		
< 5 servings	488	70.0
≥ 5 servings	209	30.0
Physical Activity		
Insufficient physical activity	550	78.9
Moderate physical activity	61	8.8
Vigorous physical activity	86	12.3
Current alcohol users (Past 30 days)		
Non-current drinker	605	86.8
Lower end level (< 2 drinks /occasion)	27	3.9
Intermediate end level (2 - < 4 drinks per occasion)	52	7.4
High-end level ≥4 drinks per occasion)	13	1.9
Tobacco Use		
Non-smokers (past 30 days)	689	98.9
Smokers (past 30 days)	8	1.1
Dietary Salt		
Adding salt or salty sauce at meal to food before or when eating		
Sometimes - never	571	82.0
Always or often	126	18.0

~~**Table 1: Clinical and biochemical characteristics of study participants**~~

~~**Variables**~~	~~**Total**~~
	~~**Mean ± SD**~~
~~Number of participants (%)~~	~~697 (100%)~~
~~Age (years)~~	29.7 ± 9.07
~~Multiple risk factor aggregation (MRFA) (≥2 MetS factors)~~	~~49.93%~~
~~Triglyceride (mmol/l)~~	~~1.13 ± 0.62~~
~~HDL cholesterol (mmol/l)~~	~~1.33 ± 0.38~~
~~Fasting blood sugar (mmol/l)~~	~~4.56 ± 1.16~~
~~Body mass index (kg/m2)~~	~~26.9 ± 5.8~~
~~Systolic BP (mmHg)~~	~~117.6 ± 15.3~~
~~Diastolic BP (mmHg)~~	~~76.3 ± 11.2~~
~~Waist circumference (cm)~~	~~86.4 ± 13.8~~

Age-specific evaluation of optimal thresholds for waist circumference using different classification metrics

For the age group 15-45 years, the optimal threshold was 80.25~~3~~ cm. The Youden index, specificity and sensitivity were 0.604, 67% and 94% respectively, and the AUC curve was 0.848 (95% CI 0.819 - 0.877), while for 18-45 years, the optimal threshold was 80.25 cm. The Youden index, specificity and sensitivity were 0.591, 65% and 94% respectively, and the AUC curve was 0.843 (95% CI 0.812 – 0.875).

The optimal WC cut-off points varied among the various age groups, ranging from 78.9 cm for females aged 15 to 17 years, ~~79.4 cm for females aged 15 to 24 years~~ to 91.1 cm for those aged 45 to 49 ~~54~~ years. Across different age groups, the area under the ROC curve at cut point values fluctuated between 0.78 and 0.86, with the 45- 49~~54~~-year-old age group showing the highest AUC of 0.86~~91~~. The 45-49~~54~~ years age group showed the highest discriminative power (AUC =0.891), with specificity (88 %), Youden index (0.729), and a likelihood ratio of (7.08), as shown in Table 2.

Table 2: Age -stratified w~~W~~aist circumference cut-off points~~, and their~~ MetS risk classification metrics, and prevalence of MetS components ~~for MetS risk~~ by age grup.

Variables	Age group						
	**15-17 years(n=63)**	**18~~5~~-24 years (n=163)**	**25-34 years (n=252)**	**35-44 years (n=163)**	**45-49~~54~~ years(n=56)**	**15-49~~5~~ years(n=697)**	**18-49 years(n=634)**
Empirical optimal WC cut-off point (cm)	78.9	80.4 ~~79.4~~	79.9	85.6	91.1	80.3(80.25)	80.25
Area under the ROC curve at the cut point	0.81	0.84 ~~3~~	0.78	0.80	0.86	0.80	0.80
Area under ROC curve (95% CI)	0.814 (0.690 - 0.937)	~~0.8553(0.803 - 0.907)~~ 0.872 (0.817 - 0.927)	0.804(0.750 - 0.858)	0.852(0.786 - 0.918)	0.891 (0.790 - 0.993)	0.848 (0.819 - 0.877)	0.849 (0.819 - 0.879)
Sensitivity at cut point (%)	87	86 ~~90~~	97	85	85	94	94
Specificity at cut point (%)	75	82 ~~77~~	58	75	88	67	65
Youden index (J)	0.620	0.688 ~~65~~	0.557	0.604	0.729	0.604	0.597
LR+ = sensitivity / (1 - specificity)	3.480	4.778 ~~3.913~~	2.3095	3.40	7.08	2.85	2.66
~~Presence of less than 2 metabolic syndrome risk factors (no.)~~		~~137~~	~~134~~	~~60~~	~~17~~	~~348~~	
Presence of 2 or more metabolic syndrome risk factors (%~~no.~~)	36.5	40.5 ~~89~~	46.8 ~~118~~	63.2 ~~103~~	69.6 ~~39~~	50.1 ~~349~~	51.4
~~**Total**~~		~~**226**~~	~~**252**~~	~~**163**~~	~~**56**~~	~~**697**~~	
**Metabolic Syndrome components**							

Elevated waist circumference (%)	35.9	48.0	66.8	77.3	82.8	63	66.1
Elevated triglycerides (%)	10.1	13.1	12.3	15.9	24.4	14.1	14.6
Reduced HDL Cholesterol (%)	44.7	48.9	48.6	47.0	41.2	47.3	47.6
Elevated blood pressure (%)	14.0	21,0	19.5	35.2	42.8	25.0	26.2
Elevated fasting blood sugar (%)	4.1	11.7	5.0	6.8	6.7	7.1	7.4

ROC curve analysis, for waist circumference in reproductive-age women in Wakiso district, for detecting MetS risk factors per age group

Figures in S2-S7~~1 S5~~ Figs show the ROC curves for WC to predict the presence of 2 or more MetS risk factors among females of reproductive age for the various age groups;

S2 Fig: ROC curve for 18~~5~~-49~~5~~ years old females in Wakiso district, 2023

S3 Fig: ROC curve for 15-17 years old females in Wakiso district, 2023

S4~~3~~ Fig: ROC curve for 18~~5~~-24 years old females in Wakiso district, 2023

S5~~4~~ Fig: ROC curve for 25-34 years old females in Wakiso district, 2023

S6~~5~~ Fig: ROC curve for 35-44 years old females in Wakiso district, 2023

S7~~6~~ Fig: ROC curve for 45-49~~54~~ years old females in Wakiso district, 2023

Discussion

The rise in the global prevalence of MetS calls for more research towards early and accurate diagnosis of MetS to provide preventive measures and timely intervention for MetS.

Our study revealed that the mean WC cut-off point of 80.3 cm accurately predicted MetS in females aged 15-49 years in an urban population in Uganda. However, optimal waist circumference varied by age groups (15-17 years), (185-24 years, 25-34 years, 35-44 years, and 45-4954 years) being 78.9 cm, 80.4 cm, 79.4 cm, 79.9 cm, 85.6 cm, and 91.1 cm respectively. This study is the first to use the Joint Interim Statement to establish these cut-off thresholds in Uganda.

The findings showed a strong link between age and the prevalence of several MetS components, particularly elevated waist circumference and blood pressure, both of which increased with age. Reduced HDL cholesterol appeared consistent across all age groups. Public health interventions for preventing metabolic syndrome, should consider targeting abdominal obesity and hypertension, especially for older individuals within the reproductive age range. Research indicates that obesity(44) and hypertension(45) impacts maternal and neonatal health.

Our study has identified an optimal WC threshold value that closely approximates the recommended cut-off of 80.0 cm as specified by the International Diabetes Federation for diagnosing Metabolic Syndrome (MetS)(46). Similar results have been found in prior studies conducted across Africa and other locations. An optimal WC of 80.5 cm was found among female university employees in Angola(47). Elsewhere, some studies among a multiethnic Malaysian population (48) and in a the Japanese population (49) found that 80.0 cm was an optimal WC for women. However, it should be noted that these two studies were conducted among ethnically different populations compared to Africans. The consistency of our findings with those of other researchers strengthens the validity of our findings and reinforces the global relevance of the established diagnostic criteria. From a public health perspective, policymakers

and healthcare professionals may utilize these findings to implement cost-effective and efficient screening programs to enable the early detection and management of MetS.

Our findings, however, differed from a study in Ethiopia, which revealed the optimal WC at 78.0 cm (50). Higher optimal WC cut off points of ≥ 82.3 cm and 92.0 cm were found in studies conducted among women in Botswana and South Africa, respectively(18, 51). These variations highlight the importance of considering region-specific factors when considering WC cutoff points for assessing women's health. The observed variation might be attributed to differences in factors such as genetics, ~~and~~ body composition, and socio-environmental factors across different geographical regions. For instance, countries with higher prevalences of obesity, such as South Africa, often have higher optimal WC cut-off points (18, 52) due to greater fat and different fat distribution. Economic conditions and urbanisation also affect lifestyle and dietary patterns (53), impacting obesity rates and waist circumference measurements.

This study also revealed varying age-specific WC thresholds for various age groups: 78.9 cm for 15-17 years, 80.4~~79.4~~ cm for people aged 18~~5~~-24 years, 79.9 cm for those aged 25-34 years, 85.6 cm for the 35-44 age group, and 91.1 cm for those aged 45-49~~54~~ years respectively. The age-specific cut-off points highlight the variability of MetS risk across various age groups. The younger individuals showed risk factors even with slightly lower waist circumference levels. This emphasizes the importance of early detection and timely intervention in this group. Conversely, elderly individuals exhibited higher waist circumference cut-off points. This is in agreement with other studies (54). Probable reasons for this could be due to changes in body composition with age resulting in reduced muscle mass and again in body fat , and a reduction in metabolic rate (55) with age resulting in weight gain if dietary intake and physical activity remained constant.

Therefore, to effectively combat the rising burden of cardiovascular diseases, healthcare providers and policymakers should include age-specific thresholds for waist circumference in screening and intervention protocols for Metabolic syndrome.

Diagnostic accuracy of the waist circumference cut-off points to detect MetS risk factors

Different metrics were employed to assess the precision of detecting Metabolic Syndrome, including AUC, PPV, specificity and sensitivity, with reference to a specific waist circumference threshold.

When we measured the performance of the AUC values within the 15-17 years and the 18~~5~~-24 age range, the AUC reached 0.81 and 0.84~~3~~, respectively. This data suggests that the WC cut-off point can serve as a reasonably accurate indicator of MetS risk in individuals within this age range. For the 25-34 years age group, the AUC was 0.78, this demonstrates moderate performance. In the 35-44 years age category, the AUC was 0.80, this suggests a relatively good ability to distinguish those at risk for MetS. In the 45-49~~54~~ years age group, the AUC was 0.86., this age group exhibited the highest AUC values, and thus the most robust discriminative power. This suggests that this group may be the most appropriate target for MetS screening using WC. Considering all individuals aged 15-49~~5~~ years or 18-49 years, the overall AUC was 0.80, indicating good performance in identifying MetS. Normally, an AUC of 1 indicates a perfect classifier with the model yielding a 100% true positive rate and 0% false positive rate across all possible threshold values. An AUC of 0 shows that the classifier performs no better than chance, is not able to distinguish between two classes and yields a 0% true positive rate and 100% false positive rate (56). Most classifiers aim for an AUC above 0.5, and that indicates predictive power.

These findings hold important implications for healthcare professionals and researchers, as they provide valuable guidance for developing protocols for screening MetS that are specific to different age groups.

Effectiveness of waist circumference cut-off points in terms of sensitivity and specificity

For the 15-17 age group, a waist circumference of 78.9 cm was associated withwith 87% sensitivity and 75% specificity. The high sensitivity suggests that it is effective in identifying Metabolic Syndrome. However, the moderate specificity indicates some potential for false positives.

Considering sensitivity and specificity values, in the 185-24 age group, a WC of 80.479.4 cm was obtained with 8690% sensitivity and 8277% specificity. The high sensitivity suggests that it is effective in identifying Metabolic Syndrome. However, the moderate specificity indicates some potential for false positives.

The 25-34 age group showed a WC cut-off of 79.9 cm and had the highest sensitivity of 97%. This means that nearly all individuals with Metabolic Syndrome in this age group are accurately identified. However, a lower 58% specificity may imply concerns about potential false positives, and thus there is a need to do confirmation tests.

The 35-44 years age category, had an 85.6 cm cut-off, and balanced sensitivity (85%) and specificity (75%). This implies that it is reasonably practical in identifying those with Metabolic Syndrome and can minimize the risk of false positives, making it a valuable tool for screening.

In the 45-4954 age group, there was a 91.1 cm cut-off point, with an 85% sensitivity and 88% specificity. This implies that this cut-off can accurately identify individuals at risk and can minimise false positives. For the age range of 15-495 years, and 18-49 years, we obtained an 80.25 cm cut-off point, with 94% sensitivity and 67% specificity, and 94% sensitivity and 65%

specificity, respectively. This shows that has the potential to serve as an efficient screening tool. However, the lower specificity of 65% and 67% calls for further evaluation to confirm diagnoses.

Generally, these age-specific WC cut-off points can be used to offer tailored strategies for MetS risk assessment and underscore the importance of age in risk prediction and patient management. Further research may be needed to validate and expand these findings for broader clinical and public health applications.

Youden Index and Positive Likelihood Ratios (LR+)

Our study evaluated age-specific waist circumference (WC) cut-off points and their associated Youden index (J) and Positive Likelihood Ratios (LR+) to determine the diagnostic performance across different age groups.

Our study identified distinctions among the different age categories. The 45-4954-year-old age group showed the highest Youden index (J) of 0.729, indicating a higher overall accuracy than the other age groups. A value of one (1) indicates perfect accuracy, and the closer J is to 1, the better the test's overall performance (57). Furthermore, it showed the highest LR+ value of 7.08. This implies that among individuals within this age group, a positive test result is about 7.08 times more likely to occur in individuals with Metabolic Syndrome compared to those without the syndrome. This makes it a robust diagnostic tool.

The 25-34 years age group showed a slightly lower Youden index (J) of 0.557 (indicating slightly lower overall accuracy) compared to the 185-24 years group with a Youden index (J) of 0.68865. The 25-34 years age group showed an LR+ of 2.3095, demonstrating valuable diagnostic utility. A LR+ of 2.3095 suggests that individuals diagnosed with Metabolic Syndrome have a waist circumference above the cut-off point approximately 2.3 times more often than those without Metabolic Syndrome. Although this LR+ was slightly lower than the

185-24 years group LR+ (Likelihood Ratio Positive) of 4.7883.913, this LR+ is still a valuable indicator of diagnostic strength. The 35-44 years and the broader 15-495 years age range both demonstrated intermediate Youden index (J) values of 0.604, with corresponding LR+ values of 3.4 and 2.85, respectively.

The study demonstrates that for urban Ugandan females aged 185-49 years, 80.3 cm waist circumference is the mean optimal threshold for MetS prediction. However, these findings were based on data from one district and should be interpreted cautiously when generalizing to other populations. Furthermore, waist circumference cut-off points varied depending on age group as indicated; 78.9 cm (15-17 years),80.4 cm79.4 cm (185-24 years), 79.9 cm (25-34 years), 85.6 cm (35-44 years), and 91.1 cm (45-4954 years). Our findings provide useful information for healthcare professionals when considering age-tailored risk assessment strategies for Metabolic Syndrome to enhance the clinical relevance of WC measurements.

Study limitations

We used a cross-sectional study design. MetS is a condition that develops over time, and the measurement of WC in a single time point may not fully capture its dynamic nature. A longitudinal study would be more appropriate for confirming the temporal relationships observed in the study findings.

Our study focused exclusively on females of reproductive age in Wakiso district, central Uganda. Consequently, while the findings are relevant to women in a similar setting, the results may not be generalizable to the entire Ugandan population. Conducting nationally representative studies on optimal waist circumference in the future would help address this limitation and provide a better understanding of MetS in Uganda.

Our study sample was randomly sampled from an existing database. While random sampling ensures diversity, it may miss out on segments of the population not included in the database. This may have led to a potential selection bias.

In computation of the sample size for our study, we used a prevalence of MetS obtained from a study conducted among females in an urban population in Kenya. This may not accurately reflect the current study population characteristics. However, given that our population is female and in an urban setting, we think this limitation was minimised.

Defining MetS based on the presence of 2 or more components while excluding waist circumference, unlike using 3 or more components as in established guidelines can result in overestimation of the prevalence of MetS in the population.

Conclusions and recommendations

We found that a waist circumference of 80.3 cm was the mean optimal threshold for identifying Metabolic Syndrome in females between the ages of 18~~5~~ and 49 years in Wakiso district,

central Uganda. Furthermore, participants exhibited waist circumference cut-offs ranging from 78.979.4 cm to 91.1 cm based on age group. This study highlights the significance and precision of the existing waist circumference guidelines in healthcare. This study calls for the utilization of a waist circumference cut-off threshold to predict Metabolic Syndrome among reproductive-age females in Uganda, based on age group. The findings of this research may be relevant to healthcare providers, policymakers, public health practitioners and researchers. Further research in a wider range of populations encompassing both rural and urban settings is needed to validate these waist circumference cut-off points.

Acknowledgements

We appreciate all the study participants and research assistants who supported the data collection process. We thank all the local leaders and the district health management team members in Wakiso district who facilitated the research teams. We appreciate Dr Arthur Bagonza (PhD) and Dr Freddie Kitutu (PhD) for reviewing the manuscript.

References

1. Paley CA, Johnson MI. Abdominal obesity and metabolic syndrome: exercise as medicine? BMC Sports Science, Medicine and Rehabilitation. 2018;10:1-8.

2. Bovolini A, Garcia J, Andrade MA, Duarte JA. Metabolic syndrome pathophysiology and predisposing factors. International Journal of Sports Medicine. 2021;42(03):199-214.

3. Wang HH, Lee DK, Liu M, Portincasa P, Wang DQ-H. Novel insights into the pathogenesis and management of the metabolic syndrome. Pediatric gastroenterology, hepatology & nutrition. 2020;23(3):189.

4. Alberti KGMM, Zimmet P, Shaw J. Metabolic syndrome—a new world-wide definition. A consensus statement from the international diabetes federation. Diabetic medicine. 2006;23(5):469-80.

5. Alberti K, Eckel RH, Grundy SM, Zimmet PZ, Cleeman JI, Donato KA, et al. Harmonising the Metabolic Syndrome a Joint Interim Statement of the International Diabetes Federation Task Force on Epidemiology and Prevention; National Heart, Lung, and Blood Institute; American Heart Association; World Heart Federation; International Atherosclerosis Society; and International Association for the study of Obesity. Circulation. 2009;120(16):1640-5.

6. Alberti G, Zimmet P, Shaw J, Grundy SM. The IDF consensus worldwide definition of the metabolic syndrome. Brussels: International Diabetes Federation. 2006:1-23.

7. Okafor CI. The metabolic syndrome in Africa: Current trends. Indian journal of endocrinology and metabolism. 2012;16(1):56-66.

8. Bowo-Ngandji A, Kenmoe S, Ebogo-Belobo JT, Kenfack-Momo R, Takuissu GR, Kengne-Nde C, et al. Prevalence of the metabolic syndrome in African populations: A systematic review and meta-analysis. PloS one. 2023;18(7):e0289155.

9. Saklayen MG. The global epidemic of the metabolic syndrome. Current hypertension reports. 2018;20(2):1-8.

Formatted: Line spacing: Double

10. Noubiap JJ, Nansseu JR, Lontchi-Yimagou E, Nkeck JR, Nyaga UF, Ngouo AT, et al. Geographic distribution of metabolic syndrome and its components in the general adult population: A meta-analysis of global data from 28 million individuals. Diabetes research and clinical practice. 2022;188:109924.

11. Faijer-Westerink HJ, Kengne AP, Meeks KA, Agyemang C. Prevalence of metabolic syndrome in sub-Saharan Africa: A systematic review and meta-analysis. Nutrition, Metabolism and Cardiovascular Diseases. 2020;30(4):547-65.

12. Hwang Y-C, Jee J-H, Oh EY, Choi Y-H, Lee M-S, Kim K-W, et al. Metabolic syndrome as a predictor of cardiovascular diseases and type 2 diabetes in Koreans. International journal of cardiology. 2009;134(3):313-21.

13. Mohamed SM, Shalaby MA, El-Shiekh RA, El-Banna HA, Emam SR, Bakr AF. Metabolic syndrome: risk factors, diagnosis, pathogenesis, and management with natural approaches. Food Chemistry Advances. 2023;3:100335.

14. Handelsman Y, Butler J, Bakris GL, DeFronzo RA, Fonarow GC, Green JB, et al. Early intervention and intensive management of patients with diabetes, cardiorenal, and metabolic diseases. Journal of Diabetes and its Complications. 2023;37(2):108389.

15. dos Prazeres Tavares H, Dos Santos DCDM, Abbade JF, Negrato CA, De Campos PA, Calderon IMP, et al. Prevalence of metabolic syndrome in non-diabetic, pregnant Angolan women according to four diagnostic criteria and its effects on adverse perinatal outcomes. Diabetology & Metabolic Syndrome. 2016;8:1-12.

16. Bo S, Menato G, Gallo M-L, Bardelli C, Lezo A, Signorile A, et al. Mild gestational hyperglycemia, the metabolic syndrome and adverse neonatal outcomes. Acta obstetricia et gynecologica Scandinavica. 2004;83(4):335-40.

17. Grieger JA, Bianco-Miotto T, Grzeskowiak LE, Leemaqz SY, Poston L, McCowan LM, et al. Metabolic syndrome in pregnancy and risk for adverse pregnancy outcomes: A prospective cohort of nulliparous women. PLoS medicine. 2018;15(12):e1002710.

18. Crowther NJ, Norris SA. The current waist circumference cut point used for the diagnosis of metabolic syndrome in sub-Saharan African women is not appropriate. PloS one. 2012;7(11):e48883.

19. Fang H, Berg E, Cheng X, Shen W. How to best assess abdominal obesity. Current opinion in clinical nutrition and metabolic care. 2018;21(5):360.

20. Ross R, Neeland IJ, Yamashita S, Shai I, Seidell J, Magni P, et al. Waist circumference as a vital sign in clinical practice: a Consensus Statement from the IAS and ICCR Working Group on Visceral Obesity. 2020. Report No.: 1759-5029 Contract No.: 3.

21. Misra A, Wasir JS, Vikram NK. Waist circumference criteria for the diagnosis of abdominal obesity are not applicable uniformly to all populations and ethnic groups. Nutrition. 2005;21(9):969-76.

22. Nicolaidis S. Environment and obesity. Metabolism. 2019;100:153942.

23. WHO. Waist circumference and waist-hip ratio: report of a WHO expert consultation, Geneva, 8-11 December 2008. Geneva; 2011.

24. Wang H, Liu A, Zhao T, Gong X, Pang T, Zhou Y, et al. Comparison of anthropometric indices for predicting the risk of metabolic syndrome and its components in Chinese adults: a prospective, longitudinal study. BMJ open. 2017;7(9):e016062.

25. Bener A, Yousafzai MT, Darwish S, Al-Hamaq AO, Nasralla EA, Abdul-Ghani M. Obesity index that better predict metabolic syndrome: body mass index, waist circumference, waist hip ratio, or waist height ratio. Journal of obesity. 2013;2013.

26. Gharipour M, Sarrafzadegan N, Sadeghi M, Andalib E, Talaie M, Shafie D, et al. Predictors of metabolic syndrome in the Iranian population: waist circumference, body mass index, or waist to hip ratio? Cholesterol. 2013;2013:198384.

27. Beydoun MA, Kuczmarski MTF, Wang Y, Mason MA, Evans MK, Zonderman AB. Receiver-operating characteristics of adiposity for metabolic syndrome: the Healthy Aging in Neighborhoods of Diversity across the Life Span (HANDLS) study. Public health nutrition. 2011;14(01):77-92.

28. Alberti G, Zimmet P, Shaw J, Grundy SM. The IDF consensus worldwide definition of the metabolic syndrome. Brussels: International Diabetes Federation. 2006;23(5):469-80.

29. Consultation WE. Waist circumference and waist-hip ratio. Report of a WHO Expert Consultation Geneva: World Health Organization. 2008;2008:8-11.

30. Clayton GL, Borges MC, Lawlor DA. The impact of reproductive factors on the metabolic profile of females from menarche to menopause. Nature Communications. 2024;15(1):1103.

31. Mazza E, Troiano E, Ferro Y, Lisso F, Tosi M, Turco E, et al. Obesity, Dietary Patterns, and Hormonal Balance Modulation: Gender-Specific Impacts. Nutrients. 2024;16(11):1629.

32. Zheng L, Yang L, Guo Z, Yao N, Zhang S, Pu P. Obesity and its impact on female reproductive health: Unraveling the connections. Frontiers in Endocrinology. 2024;14:1326546.

33. Araújo J, Ramos E. Paediatric obesity and cardiovascular risk factors–A life course approach. Porto biomedical journal. 2017;2(4):102-10.

34. . !!! INVALID CITATION !!! (15, 16).

35. UBOS. Population and Censuses Kampala: UBOS; 2020 [Available from: https://www.ubos.org/explore-statistics/20/.

36. Sullivan K, Dean A. OPENEPI: A Web-based epidemiologic and statistical calculator for Public Health. 2009; 124: 471–474. 2009.

37. Kaduka LU, Kombe Y, Kenya E, Kuria E, Bore JK, Bukania ZN, et al. Prevalence of metabolic syndrome among an urban population in Kenya. Diabetes Care. 2012;35(4):887-93.

38. WHO. WHO STEPS surveillance manual: the WHO STEPwise approach to chronic disease risk factor surveillance. Geneva: World Health Organization; 2005. Report No.: 9241593830.

39. Petermann-Rocha F, Martínez-Sanguinetti MA, Ho FK, Celis-Morales C, Pizarro A. Optimal cut-off points for waist circumference in the definition of metabolic syndrome in Chile. Public Health Nutrition. 2020;23(16):2898-903.

40. Owolabi EO, Ter Goon D, Adeniyi OV, Ajayi AI. Optimal waist circumference cut-off points for predicting metabolic syndrome among low-income black South African adults. BMC research notes. 2018;11:1-5.

41. Nguyen KA, Peer N, de Villiers A, Mukasa B, Matsha TE, Mills EJ, et al. Optimal waist circumference threshold for diagnosing metabolic syndrome in African people living with HIV infection. Plos one. 2017;12(9):e0183029.

42. StataCorp L. StataCorp stata statistical software: Release 14. StataCorp LP: College Station, TX, USA. 14.1 ed2015.

43. Fluss R, Faraggi D, Reiser B. Estimation of the Youden Index and its associated cutoff point. Biometrical Journal: Journal of Mathematical Methods in Biosciences. 2005;47(4):458-72.

44. Andreu A, Casals G, Vinagre I, Flores L. Obesity management in women of reproductive age. Endocrinología, Diabetes y Nutrición (English ed). 2023;70:85-94.

45. Grover S, Brandt J, Reddy U, Ananth C. Chronic hypertension, perinatal mortality and the impact of preterm delivery: a population-based study. BJOG: An International Journal of Obstetrics & Gynaecology. 2022;129(4):572-9.

46. Alberti, Kurt George Matthew Mayer, Zimmet Paul, Shaw. J. Metabolic Syndrome—a new world-wide definition. A consensus statement from the International Diabetes Federation. Diabetic medicine. 2006;23(5):469-80.

47. Magalhaes P, Capingana DP, Mill JG. Prevalence of the metabolic syndrome and determination of optimal cut-off values of waist circumference in university employees from Angola. South African Journal of Diabetes and Vascular Disease. 2015;12(1):35-40.

48. Cheong KC, Ghazali SM, Hock LK, Yusoff AF, Selvarajah S, Haniff J, et al. Optimal waist circumference cut-off values for predicting cardiovascular risk factors in a multi-ethnic Malaysian population. Obesity research & clinical practice. 2014;8(2):e154-e62.

49. Ogawa D, Kahara K, Shigematsu T, Fujii S, Hayakawa N, Okazaki M, et al. Optimal cut-off point of waist circumference for the diagnosis of metabolic syndrome in Japanese subjects. Journal of Diabetes Investigation. 2010;1(3):117-20.

50. Sinaga M, Worku M, Yemane T, Tegene E, Wakayo T, Girma T, et al. Optimal cut-off for obesity and markers of metabolic syndrome for Ethiopian adults. Nutrition journal. 2018;17(1):1-12.

51. Tladi D, Mokgatlhe L, Shaibu S, Nell T, Mitchell R, Mokgothu C, et al. Determination of optimal cut-off values for waist circumferences used for the diagnosis of the metabolic syndrome among Batswana adults (ELS 32). Cardiovascular Journal of Africa. 2020;31(6):314-8.

52. Motala AA, Esterhuizen T, Pirie FJ, Omar MA. The prevalence of metabolic syndrome and determination of the optimal waist circumference cutoff points in a rural South African community. Diabetes care. 2011;34(4):1032-7.

53. Casari S, Di Paola M, Banci E, Diallo S, Scarallo L, Renzo S, et al. Changing dietary habits: the impact of urbanization and rising socio-economic status in families from Burkina Faso in Sub-Saharan Africa. Nutrients. 2022;14(9):1782.

54. Subramoney S, BjöRkeluND C, Guo X, Skoog I, Bosaeus I, Lissner L. Age-related differences in recommended anthropometric cut-off point validity to identify cardiovascular risk factors in ostensibly healthy women. Scandinavian journal of public health. 2014;42(8):827-33.

55. St-Onge M-P, Gallagher D. Body composition changes with aging: the cause or the result of alterations in metabolic rate and macronutrient oxidation? Nutrition. 2010;26(2):152-5.

56. Kohn MA, Newman TB. The walking man approach to interpreting the receiver operating characteristic curve and area under the receiver operating characteristic curve. Journal of Clinical Epidemiology. 2023;162:182-6.

57. Shan G. Improved confidence intervals for the Youden index. PloS one. 2015;10(7):e0127272.

Supporting Information

S2~~1~~ Fig: ROC curve for 18~~5~~-49~~5~~ years old females in Wakiso district, 2023

S3 Fig: ROC curve for 15-17 years old females in Wakiso district, 2023

S4~~2~~ Fig: ROC curve for 18~~5~~-24 years old females in Wakiso district, 2023

S5~~3~~ Fig: ROC curve for 25-34 years old females in Wakiso district, 2023

S6~~4~~ Fig: ROC curve for 35-44 years old females in Wakiso district, 2023

S7~~5~~ Fig: ROC curve for 45-49~~54~~ years old females in Wakiso district, 2023

10th October 2024

Dear Editor and Reviewers,

We thank the Editor and the Reviewers for taking the time to assess our manuscript. We appreciate the detailed and insightful comments. A point-by-point response to the reviewer's comments is below

Response to Reviewer's Comments

PGPH-D-24-00473
Optimal waist circumference cut-off points for predicting Metabolic Syndrome among females of reproductive age in Wakiso district, central Uganda
PLOS Global Public Health

Thank you for submitting your manuscript to PLOS Global Public Health. After careful consideration, we feel that it has merit but does not fully meet PLOS Global Public Health's publication criteria as it currently stands. Therefore, we invite you to submit a revised version of the manuscript that addresses the points raised during the review process.

Response: Thank you so much for the opportunity to respond to the reviewer's comments.

Please include the following items when submitting your revised manuscript:

- A rebuttal letter that responds to each point raised by the editor and reviewer(s). You should upload this letter as a separate file labeled 'Response to Reviewers'.
- A marked-up copy of your manuscript that highlights changes made to the original version. You should upload this as a separate file labeled 'Revised Manuscript with Track Changes'.
- An unmarked version of your revised paper without tracked changes. You should upload this as a separate file labeled 'Manuscript'.

Guidelines for resubmitting your figure files are available below the reviewer comments at the end of this letter.

Response: Thank you. The guidelines were noted and considered.

Journal Requirements:

1. Please update your online Competing Interests statement. If you have no competing interests to declare, please state: "The authors have declared that no competing interests exist."

2. We note that your Data Availability Statement is currently as follows: "Data already provided as part of the submitted article."

Please confirm at this time whether or not your submission contains all raw data required to replicate the results of your study. Authors must share the "minimal data set" for their submission. PLOS defines the minimal data set to consist of the data required to replicate all study findings reported in the article, as well as related metadata and methods (https://journals.plos.org/plosone/s/data-availability#loc-minimal-data-set-definition).

For example, authors should submit the following data:

- The values behind the means, standard deviations and other measures reported;
- The values used to build graphs;
- The points extracted from images for analysis.

Authors do not need to submit their entire data set if only a portion of the data was used in the reported study.

> ***Response: Thank you. We have provided the data as required in the supporting information files.***

If your submission does not contain these data, please either upload them as Supporting Information files or deposit them to a stable, public repository and provide us with the relevant URLs, DOIs, or accession numbers. For a list of recommended repositories, please see https://journals.plos.org/plosone/s/recommended-repositories.

If there are ethical or legal restrictions on sharing a de-identified data set, please explain them in detail (e.g., data contain potentially sensitive information, data are owned by a third-party organization, etc.) and who has imposed them (e.g., an ethics committee). Please also provide contact information for a data access committee, ethics committee, or other institutional body to which data requests may be sent. If data are owned by a third party, please indicate how others may request data access.

Additional Editor Comments (if provided):

The manuscript aims at identifying an optimal waist circumference cut-off points for predicting Metabolic Syndrome in females of reproductive age in a district in central Uganda and makes a case for further larger studies in the country.

The authors are encouraged to conisder the comments from the reviewer.
Additionally while the results are obtained from one district in Uganda, the authors, Therefore temper statements such as these to indicate the appropriate level of certainity "The study demonstrates that for urban Ugandan females aged 15-49 years, 80.3 cm waist circumference is the mean optimal threshold for MetS prediction.

> ***Response: Thank you. We will consider all the reviewer comments. We have also tempered the statements as suggested. Please see page 21.***

Reviewers' comments:

Reviewer's Responses to Questions

Comments to the Author

1. Does this manuscript meet PLOS Global Public Health’s publication criteria? Is the manuscript technically sound, and do the data support the conclusions? The manuscript must describe methodologically and ethically rigorous research with conclusions that are appropriately drawn based on the data presented.

Reviewer #1: Partly

Reviewer #2: Yes

---

2. Has the statistical analysis been performed appropriately and rigorously?

Reviewer #1: No

Reviewer #2: Yes

---

3. Have the authors made all data underlying the findings in their manuscript fully available (please refer to the Data Availability Statement at the start of the manuscript PDF file)?

The PLOS Data policy requires authors to make all data underlying the findings described in their manuscript fully available without restriction, with rare exception. The data should be provided as part of the manuscript or its supporting information, or deposited to a public repository. For example, in addition to summary statistics, the data points behind means, medians and variance measures should be available. If there are restrictions on publicly sharing data—e.g. participant privacy or use of data from a third party—those must be specified.

Reviewer #1: Yes

Reviewer #2: Yes

---

4. Is the manuscript presented in an intelligible fashion and written in standard English?

PLOS Global Public Health does not copyedit accepted manuscripts, so the language in submitted articles must be clear, correct, and unambiguous. Any typographical or grammatical errors should be corrected at revision, so please note any specific errors here.

Reviewer #1: Yes

Reviewer #2: Yes

---

5. Review Comments to the Author

Please use the space provided to explain your answers to the questions above. You may also include additional comments for the author, including concerns about dual publication, research ethics, or publication ethics. (Please upload your review as an attachment if it exceeds 20,000 characters)

Reviewer #1: Review
Reviewer Recommendation and Comments for Manuscript Number PGPH-D-24-00473

Title: Optimal waist circumference cut-off points for predicting Metabolic Syndrome among females of reproductive age in Wakiso district, central Uganda.

Study aimed at determining the average waist circumference and age-specific cut-off points that are optimal for predicting MetS among females of reproductive age in Wakiso district, central Uganda. The hypothesis for the study was that the optimal waist circumference for females aged 15-49 years might not be equal to 80.0cm

My suggestion of definition:
INSERM/ Metabolic syndrome corresponds to the combination of several disorders related to the presence of excess fat inside the abdomen. Affected individuals have a large waist circumference (greater than 94 cm for men and 80 cm for women) and at least two other abnormalities among the

following: hyperglycemia (excess blood sugar), elevated triglycerides, low "good" HDL cholesterol, and high blood pressure.

The International Diabetes Federation (IDF) has called on experts from around the world to provide a new definition of metabolic syndrome. According to the IDF, a person has metabolic syndrome when they have abdominal obesity (waist circumference greater than 94 cm in men and 80 cm in women) and at least two of the following factors:

• Elevated triglycerides: Triglyceride levels equal to or greater than 1.7 mmol/L, equivalent to 150 mg/dL.
• Low HDL cholesterol (the "good" cholesterol): HDL cholesterol levels less than 1.03 mmol/L (40 mg/dL) in men and less than 1.29 mmol/L (50 mg/dL) in women.
• High blood pressure: Blood pressure, also known as arterial pressure, equal to or greater than 130 mmHg systolic and 85 mmHg diastolic.
• Elevated fasting blood glucose: Fasting venous blood glucose equal to or greater than 5.6 mmol/L (100 mg/dL).

> ***Response: Thank you for the suggestion. We incorporated the IDF definition in the first paragraph of the manuscript on page 4 lines 58-63. We also modified the first statement so that it would not appear as if we were repeating the IDF definition. See lines 55-57. Thank you for this insightful comment.***

My remarks:

Introduction:
Problem: need to rework the **structure of the introduction** and the **writing language**. Avoid **subjectivity and too much oral writing**.

> **Response: Thank you for the comment. We have reviewed and reworked the structure of the introduction following your suggestion and improved the writing language.**

Suggestion:
I suggest the following outline:
• Definition and components of MetS
• Importance of early identification and management
• Significance of Waist Circumference (WC)
- WC as a measure of central obesity
- Comparison with Body Mass Index (BMI)
- WC's role in predicting MetS

> ***Response: Thank you for this comprehensive structure. We have considered all the suggestions and incorporated them into our introduction. Please see pages 2 to 3 lines 58 to 80.***

• Current WC Cut-off Points

- Overview of existing guidelines and cut-off points (e.g., IDF recommendations)
- Variability of cut-off points based on population demographics

> ***Response: Thank you for the suggestions. We have considered all the suggestions and incorporated them into our introduction. See page 5 lines 84 to 90.***

• Focus on Females of Reproductive Age
- Definition and characteristics of this demographic
- Impact of hormonal and physiological changes on metabolic health
- Unique metabolic challenges and risks in this group

Response: Thank you for the suggestions. We have considered all the suggestions and incorporated them into our introduction. See pages 5-6, lines 91-116.

• Research Gap
- Lack of specific studies on WC cut-off points for females of reproductive age
- Importance of identifying precise cut-off points for this population

Response: Thank you for the suggestions. We have considered all the suggestions and incorporated them into our introduction. Pages 4-5, lines 100-111.

• Study Objective
- Aim to determine optimal WC cut-off points for predicting MetS in females of reproductive age
- Potential benefits for early detection, prevention, and management of MetS

Response: Thank you for this comprehensive structure. We have considered all the suggestions and incorporated them into our introduction. See page 5, lines 112-116

METHODS:
• Random Sampling from Database: While random sampling from a database ensures diversity, it may still miss out on segments of the population not included in the database, leading to potential selection bias.

Response: Thank you for the comment. We have taken note of the comment and acknowledged it in our limitations. See page 22, lines 394-396.

• Sample Size Calculation: The sample size calculation assumes a response rate and prevalence based on previous studies from different populations, which may not accurately reflect the current study population's characteristics.

Response: Thank you for the comment. We have taken note of the comment and acknowledged it in our limitations. Please see page 22, lines 397-400.

• Analysis: do we have data on sociodemographic information?

•Response: Thank you for this comment. We have updated the manuscript and added information on sociodemographic factors. See pages 11-12, lines 209-216.

RESULTATS:
• Flow-chart missing

Response: Thank you. We have provided the flow chart. Fig S1. Supporting information

• I would suggest to describe each component variables used to identify metabolic syndrome (WC, Elevated triglycerides, Low HDL cholesterol , Blood pressure, Elevated fasting blood glucose)

Response: Thank you. We have indicated the component variables including the cut-off points that are associated with each component variable in page 9, lines 170-174.

• We need the first table describing the study population, provide socio demographic information and also the variables related to the med syndrome.

Response: Thank you for this comment. We have now provided the first table and included the sociodemographic information and variables related to metabolic syndrome including physical activity, diet, alcohol intake, smoking and others. See page 14, Table 2.

• Then a table focusing on the waist circumference cut-off points and their 201 classification metrics for MetS risk

Response: Thank you. We have provided the table on waist circumference and their classification metrics on page 14, Table 2.

• Can we consider survival analysis

Response: Thank you for the comment. We think survival analysis may not be suitable in this case because it requires time-to-event data. Our study was cross-sectional and lacked a time component. We considered using ROC curve analysis to determine waist circumference cut-off points.

Given that your study is cross-sectional and aims to identify optimal waist circumference cut-off points for predicting Metabolic Syndrome (MetS) among females of reproductive age, the recommended modeling analyses would primarily focus on classification and predictive modeling. Here are some suitable methods:

• Logistic Regression Analysis:

- To assess the relationship between waist circumference and the presence of MetS, while controlling for other potential confounders (e.g., age, BMI, lifestyle factors).
- Output: Odds ratios (ORs) indicating the likelihood of MetS associated with different waist circumference values.
- Application: Logistic regression can help determine the optimal cut-off point for waist circumference by evaluating the sensitivity and specificity at various thresholds.

Response: We did not use this method in our study.

• Receiver Operating Characteristic (ROC) Curve Analysis:

- Purpose: To evaluate the diagnostic performance of waist circumference in predicting MetS.
- Output: Area under the curve (AUC) to measure the overall accuracy. The optimal cut-off point can be identified using the Youden Index (sensitivity + specificity - 1).
- Application: ROC analysis will help in identifying the waist circumference value that maximizes both sensitivity and specificity for predicting MetS.

Response: Thank you for this clarification. We appreciate this and we used this method in our study.

• Sensitivity and Specificity Analysis:
- Purpose: To calculate the sensitivity, specificity, positive predictive value (PPV), and negative predictive value (NPV) for various waist circumference cut-off points.
- Application: This analysis will provide detailed information on how well different cut-off points classify individuals as having or not having MetS.

Response: Thank you for this clarification. We used this method in our study.

• Youden Index Calculation:
- Purpose: To find the cut-off point that maximizes the Youden Index, which is a summary measure of the ROC curve.
- Application: The Youden Index helps to identify the threshold that offers the best trade-off between sensitivity and specificity.

Response: Thank you for this clarification. We used this method in our study as well.

Reviewer #2: Dear Author,

Thank you for the opportunity to review your manuscript. After careful consideration, I have several suggestions that may improve the clarity and impact of your work:

I have attached my comments in the Word document; please refer to it for detailed feedback.

Response: Thank you. We have addressed the comments as suggested.

Ethical Considerations: I did not find any issues with dual publication or research ethics.

Response: Thank you for the comment.

Overall Quality: Your manuscript is well-structured and addresses an important topic. With these revisions, it has the potential to contribute significantly to the field.

I look forward to seeing the revised manuscript.

Response: Thank you for the comments. We appreciate.

---

6. PLOS authors have the option to publish the peer review history of their article (what does this mean?). If published, this will include your full peer review and any attached files.

Do you want your identity to be public for this peer review? If you choose “no”, your identity will remain anonymous but your review may still be made public.

For information about this choice, including consent withdrawal, please see our Privacy Policy.

Reviewer #1: No

Reviewer #2: **Yes:** Dr.Lakshmi Priya Nagarajan

While revising your submission, please upload your figure files to the Preflight Analysis and Conversion Engine (PACE) digital diagnostic tool, https://pacev2.apexcovantage.com/. PACE helps ensure that figures meet PLOS requirements.

Response: Thank you. We have used PACE to improve our figures.

We hope that the revised version of our manuscripts meets your expectations.

Kind regards

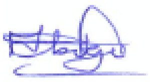

Dr David Lubogo

Corresponding Author.

10th October 2024

Dear Editor and Reviewers,

We thank the Editor and the Reviewers for taking the time to assess our manuscript. We appreciate the detailed and insightful comments. A point-by-point response to the reviewer's comments is below

Response to Reviewer Comments

Abstract Corrections

Page 2, Lines 31-32:

The data collected were from a cross-sectional study conducted in Wakiso district, central Uganda, involving 697 randomly selected females aged 15 to 49, between June 9, 2021, and August 17, 2021.

Q1: Age Range Impact on Metabolic Syndrome

The study includes females aged 15 to 49, covering the reproductive age range. As metabolic risk increases with age, older participants are at higher risk. This broad age range could potentially dilute the overall prevalence of metabolic syndrome (MS) or alter the waist circumference (WC) cut-offs, as the younger age group typically has a lower risk compared to adults. Visceral fat distribution is significantly higher in adults than in teenagers or younger women, which may influence WC measurements and their association with MS. Additionally, hormonal changes and variations in body composition during different life stages, such as puberty and menopause, can affect fat distribution patterns, leading to varying MS risk profiles across age groups.

> ***Response: Thank you. We agree with the reviewer, and we have narrowed the age range to 18-49 years in our calculations so that our results relate to other studies whose findings are comparable to adults. We also provided the 15-49 years for comparison purposes. For instance, page 2, lines 28 and page 3, line 45 and other areas in the manuscript as appropriate.***

Q2: Optimal WC Cut-offs

Page 2, Lines 39-41:

Variations in optimal WC thresholds were observed across different age groups: 79.4 cm for 15-24 years, 79.9 cm for 25-34 years, 85.6 cm for 35-44 years, and 91.1 cm for 45-54 years.

Q2a: It seems there may be a discrepancy; the correct value appears to be 79.4 cm rather than 97.4 cm.

> ***Response: We have corrected the discrepancy. The revised age range is 15-17 years, 18-24 years. 2-34 years, 35 -44 years, and 45 -49 years. We have also recalculated the respective optimal WC thresholds. The revised waist circumferences are 78.9 cm, 80.4 cm, 79.9cm, 85.6 cm and 91.1cm respectively. Page 2, lines 39-40.***

Q2b: Adulthood is generally defined as starting at 18 years of age. International standards set by the IDF, NCEP, and WHO provide waist circumference cut-offs primarily applicable to adults aged 18 years and older and do not include cut-offs for teenagers or adolescents. In this analysis, adolescents and adults aged 15-24 years were grouped together, which may affect the results for this group. Analyzing the 15-17 years age group separately could provide more accurate and relevant assessments.

> ***Response: We agree with the reviewer, and we have reanalysed the data and separated the adolescents (15-17 years) from the adults. The categorisation to 15-17 years yielded an optimal WC cut-off of 78.9 cm, while the optimal WC cut-off of the 15-24 years was 79.4 cm.***
>
> ***Interestingly, the overall optimal WC for 18-49 years did not change much remaining at 80.25 cm, with sensitivity of 94% and specificity of 65%. The overall optimal WC for 15-49 years was 80.25 cm, with a sensitivity of 94% and specificity of 67%. See page 14, table 2.***

Q3: Criteria for Metabolic Syndrome

Page 2, Lines 33-34:
MetS status was identified based on two or more MetS components excluding WC.

Q3a: Could you clarify the criteria used to exclude WC from MetS status? If you referred to any internal criteria, please provide the reference.

> ***Response: Thank you for the question. In this study, we excluded waist circumference from the MetS status criteria so that we could focus on its optimal cut-off points for diagnosing metabolic syndrome in the population under study. Including WC in the initial MetS classification would have introduced bias since we aimed to identify its specific contribution to metabolic risk independently of other established components. This approach allowed us to evaluate the WC diagnostic utility without confounding its role in MetS status. Our decision was guided by the internal study guidelines and literature that has used similar approaches. Please see page 2, lines 32-33 and a clarification on page 9, line 170-178.***

Q3b: According to Alberti et al. (2009), Metabolic Syndrome (MetS) should be classified based on the presence of any three abnormalities, including ethnic-specific waist circumference criteria. However, in your study, only two abnormalities were considered, and WC was excluded. This might lead to potential misclassification if an individual with a normal WC but two other abnormalities is classified as having MetS. Did you assess this potential misclassification by comparing your criteria with international standards?

> ***Response: Thank you for this question regarding the classification of MetS in our study. You are correct that in Alberti et al (2009), MetS is defined by the presence of any three abnormalities, out of the five., while using ethnic-specific WC criteria. However, in our study WC was excluded from the initial Mets criteria to allow for an objective determination of the optimal WC cut-off points specific to our population. The use of two abnormalities was part of our study design to focus on other metabolic components such as blood pressure, fasting blood glucose, triglycerides, and HDL cholesterol, while treating WC as the variable under investigation. This***

decision was taken to avoid circular reasoning, where WC would both define and be analysed in the same context.

We take note of the potential misclassification concern. Defining MetS based on the presence of two or more components, while excluding waist circumference, differs from established guidelines that require three or more components. This may lead to an overestimation of MetS prevalence in the population. We have acknowledged this fact in our study limitations. See page 22, lines 401-403.

"I recommend using the approach by Cook et al. (2003) and Xi et al. (2020), which defines central obesity in adolescents by the 90th percentile of waist circumference (WC). Analyzing adolescent data separately with this method could improve the accuracy of Metabolic Syndrome (MetS) risk assessment and provide valuable age-specific insights, enhancing the precision of your study's findings

Response:

Thank you for the references above. We were able to disaggregate our data and separate the 15-17 years age group from the adults (18 years and above). We analysed the adolescents separately and obtained their age-specific WC cut-off points as earlier indicated above. However, given that we had only one age category of adolescents (15-17 years), with the other four categories (18-24 years, 25-34 years, 35 -44 years and 45-49 years) in the adult category. We could not use the 90th percentile of waist circumference (WC), since most age categories are in the adult category. Thank you.

Q4: Enhancing the Introduction

Revised Text:
To enhance the depth and relevance of the introduction, consider including the following points:

1. "Obesity, with its associated metabolic and endocrine issues, can have a direct impact on women's reproductive health."
2. "Childhood obesity is a significant risk factor for developing metabolic diseases such as cardiovascular disease (CVD) and diabetes in later life."
3. "These conditions can exacerbate reproductive health issues, including polycystic ovary syndrome (PCOS), menstrual disturbances, and infertility."

Response: Thank you for these suggestions. We have incorporated them in our introduction. See page 5. Lines 94 to 99.

Q5: Inconsistency in MetS Criteria

Page 8, Lines 141-142:
Metabolic Syndrome was assessed by determining if an individual had three or more of the five components.

Response: Thank you for pointing out this. We have addressed it on page 9, line 170 to 178.

Page 2, Lines 33-34:
MetS status was identified based on two or more MetS components excluding WC.

Q5: The definitions of MetS in these two sentences differ, indicating an inconsistency. Please review and, if necessary, include a table highlighting the differences.

Response: Thank you for pointing out this. We have addressed it on page 2 lines 32-33 and page 9, lines 170-178.

Figures and Tables

Page 10, Line 181:

Q6. Is this figure S6 or S1?

Response: Thank you. It is figure S1. We have corrected it on page 11, lines 207-208.

Page 10, Lines 185-186: Table 1 indicates that almost half of the participants (49.9%) exhibited two or more risk factors linked to MetS (≥ 2 MetS factors).

Q7. Does this approach include Waist Circumference (WC) in the diagnosis, or was WC excluded?

Response: This approach excludes waist circumference in the diagnosis of MetS. We have explained the reasons for this in our previous responses. Please refer to responses to Q3A and Q3B above.

Table 2: There is a typographical error in the table for the age category 15-45.

Q8. Should this be 15-54?

Response: Thank you for the question. We have corrected this to 18 to 49 following from your previous guidance. See Table 2 on page 14.

Q9. Provide the number (n) for each category in the columns.

Response: Thank you. The numbers are provided. Please refer to page 14 Table 2.

Q10. Include the JIS and IDF criteria along with your current WC cut-off points (if any differences). Additionally, provide the prevalence percentage of each metabolic component overall and within each age category.

Response: Thank you for this question. We agreed to include the IDF criteria definition in the introduction on page 4. However, we thought that our current WC cut-off points used 2 or more components for a definition of MetS, so we think that including the JIS and IDF which use 3 or more MetS components would not be appropriate.

We have provided the overall prevalence percentage of each metabolic syndrome

Country [references]	n	Mean WC	Mean age of the study population	Cut-off point for women (cm
Uganda [24]	6136	77.5(MALE & FEMALE)	>=18 year	≥ 82 to ≥ 85
South Africa [19]	123	93.48±15.68	25–65 years	98
Benin [23]	452	88.6±13.6	25 to 60	94
**Angola [31]**	**615**	**83.9 ± 13.5**	**>20**	**80.5**
South Africa [18]	947	86.0 ± 13.4	>15 years	92
Benin [37]	541	87.6 ± 13.0	25- 60	90
South Africa [21]	152	-	24–65	94
South Africa [38]	1099	-	55–64 y	94
South Africa [22]	920	-	25–74-y	94.6
South Africa (present	998	-	≥ 18	≥ 89.45

component and within each age category on page 14, Table 2.

Optimal WC Cut-offs and Regional Variations in various African studies.

Page 14, Lines 222-226:

Our study has identified an optimal WC threshold value that closely approximates the recommended cut-off of 80.0 cm as specified by the International Diabetes Federation for diagnosing Metabolic Syndrome (MetS) (27). An optimal WC of 80.5 cm was found among female university employees in Angola (28).

Q11a: Except for the Angola study, optimal waist circumference (WC) cut-offs derived from various African regions suggest that the currently used 80 cm cut-off for women may overestimate abdominal obesity. Additionally, many of the studies included adults as volunteers, which may affect the results. It is advisable to recheck the analysis by either removing adolescents or providing a separate analysis for this group or do the age specific cutoff (use 90th percentile).

> ***Response: Thank you for the comment. We appreciate the concern on the overestimation of abdominal obesity using the current 80cm cut-off for women based on findings from various African regions. We re-analysed our data after removing the adolescents (15-17 years). The results of the group analysis to assess optimal WC for adults separately was reported and surprisingly, they did not differ much from the previous findings. See page 14, Table 2.***

Q11b It is my guess that the mean WC of your study population (86.4±13.8), including adolescents. The cut-off may be higher than the present 80.5 cm due to potential misclassification caused by the inclusion of adolescents. Please consider using age-specific categories and redoing the analysis. Kindly check.

> ***Response: Thank you. We confirm that we have reanalysed and checked as suggested. Page 14, Table 2.***

Elsewhere, some studies among a multiethnic Malaysian population (29) and in a Japanese population (30) found that 80.0 cm was an optimal WC for women.

Q12. However, these studies involved ethnically different populations compared to Africans. Comparing your findings with those of your ethnic population and justifying the differences by considering factors such as age, socioeconomic status, lifestyle, dietary patterns, physical activity, and urbanization could provide a more accurate assessment. In the future, conducting a meta-analysis could help derive ethnicity- and age-specific WC cut-offs for Africans.

> ***Response: We agree with the reviewer. We agreed to include a statement that the two cited studies were from an "ethnically different population compared to Africans". The rationale for retaining these studies in our work was to provide a broader perspective, as we have already cited studies from Africa and wanted to incorporate populations from outside the continent for comparison. See page 16. Line 273.***

Page 14, Lines 226-228:

The observed variation might be attributed to differences in factors such as genetics and body composition across different geographical regions. For instance, countries with higher prevalences of obesity, such as South Africa, often have higher optimal WC cut-off points (15, 33).

Q13. The variation in waist circumference (WC) cut-off points for defining central obesity across regions is influenced by genetics, body composition, and socio-environmental factors. For instance, countries with higher obesity rates, like South Africa, often have higher WC cut-offs due to greater body fat and different fat distribution. Economic conditions and urbanization also affect lifestyle and dietary patterns, impacting obesity rates and WC measurements. Including these factors in your discussion can enhance the understanding and interpretation of WC variations in your results.

> ***Response: Thank you for the insightful comments. We have incorporated the suggestions in the discussion. Please see page 17, lines 282-288.***

In Pages 15-18, Lines 249-250:

Probable reasons for the variation could be due to changes in body composition with age, resulting in reduced muscle mass and increased body fat, and a reduction in metabolic rate with age, leading to weight gain if dietary intake and physical activity remain the same. Do

you have data on physical activity and diet? If so, please include this information in your descriptive table. Add relevant references for these sentences.

In Pages 15-18, Lines 254-324:

> ***Response: Thank you for this comment. We have included the necessary reference on page 17, line 298. We have added data on physical activity and diet in the descriptive Table 1 as well.***

Q14. The discussion part repeats the results section, and the discussion points are limited. If your secondary objective is to focus on age and women health, then more information should be included in your manuscript. This will strengthen the discussion.

> ***Response: Thank you for the comment. We have beefed up the discussion points. See page 18, lines 290-295 and 19, lines 315-318 and***

We hope that the revised version of our manuscripts meets your expectations.

Kind regards

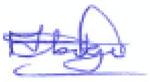

Dr David Lubogo

Corresponding Author.

Click here to access/download
Preprint PDF
Manuscript PLOS Global Public Health David Lubogo.pdf
