# Supplementary material for: Optimal waist circumference cut-off points for predicting metabolic syndrome among females of reproductive age in Wakiso district, central Uganda
Source: PLOS Glob Public Health. 2025 Apr 9;5(4):e0003059. doi: 10.1371/journal.pgph.0003059 (PMC11981207; doi:10.1371/journal.pgph.0003059)
Supplement: Attachment — Submitted filename: Response to Reviewers.pdf [file pgph.0003059.s013.pdf]

20th December 2024

Dear Editor and Reviewers,

We thank the Editor and the Reviewers for taking the time to assess our manuscript. We appreciate the detailed and insightful comments. A point-by-point response to the reviewer's comments is below

PLOS Global Public Health Decision: Revision required [PGPH-D-24-00473R1] - [EMID:a9d1fcfcb0c82d27]

PLOS Global Public Health

CC: sangeetha.shyam@urv.cat, sangeeshyam@gmail.com
PGPH-D-24-00473R1
Optimal waist circumference cut-off points for predicting Metabolic Syndrome among females of reproductive age in Wakiso district, central Uganda
PLOS Global Public Health

Dear Dr. Lubogo,

Thank you for submitting your manuscript to PLOS Global Public Health. After careful consideration, we feel that it has merit but does not fully meet PLOS Global Public Health's publication criteria as it currently stands. Therefore, we invite you to submit a revised version of the manuscript that addresses the points raised during the review process.

Response:
Thank you for your consideration.

Please submit your revised manuscript by Jan 02 2025 11:59PM. If you will need more time than this to complete your revisions, please reply to this message or contact the journal office at globalpubhealth@plos.org. When you're ready to submit your revision, log on to https://www.editorialmanager.com/pgph/ and select the 'Submissions Needing Revision' folder to locate your manuscript file.

Please include the following items when submitting your revised manuscript:

- A rebuttal letter that responds to each point raised by the editor and reviewer(s). You should upload this letter as a separate file labeled 'Response to Reviewers'.
- A marked-up copy of your manuscript that highlights changes made to the original version. You should upload this as a separate file labeled 'Revised Manuscript with Track Changes'.
- An unmarked version of your revised paper without tracked changes. You should upload this as a separate file labeled 'Manuscript'.

Guidelines for resubmitting your figure files are available below the reviewer comments at the end of this letter.

We look forward to receiving your revised manuscript.
Kind regards,
Sangeetha Shyam, M.Sc., PhD
Academic Editor
PLOS Global Public Health
Journal Requirements:

Please review your reference list to ensure that it is complete and correct. If you have cited papers that have been retracted, please include the rationale for doing so in the manuscript text, or remove these references and replace them with relevant current references. Any changes to the reference list should be mentioned in the rebuttal letter that accompanies your revised manuscript. If you need to cite a retracted article, indicate the article's retracted status in the References list and also include a citation and full reference for the retraction notice.

Response:

We reviewed and verified the reference list to ensure its completeness and correctness. We added one reference to the reference list. The reference addresses the reviewer's comment regarding the description of physical activity levels and alcohol use levels (low, intermediate, and high) and how the measurement standards were defined. Please see lines 176 to 177

Additional Editor Comments (if provided):

The authors are commended for addressing the reviewer comments in this version of the manuscript. They are encouraged to address the minor comments from the reviewer

[Note: HTML markup is below. Please do not edit.]

Reviewers' comments:

Reviewer's Responses to Questions

Comments to the Author
1. If the authors have adequately addressed your comments raised in a previous round of review and you feel that this manuscript is now acceptable for publication, you may indicate that here to bypass the "Comments to the Author" section, enter your conflict of interest statement in the "Confidential to Editor" section, and submit your "Accept" recommendation.

Reviewer #2: All comments have been addressed

2. Does this manuscript meet PLOS Global Public Health's publication criteria? Is the manuscript technically sound, and do the data support the conclusions? The manuscript must describe methodologically and ethically rigorous research with conclusions that are appropriately drawn based on the data presented.

Reviewer #2: Yes

3. Has the statistical analysis been performed appropriately and rigorously?

Reviewer #2: Yes

4. Have the authors made all data underlying the findings in their manuscript fully available (please refer to the Data Availability Statement at the start of the manuscript PDF file)?

The PLOS Data policy requires authors to make all data underlying the findings described in their manuscript fully available without restriction, with rare exception. The data should be provided as part of the manuscript or its supporting information, or deposited to a public repository. For example, in addition to summary statistics, the data points behind means, medians and variance measures should be available. If there are restrictions on publicly sharing data—e.g. participant privacy or use of data from a third party—those must be specified.

Reviewer #2: Yes

5. Is the manuscript presented in an intelligible fashion and written in standard English?

PLOS Global Public Health does not copyedit accepted manuscripts, so the language in submitted articles must be clear, correct, and unambiguous. Any typographical or grammatical errors should be corrected at revision, so please note any specific errors here.

Reviewer #2: Yes

6. Review Comments to the Author

Please use the space provided to explain your answers to the questions above. You may also include additional comments for the author, including concerns about dual publication, research ethics, or publication ethics. (Please upload your review as an attachment if it exceeds 20,000 characters)

Reviewer #2: Dear Author
Thank you for the opportunity to review your manuscript on MetS prevalence and WC as a predictor. I commend your work and have a few suggestions for improving clarity.

Response:

Thank you so much for the positive comments.

• BMI Classification: Use standard categories like WHO's cut-offs. However, since adolescents are analyzed in Table 1, the BMI categorization should be done separately for adolescents based on age- and sex-specific percentiles (WHO guidelines) or remove adolescent throughout analysis [because I also saw in table 2 results that reveal that for the 15-17 age group, the AUC value of 0.81 indicates good performance, but the broad confidence interval (0.690-0.937) may be due to the small sample size (n=63). Sensitivity (87%) is high, but specificity (75%) is moderate, and the Youden index (0.620) is relatively low. larger sample sizes in future studies could improve the results.

Response:

Thank you for your valuable comments. We re-analysed the data for adults (18-49 years) after excluding participants aged 15 to 17 years. We used the standard BMI cut-off points as per the WHO recommendations. Please see Table 1 on page 12.

For adolescents (15 to 17 years), as per your recommendation, we used the BMI for age percentiles for girls aged 5-19 years as recommended by WHO. Please see page 12 and 13 for the updated analysis.

We have acknowledged the limitation of the small sample size for the 15-17 years age group and its potential effect on the confidence intervals and performance metrics. We have also suggested that future studies with larger sample sizes among adolescents could enhance the robustness of the findings and improve other performance metrics. Please see lines 406 to 409 on page 22.

• Physical Activity Levels: Specify the classification method, e.g., WHO guidelines, PAL, or GPAQ.

• Alcohol Use Levels: Define low, intermediate, and high levels, and mention the measurement standard used.

Response:

Thank you for the comment. The physical activity levels and alcohol use levels (low, intermediate, and high) and the measurement standards were defined in detail in a previous manuscript currently under peer review. To avoid repetition, we have referred the reader to that manuscript for further information. Please see lines 176 to 177. Thank you.

These comments are also highlighted in the attached draft.

<u>Response:</u>

We have reviewed the comments highlighted in the draft shared and addressed all of them. Thank you

Final Remarks
With these updates, the paper will be clearer and more informative. Thank you for considering these suggestions. I look forward to the impact of your research.

Best regards,

<u>Response:</u>

Thank you so much for the insightful comments. We hope that we have addressed all the comments to the satisfaction of the reviewer and the Editor.

---

7. PLOS authors have the option to publish the peer review history of their article (what does this mean?). If published, this will include your full peer review and any attached files.

Do you want your identity to be public for this peer review? If you choose "no", your identity will remain anonymous but your review may still be made public.

For information about this choice, including consent withdrawal, please see our Privacy Policy.

Reviewer #2: **Yes:** Lakshmi priya Nagarajan

[NOTE: If reviewer comments were submitted as an attachment file, they will be attached to this email and accessible via the submission site. Please log into your account, locate the manuscript record, and check for the action link "View Attachments". If this link does not appear, there are no attachment files.]

While revising your submission, please upload your figure files to the Preflight Analysis and Conversion Engine (PACE) digital diagnostic tool, https://pacev2.apexcovantage.com/. PACE helps ensure that figures meet PLOS requirements. To use PACE, you must first register as a user. Registration is free. Then, login and navigate to the UPLOAD tab, where you will find detailed instructions on how to use the tool. If you encounter any issues or have any questions when using PACE, please email PLOS at figures@plos.org. Please note that Supporting Information files do not need this step.

<u>Response</u>: This was done. Thank you.

In compliance with data protection regulations, you may request that we remove your personal registration details at any time. (Remove my information/details). Please contact the publication office if you have any questions.
