# Supplementary material for: Optimal waist circumference cut-off points for predicting metabolic syndrome among females of reproductive age in Wakiso district, central Uganda
Source: PLOS Glob Public Health. 2025 Apr 9;5(4):e0003059. doi: 10.1371/journal.pgph.0003059 (PMC11981207; doi:10.1371/journal.pgph.0003059)
Supplement: Attachment — Submitted filename: PGPH-D-24-00473_R2_reviewer 12.1.25.pdf [file pgph.0003059.s012.pdf]

PLOS Global Public Health

Optimal waist circumference cut-off points for predicting metabolic syndrome among females of reproductive age in Wakiso district, central Uganda

--Manuscript Draft--

**Manuscript Number:**	PGPH-D-24-00473R2
**Article Type:**	Research Article
**Full Title:**	Optimal waist circumference cut-off points for predicting metabolic syndrome among females of reproductive age in Wakiso district, central Uganda
**Short Title:**	Optimal waist circumference cut-off points for prediction of metabolic syndrome in females
**Corresponding Author:**	David Lubogo, MBChB,MPH<br>Makerere University College of Health Sciences<br>Kampala, UGANDA
**Order of Authors:**	David Lubogo, MBChB,MPH
	Henry Wamani, MBChB, MPhil, PhD
	Roy William Mayega, MBChB, MPH, PhD
	Christopher Garimoi Orach, MBChB, DPH, MMed, MPH, PhD
**Keywords:**	Cut-off points; Females; Optimal; Reproductive age; Waist circumference
**Abstract:**	Background<br>Metabolic syndrome (MetS) poses a significant challenge to global public health, due to its strong association with Type 2 diabetes and cardiovascular disease. Waist circumference (WC) is a convenient metric for diagnosing MetS. Our study sought to establish waist circumference cut-off points that predict MetS among females of reproductive age in Wakiso district, central Uganda.<br>Methods:<br>The data collected were from a cross-sectional study conducted in Wakiso district, central Uganda, involving 697 randomly selected females aged 15 to 49, between 9th June 2021 and 17th August 2021. Data included MetS components: WC, High-Density Lipoprotein (HDL) Cholesterol, triglycerides, blood pressure, and fasting blood sugar. MetS status was identified based on two or more MetS components excluding WC. ROC analysis established the average optimal WC. The accuracy and performance of the cut-off points were evaluated through sensitivity, specificity, Positive likelihood ratio, and the Youden index.<br>Results:<br>Of the 697 participants, 50.1 % had two or more MetS risk factors. The average optimal WC cut-off for females aged 18-49 years was 80.3 cm. Variations in optimal WC thresholds were observed across different age groups: 78.9 cm for (15-17 years), 80.4 cm (18-24 years), 79.9 cm (25-34 years), 85.6 cm (35-44 years), and 91.1 cm (45-49 years) respectively. The area under the ROC curve at the cut point for these age groups ranged from 0.78 to 0.86, indicating good discriminatory capability. The sensitivity ranged from 85% to 97%, specificity from 58% to 88%, and the Youden Index from 0.557 to 0.729.<br>Conclusions:<br>A waist circumference of 80.3 cm was the optimal threshold for identifying metabolic syndrome in females between the ages of 18 and 49 years in the setting. This finding concurs with the guidance set forth by the International Diabetes Federation. Additionally, study participants had varied WC cut-offs ranging from 79.4 cm to 91.1 cm, depending on their age.
**Additional Information:**	
**Question**	**Response**
**Financial Disclosure**	DL received funding for this work from the Government of Uganda through the Makerere University Research and Innovation Fund (MakRIF) and the Strengthening Education and Training Capacity in Sexual and Reproductive Health and Rights (SET-

Powered by Editorial Manager® and ProduXion Manager® from Aries Systems Corporation

Enter a financial disclosure statement that describes the sources of funding for the work included in this submission and the role the funder(s) played. This includes grants and any commercial funding of the work or authors.<br><br>This statement will be typeset if the manuscript is accepted for publication.<br><br>*Please review the submission guidelines and the instructions link below for detailed requirements and guidance.*	SRHR) Project in Uganda. The funders had no role in study design, data collection and analysis, decision to publish, or preparation of the manuscript.
**Competing Interests**<br><br>On behalf of all authors, disclose any competing interests that could be perceived to bias this work.<br><br>This statement will be typeset if the manuscript is accepted for publication.<br><br>*Please review the instructions link below and PLOS Global Public Health's competing interests policy to determine what information must be disclosed at submission.*	The authors have declared that no competing interests exist.
**Data Availability**<br><br>Before publication, Authors are required to make fully available and without restriction all data underlying their findings. Please see our PLOS Data Policy page for detailed information on this policy.<br><br>A **Data Availability Statement**, detailing where the data can be accessed, is required at first submission. Insert your Data Availability Statement in the box below.	Data already provided as part of the submitted article.

Please see the data reporting section of our submission guidelines for instructions on what you need to include in your Data Availability Statement.<br><br>This statement will be typeset if the manuscript is accepted for publication.<br><br>*PLOS allows rare exemptions to address legal and ethical concerns. If you have legal or ethical restrictions, please use the box below to detail these in full sentences for the Journal team to consider.*	

Click here to access/download;Manuscript;Manuscript.docx

Optimal waist circumference cut-off points for predicting metabolic syndrome among females of reproductive age in Wakiso district, central Uganda

Authors

David Lubogo[1*], Henry Wamani[1], Roy William Mayega[2], Christopher Garimoi Orach[1]

Affiliations

[1]Department of Community Health and Behavioural Sciences, School of Public Health, College of Health Sciences, Makerere University, Kampala, Uganda

[2]Department of Epidemiology and Biostatistics, School of Public Health, College of Health Sciences, Makerere University, Kampala, Uganda

* Corresponding author

Lubogodad@yahoo.com

Abstract

Background

Metabolic syndrome (MetS) poses a significant challenge to global public health, due to its strong association with Type 2 diabetes and cardiovascular disease. Waist circumference (WC) is a convenient metric for diagnosing MetS. Our study sought to establish waist circumference cut-off points that predict MetS among females of reproductive age in Wakiso district, central Uganda.

The data collected were from a cross-sectional study conducted in Wakiso district, central Uganda, involving 697 randomly selected females aged 15 to 49, between 9th June 2021 and 17th August 2021. Data included MetS components: WC, High-Density Lipoprotein (HDL) Cholesterol, triglycerides, blood pressure, and fasting blood sugar. MetS status was identified based on two or more MetS components excluding WC. ROC analysis established the average optimal WC. The accuracy and performance of the cut-off points were evaluated through sensitivity, specificity, positive likelihood ratio, and the Youden Index.

Of the 697 participants, 50.1 % had two or more MetS risk factors. The average optimal WC cut-off for females aged 18-49 years was 80.3 cm. Variations in optimal WC thresholds were observed across different age groups: 80.4 cm (18-24 years), 79.9 cm (25-34 years), 85.6 cm (35-44 years), and 91.1 cm (45-49 years) respectively. The area under the ROC curve at the cut point for these age groups ranged from 0.78 to 0.86, indicating good discriminatory capability. The sensitivity ranged from 85% to 97%, specificity from 58% to 88%, and the Youden Index from 0.557 to 0.729.

A waist circumference of 80.3 cm was the optimal threshold for identifying metabolic syndrome in females between the ages of 18 and 49 years in the setting. This finding concurs with the guidance set forth by the International Diabetes Federation.

Additionally, study participants' WC cut-offs varied, ranging from 79.9 cm to 91.1 cm, depending on their age.

Keywords: Cut-off points, Females, Optimal, Reproductive age, Waist circumference

Introduction

Metabolic syndrome (MetS) corresponds to the combination of several disorders closely related to abdominal obesity, (1) which is a strong predictor of adverse metabolic outcomes including insulin resistance, hypertension, and dyslipidemia (2, 3).

There are several definitions for MetS. However, according to the International Diabetes Federation (IDF), a diagnosis of MetS requires the presence of abdominal (central) obesity, measured by waist circumference, with ethnic-specific thresholds of ≥ 94cm and ≥ 90 cm for European and Asian men, respectively, and ≥ 80 cm for women of all ethnic groups, and at least two of the following four factors, reduced HDL Cholesterol, elevated triglycerides, high blood pressure, and elevated fasting blood sugar (4-6).

Metabolic syndrome is a significant global public health challenge, contributing to high morbidity and mortality, with its prevalence increasing in Africa (7-10). The failure to address MetS significantly increases the chances of developing Type 2 diabetes and cardiovascular disease (11-13). Therefore, early identification of cases and management are key in preventing disease progression and the associated long-term health consequences (14).

The measurement of waist circumference (WC) serves as a simple and practical indicator for assessing abdominal obesity, which is a key component of metabolic syndrome (15, 16). The World Health Organization (WHO) and other scientific organizations strongly recommend using waist circumference measurement in screening, diagnosis, and routine clinical care to guide patient management (5, 17). This requires an acceptable waist circumference cut-off for risk stratification. However, establishing universally accepted WC cut-offs has been challenging due to differences in abdominal tissue composition among ethnic groups (18). This is because genetics, environmental, and lifestyle elements impact abdominal obesity (19) and overall body composition (20).

Waist circumference is a simple, useful and superior (21-23) predictor for MetS compared to body mass index. Its simplicity and ability to capture central obesity make it a widely used tool (24) in both clinical and public health practice. Abdominal adipose tissue positively correlates with waist circumference and waist-hip ratio and is associated with several metabolic abnormalities including adverse lipid profiles, decreased insulin sensitivity, and decreased glucose tolerance all of which are risk factors for T2DM and CVD.

The current waist circumference cut-off points for predicting metabolic syndrome vary by population and guidelines. For instance, according to the International Diabetes Federation (IDF) guidelines, the waist circumference cut-off for men is ≥ 94 cm for Europeans and ≥ 90 cm for South Asians, Chinese and Japanese. For women, the waist circumference is ≥80 cm for all ethnicities (24). Sub-Saharan Africans use European waist circumference cut-offs (24). On the other hand, the WHO Expert Consultation (2008) cut-off points for men are ≥ 94 cm and ≥80 cm for women (25).

Metabolic syndrome is more prevalent among females than males (8, 11), directly affects their reproductive roles, and contributes to adverse maternal (26) and perinatal (27, 28) outcomes. Women of reproductive age (15-49 years) experience distinct physiological and hormonal changes that influence their health and metabolic health (29). Hormonal fluctuations due to the menstrual cycle, pregnancy, and menopause, as well as reproductive health conditions such as polycystic ovary diseases (PCOS), can alter body fat distribution (30), leading to abdominal obesity. This abdominal obesity, along with its associated metabolic abnormalities, can directly affect women's reproductive health (31). Obesity in childhood can also lead to the development of metabolic diseases such as diabetes or cardiovascular disease later in life (32).

Uganda and other countries in sub-Saharan Africa use WC cut-offs endorsed by international scientific organizations including the IDF and WHO (4, 20). Nevertheless, it is still unclear whether established thresholds, such as the ≥ 80 cm suggested for females, are valid and

appropriate for the African population. Hence, it the necessary to conduct research on the diverse African population groupings including Uganda and to establish sex-specific WC cut-offs as recommended by WHO and others (5, 20).

To date, most studies on optimal waist circumference cut-off points have been conducted in high-income countries. In low-income countries such as Uganda, the optimal ethnic WC cut-off has not been established particularly for females of reproductive age. Given the unique metabolic challenges faced by women of reproductive age, determining optimal WC cut-off points that are sensitive to their specific physiological context is warranted to improve early detection and management of the condition.

Therefore, this study aimed at determining the average waist circumference and age-specific cut-off points that are optimal for predicting MetS among females of reproductive age in Wakiso district, central Uganda. This may assist in the early detection, prevention and management of metabolic syndrome. The hypothesis for the study was that the optimal waist circumference for females aged 15-49 years might not be equal to 80.0cm.

Methods

Study design

We carried out a cross-section study in Wakiso district between 9th June 2021 and 17th August 2021. Our study involved a random sample of female participants from 15 to 49 years of age.

Study population

The study population included females aged 15-49 years residing in Wakiso district, central Uganda. Participants were randomly selected from urban and rural communities to ensure diversity. Eligible participants had resided in Wakiso for at least a year and were identified from a database of 800 individuals, who had anthropometric, physical, and biochemical tests carried out on them to determine the prevalence of MetS in the district.

We excluded participants who were pregnant, those within six weeks after childbirth, alcoholics, smokers, those with chronic diseases such as diabetes mellitus, hypertension, hyperlipidaemia, allergic disorders, depression, and those with known carrier states of hepatitis B, hepatitis C, or human Immunodeficiency virus.

Study setting

The study was conducted in Wakiso district, situated in central Uganda, with an estimated total population of 3,105,700, based on the 2014 census. More than half (52.7%) of the residents are females. Of these, 793,770 are in the reproductive age bracket of 15-49 years (33). The district comprises two counties, namely Kyaddondo and Busiro, and four Municipal Councils - Entebbe, Nansana, Kira, and Makindye-Ssabagabo that together constitute eight sub-counties, eight town councils, 148 parishes, and 704 villages. The Uganda Bureau of Statistics provided the sampling frame for the study, and it included 32 randomly selected enumeration areas in the district. Twenty-eight (28) households were sampled per enumeration area.

Sample size calculation

The sample size was computed using the OpenEpi, Version 3.01, open-source calculator for a proportion or descriptive study (34). Using a population size for a finite population of 1,000,000, confidence limits of 5%, a design effect of 2.0., and a prevalence (p) of 40.2% +/-5 obtained from a study conducted among females in an urban population in Kenya to determine MetS prevalence (35). A sample size of 739 was obtained; after adjusting for a response rate of 90%, the sample size was 813. The final sample used in the study was 800 respondents. We estimated a MetS prevalence of 15% and expected 120 respondents to be with MetS out of the total sample size of 800. This sample was considered appropriate for assessing the sensitivity and specificity of WC as a predictor for MetS in this sample.

Measurements and variables

We used the population sample data set from a prevalence study to obtain participant data on anthropometric measurements (waist circumference), physical measurements (blood pressure), and biochemical measurements (fasting plasma glucose, triglycerides, and low HDL Cholesterol) collected as per the WHO STEP wise approach (36).

Quality control measures were undertaken to ensure the data for the prevalence study were of quality. They included the employment of well-trained data collectors/research assistants (female nurses and graduate nutritionists) who were knowledgeable of the study protocol and had skills in using the Open Data Kit (ODK) for data collection. The data collection tools were field tested from a district near the study district. The data collected in the field were entered in the ODK tool embedded with skip patterns and constraints to ensure data quality and reduce bias. Quantitative data (ODK data) was downloaded from the server and exported to Stata version 14 for cleaning. This ensured reasonable quality control and data management for this study.

Study variables

Dependent variable:

Metabolic syndrome was assessed by determining if an individual had three or more of the five components namely: low HDL cholesterol (<50 mg/dL or (1.3 mmol/l) in females) or treatment for it, high triglycerides (≥150 mg/dL (1.7 mmol/L) or treatment for it, elevated fasting blood sugar (≥ 100 mg/dL (5.6 mmol/l) or diabetes diagnosis, or diabetes treatment; high blood pressure (≥130/ ≥85 mmHg) or treatment for it, and increased waist circumference. These components were derived from the Joint Interim Statement (JIS) (5). This study assessed MetS using two of the four metabolic components, excluding waist circumference. This approach was used to evaluate the independent contribution of waist circumference to metabolic risk. Similar methods have been applied in previous studies (37-39).

Detailed information on the measurements of the variables shown in the baseline characteristics can be found in Lubogo et al.(2024) (40).

Data analysis

Data were analysed using Stata statistical software (SE/14.0, StatCorp, College Station, TX, USA (41). Descriptive statistics were employed to summarise participant characteristics. Data on continuous variables were summarised using the independent sample T-test and categorical variables were analysed using the Chi-square test. The results were presented as means with standard deviation and frequencies/percentages.

Waist circumference cut-off points for various age groups (15-17, 18-24, 25-34, 35-44, 45-49, 15-49, 18-49, years) were evaluated using sensitivity and specificity techniques to identify the most effective predictive values for MetS within the study age groups.

The research investigated the Area under the Curve (AUC) for different components of MetS such as elevated fasting blood sugar, high triglycerides, high blood pressure and low HDL cholesterol. To conduct this analysis, an ROC curve was plotted for the association between waist circumference (WC) and two or more components of MetS.

The optimal cut-off points for WC, which achieve maximum accuracy, were determined by plotting sensitivity and (1-specificity) on a graph.

The study employed the Youden Index method (42) expressed as [Maximum (sensitivity + specificity - 1)] to ascertain the optimal WC values and also calculated the positive predictive values (PPV) and negative predictive values (NPV).

Ethical considerations

We obtained approval from the Higher Degrees Research and Ethics Committee of the School of Public Health, College of Health Sciences, Makerere University (Protocal number: MakSPH-REC, 071) and the National Council for Science and Technology of Uganda (Registration number: UNCST, HS1281ES). All study participants provided written informed consent and were assured of the confidentiality of their information. Written informed consent was obtained from parents or guardians of participants under 18 years of age.

Results

Flow of participants

The figure in S1 Fig shows the flow diagram of the study participants.

S1 Fig: Flow diagram of study participants

Characteristics of study participants

The study involved 697 women of reproductive age with a mean age of 29.7 ± 9.07 years. Most participants (38.7%) completed primary school, and 51.5% were employed.

For participants aged 18-49 years, the mean body mass index (BMI) was 27.2 (±5.8), 40.1% had a normal BMI, and 28.9% were classified as obese. See Table 1.

Table 1: Characteristics of women of reproductive age in Wakiso district (N=697)

Characteristics	N	Percent
All participants	697	100.0
Age group (n) in years (mean, SD)		
15-17 (n=63)	16.0 ± 0.77	
18-24 (n=163)	21.2 ± 2.06	
25-34 (n=252)	29.0 ± 2.7	
35-44 (n=163)	38.8 ± 2.87	
45-49 (n=56)	46.7 ± 1.70	
15-49 (n=697)	29.7 ±9.07)	
18-49 (n=634)	31.1± 8.36	
Level of Education attained		
Primary not completed	166	23.8
Primary School	270	38.7
0-Level[a]	154	22.1
A-Level[b] or above	107	15.4
Employment Status		
Paid and employed	359	51.5
Unpaid and Unemployed	338	48.5
BMI-for-age percentiles (n=63) *		
< 5 th percentile	0	0
5th-<85th percentile	37	58.8
85th-<95th percentile	13	20.6
≥ 95th percentile	13	20.6
BMI[a] (kg/m^2) (Mean, SD) (n=634)**	27.2 ± 5.8	
< 17.0	0	0
< 18.5	11	1.7
18.5–24.9	254	40.1
25.0–29.9	186	29.3
≥ 30	183	28.9
Fruits and vegetables consumed per day		
< 5 servings	488	70.0
≥ 5 servings	209	30.0
Physical Activity		
Insufficient physical activity	550	78.9
Moderate physical activity	61	8.8
Vigorous physical activity	86	12.3
Current alcohol users (Past 30 days)		
Non-current drinker	605	86.8
Lower end level (< 2 drinks /occasion)	27	3.9
Intermediate end level (2 - < 4 drinks per occasion)	52	7.4
High-end level ≥4 drinks per occasion)	13	1.9
Tobacco Use		
Non-smokers (past 30 days)	689	98.9
Smokers (past 30 days)	8	1.1

Dietary Salt		
Adding salt or salty sauce at meal to food before or when eating		
Sometimes - never	571	82.0
Always or often	126	18.0

**BMI for age percentiles for participants aged 15-17 years old*
***BMI for participants aged 18-49 years old.*
0-Level[a] Ordinary level (lower secondary school)
A-Level[b] Advanced level (Upper secondary school)

Age-specific evaluation of optimal thresholds for waist circumference using different classification metrics and prevalence of MetS components

For the age group 18-49 years, the optimal threshold was 80.3 cm. The Youden Index, specificity and sensitivity were 0.597, 65 % and 94% respectively, and the AUC curve was 0.849 (95% CI 0.819 - 0.879).

The optimal WC cut-off points varied among the various age groups, ranging from 78.9 cm for females aged 15 to 17 years, to 91.1 cm for those aged 45 to 49 years. Across different age groups, the area under the ROC curve at cut-point values fluctuated between 0.78 and 0.86, with the 45- 49-year-old age group showing the highest AUC of 0.86. The 45-49 years age group showed the highest discriminative power (AUC =0.891), with specificity (88 %), Youden Index (0.729), and a likelihood ratio of (7.08).

The prevalence of various MetS components increased with age, particularly elevated waist circumference ($p<0.001$) and blood pressure ($p<0.001$). Reduced HDL cholesterol ($p=0.958$) appeared consistently across all age groups as shown in Table 2.

Table 2: Age-stratified WC cut-off points, MetS risk classification metrics, and prevalence of MetS components by age group.

Variable	Age group						
	**15-17 years(n=63)**	**18-24 years (n=163)**	**25-34 years (n=252)**	**35-44 years (n=163)**	**45-49 years(n=56)**	**15-49 years(n=697)**	**18-49 years(n=634)**
**MetS risk Classification metrics**							
Empirical optimal WC cut-off point (cm)	78.9	80.4	79.9	85.6	91.1	80.3	80.3
Area under the ROC curve at the cut point	0.81	0.84	0.78	0.80	0.86	0.80	0.80
Area under ROC curve (95% CI)	0.814 (0.690 - 0.937)	0.872 (0.817 -0.927)	0.804 (0.750 - 0.858)	0.852 (0.786 - 0.918)	0.891 (0.790 - 0.993)	0.848 (0.819 - 0.877)	0.849 (0.819 - 0.879)
Sensitivity at cut point (%)	87	86	97	85	85	94	94
Specificity at cut point (%)	75	82	58	75	88	67	65
Youden index (J)	0.620	0.688	0.557	0.604	0.729	0.604	0.597
LR+ = sensitivity / (1 - specificity)	3.480	4.778	2.3095	3.40	7.08	2.85	2.66
Presence of 2 or more metabolic syndrome risk factors (%)	36.5	40.5	46.8	63.2	69.6	50.1	51.4
**Metabolic syndrome components**							
Elevated waist circumference (%)[a]	35.9	48.0	66.8	77.3	82.8	63	66.1
Elevated triglycerides (%)[b]	10.1	13.1	12.3	15.9	24.4	14.1	14.6
Reduced HDL Cholesterol (%)[c]	44.7	48.9	48.6	47.0	41.2	47.3	47.6
Elevated blood pressure (%)[d]	14.0	21,0	19.5	35.2	42.8	25.0	26.2
Elevated fasting blood sugar (%)[e]	4.1	11.7	5.0	6.8	6.7	7.1	7.4

[a]($\chi2$=50.94, df=4,p<0.001), [b]($\chi2$=6.62,df=4,p=0.157), [c]($\chi2$=0.65,df=4,p=0.958), [d]($\chi2$=35.88,df=4,p<0.001), [e]($\chi2$=2.91,df=4,p=0.572)
($\chi2$= Pearson chi-square, df=degrees of freedom and p=p.value)

ROC curve analysis, for waist circumference in reproductive-age women in Wakiso district, for detecting MetS risk factors per age group

Figures in S2-S7 Figs show the ROC curves for WC to predict the presence of 2 or more **MetS risk factors among females of reproductive age for the various age groups.**

S2 Fig: ROC curve for 18-49 years old females in Wakiso district, 2023

S3 Fig: ROC curve for 15-17 years old females in Wakiso district, 2023

S4 Fig: ROC curve for 18-24 years old females in Wakiso district, 2023

S5 Fig: ROC curve for 25-34 years old females in Wakiso district, 2023

S6 Fig: ROC curve for 35-44 years old females in Wakiso district, 2023

S7 Fig: ROC curve for 45-49 years old females in Wakiso district, 2023

Discussion

The rise in the global prevalence of MetS calls for more research towards early and accurate diagnosis of MetS to provide preventive measures and timely intervention for MetS.

Our study revealed that the mean waist circumference cut-off points of 80.3 cm accurately predicted MetS in females aged 18-49 years in an urban population in Uganda. However, optimal waist circumference varied by age groups (15-17 years, 18-24 years, 25-34 years, 35-44 years, and 45-49 years) being 78.9 cm, 80.4 cm, 79.9 cm, 85.6 cm, and 91.1 cm respectively. This study is the first to use the Joint Interim Statement to establish these cut-off thresholds in Uganda.

The findings showed a strong link between age and the prevalence of several MetS components, particularly elevated waist circumference ($p<0.001$), and blood pressure ($p<0.001$), both of which increased with age group. Reduced HDL cholesterol ($p=0.958$) appeared consistent across all age groups. Public health interventions for preventing metabolic syndrome should consider targeting abdominal obesity and hypertension, especially for older individuals within the reproductive age range. Research indicates that obesity (43) and hypertension (44) impact maternal and neonatal health.

Our study identified an optimal WC threshold value that closely approximates the recommended cut-off of 80.0 cm as specified by the International Diabetes Federation for diagnosing metabolic syndrome (MetS) (45). Similar results were found in prior studies conducted across Africa and other locations. An optimal WC of 80.5 cm was found among female university employees in Angola (46). Elsewhere, some studies among a multiethnic Malaysian population (47) and in the Japanese population (48) found that 80.0 cm was an optimal WC for women. However, it should be noted that these two studies were conducted among ethnically different populations compared to Africans. The consistency of our findings

with those of other researchers strengthens the validity of our findings and reinforces the global relevance of the established diagnostic criteria. From a public health perspective, policymakers and healthcare professionals can utilize these findings to implement cost-effective and efficient screening programs that facilitate the early detection and management of MetS.

However, our findings differed from a study in Ethiopia, which identified the optimal WC cutoff at 78.0 cm (49). Studies conducted among women in Botswana and South Africa found higher optimal WC cut-off points of ≥ 82.3 cm and 92.0 cm, respectively (15, 50). These variations highlight the importance of considering region-specific factors while establishing WC cutoff points for assessing women's health. The observed variation might be attributed to differences in factors such as genetics, body composition, and socio-environmental factors across different geographical regions. For instance, countries with higher prevalences of obesity, such as South Africa, often have higher optimal WC cut-off points (15, 51) due to greater fat and different fat distribution. Economic conditions and urbanisation also affect lifestyle and dietary patterns (52), impacting obesity rates and waist circumference measurements.

This study also revealed varying age-specific WC thresholds for various age groups: 78.9 cm for 15-17 years, 80.4 cm for people aged 18-24 years, 79.9 cm for those aged 25-34 years, 85.6 cm for the 35-44 years age group, and 91.1 cm for those aged 45-49 years, respectively. The age-specific cut-off points highlight the variability of MetS risk across various age groups. The younger individuals showed risk factors even with slightly lower waist circumference levels. This emphasizes the importance of early detection and timely intervention in this group. Conversely, elderly individuals exhibited higher waist circumference cut-off points. This is in agreement with other studies (53). Probable reasons for this could be due to changes in body composition with age resulting in reduced muscle mass and gain in body fat, and a reduction

in metabolic rate (54) which occurs with age resulting in weight gain if dietary intake and physical activity remained constant.

Therefore, to effectively combat the rising burden of cardiovascular diseases, healthcare providers and policymakers should include age-specific thresholds for waist circumference in screening and intervention protocols for metabolic syndrome.

Diagnostic accuracy of the waist circumference cut-off points to detect MetS risk factors

Different metrics were employed to assess the precision of detecting metabolic syndrome, including AUC, PPV, specificity and sensitivity, with reference to a specific waist circumference threshold.

When we measured the performance of the AUC values within the 15-17 years and the 18-24 age range, the AUC reached 0.81 and 0.84, respectively. This data suggests that the WC cut-off point can serve as a reasonably accurate indicator of MetS risk in individuals within this age range. For the 25-34 years age group, the AUC was 0.78, this demonstrates moderate performance. In the 35-44 years age category, the AUC was 0.80, this suggests a relatively good ability to distinguish those at risk for MetS. In the 45-49 years age group, the AUC was 0.86. This age group exhibited the highest AUC values, and thus the most robust discriminative power. This suggests that this group may be the most appropriate target for MetS screening using WC. Considering all individuals aged 15-49 years or 18-49 years, the overall AUC was 0.80, indicating good performance in identifying MetS. Normally, an AUC of 1 indicates a perfect classifier with the model yielding a 100% true positive rate and 0% false positive rate across all possible threshold values. An AUC of 0 shows that the classifier performs no better than chance, is not able to distinguish between two classes and yields a 0% true positive rate and 100% false positive rate (55). Most classifiers aim for an AUC above 0.5, and that indicates predictive power.

These findings hold important implications for healthcare professionals and researchers, as they provide valuable guidance for developing protocols for screening MetS that are specific to different age groups.

Effectiveness of waist circumference cut-off points in terms of sensitivity and specificity

For the 15-17 age group, a waist circumference of 78.9 cm was associated with 87% sensitivity and 75% specificity. The high sensitivity suggests that it is effective in identifying metabolic syndrome. However, the moderate specificity indicates some potential for false positives in that age group.

Considering sensitivity and specificity values, a WC of 80.4 cm in the 18-24 age group showed 86% sensitivity and 82% specificity. The high sensitivity further suggests its effectiveness in identifying metabolic syndrome.

The 25-34 age group showed a WC cut-off of 79.9 cm and had the highest sensitivity of 97%. This means that nearly all individuals with metabolic syndrome in this age group are accurately identified. However, a lower 58% specificity may imply concerns about potential false positives, and thus there is a need to do confirmation tests.

The 35-44 years age category had an 85.6 cm cut-off, and balanced sensitivity (85%) and specificity (75%). This implies that it is reasonably practical in identifying those with metabolic syndrome and can minimize the risk of false positives, making it a valuable tool for screening.

In the 45-49 age group, there was a 91.1 cm cut-off point, with an 85% sensitivity and 88% specificity. This implies that this cut-off can accurately identify individuals at risk and can minimise false positives. For the age range of 15-49 years, and 18-49 years, we obtained an 80.3 cm cut-off point, with 94% sensitivity and 67% specificity, and 94% sensitivity and 65% specificity, respectively. This shows that it has the potential to serve as an efficient screening

tool. However, the lower specificity of 65% and 67% calls for further evaluation to confirm diagnoses.

Generally, these age-specific WC cut-off points can be used to offer tailored strategies for MetS risk assessment and highlight the importance of age in risk prediction and patient management. Further research may be needed to validate and expand these findings for broader clinical and public health applications.

Youden Index and Positive Likelihood Ratios (LR+)

Our study evaluated age-specific waist circumference (WC) cut-off points and their associated Youden Index (J) and positive likelihood ratios (LR+) to determine the diagnostic performance across different age groups.

Our study identified distinctions among the different age categories. The 45-49-year-old age group showed the highest Youden Index (J) of 0.729, indicating a higher overall accuracy than the other age groups. A value of one (1) indicates perfect accuracy, and the closer J is to 1, the better the test's overall performance (56). Furthermore, it showed the highest LR+ value of 7.08. This implies that among individuals within this age group, a positive test result is about 7.08 times more likely to occur in individuals with metabolic syndrome compared to those without the syndrome. This makes it a robust diagnostic tool.

The 25-34 years age group showed a slightly lower Youden Index (J) of 0.557 (indicating slightly lower overall accuracy) compared to the 18-24 years group with a Youden Index (J) of 0.688. The 25-34 years age group showed an LR+ of 2.3095, demonstrating valuable diagnostic utility. A LR+ of 2.3095 suggests that individuals diagnosed with metabolic syndrome have a waist circumference above the cut-off point approximately 2.3 times more often than those without metabolic syndrome. Although this LR+ was slightly lower than the 18-24 years group LR+ (Likelihood Ratio Positive) of 4.788, this LR+ is still a valuable indicator of diagnostic

strength. The 35-44 years and the broader 15-49 years age range both demonstrated intermediate Youden Index (J) values of 0.604, with corresponding LR+ values of 3.4 and 2.85, respectively.

The study demonstrates that for urban Ugandan females aged 18-49 years, 80.3 cm waist circumference is the mean optimal threshold for MetS prediction. However, these findings were based on data from one district and should be interpreted cautiously when generalizing to other populations. Furthermore, waist circumference cut-off points varied depending on age group as indicated; 78.9 cm (15-17 years), 80.4 cm (18-24 years), 79.9 cm (25-34 years), 85.6 cm (35-44 years), and 91.1 cm (45-49 years). Our findings provide useful information for healthcare professionals when considering age-tailored risk assessment strategies for metabolic syndrome to enhance the clinical relevance of WC measurements.

Study limitations

We used a cross-sectional study design. MetS is a condition that develops over time, and the measurement of WC in a single time point may not fully capture its dynamic nature. A longitudinal study would be more appropriate for confirming the temporal relationships observed in the study findings.

Our study focused exclusively on females of reproductive age in Wakiso district, central Uganda. Consequently, while the findings are relevant to women in a similar setting, the results may not be generalizable to the entire Ugandan population. Conducting nationally representative studies on optimal waist circumference in the future would help address this limitation and provide a better understanding of MetS in Uganda.

Our study sample was randomly sampled from an existing database. While random sampling ensures diversity, it may miss out on segments of the population not included in the database. This may have led to a potential selection bias.

In the computation of the sample size for our study, we used a prevalence of MetS obtained from a study among females in an urban population in Kenya. This may not accurately reflect the current study population characteristics. However, given that our population is female and in an urban setting, we think this limitation was minimised.

The sample size for the 15-17 years age group was relatively small. This may have influenced the confidence interval and performance metrics of waist circumference cut-off points in this age group. Future studies with larger and more representative samples of adolescents may provide more robust findings in this age group.

Defining MetS based on the presence of 2 or more components while excluding waist circumference, unlike using 3 or more components as in established guidelines can result in overestimation of the prevalence of MetS in the population.

Conclusions and recommendations

We found that a waist circumference of 80.3 cm was the optimal mean threshold for identifying metabolic syndrome in females between the ages of 18 and 49 years in Wakiso district, central Uganda. Furthermore, participants exhibited waist circumference cut-offs ranging from 78.9 cm to 91.1 cm based on age group. This study highlights the significance and precision of the existing waist circumference guidelines in healthcare. This study calls for the utilization of a waist circumference cut-off threshold to predict metabolic syndrome among reproductive-age females in Uganda, based on age group. The findings of this research may be relevant to healthcare providers, policymakers, public health practitioners and researchers. Further research in a wider range of populations encompassing both rural and urban settings is needed to validate these waist circumference cut-off points.

Acknowledgements

We appreciate all the study participants and research assistants who supported the data collection process. We thank all the local leaders and the district health management team members in Wakiso district who facilitated the research teams. We appreciate Dr Arthur Bagonza (PhD) and Dr Freddie Kitutu (PhD) for reviewing the manuscript.

References

1. Paley CA, Johnson MI. Abdominal obesity and metabolic syndrome: exercise as medicine? BMC Sports Science, Medicine and Rehabilitation. 2018;10:1-8.

2. Bovolini A, Garcia J, Andrade MA, Duarte JA. Metabolic syndrome pathophysiology and predisposing factors. International Journal of Sports Medicine. 2021;42(03):199-214.

3. Wang HH, Lee DK, Liu M, Portincasa P, Wang DQ-H. Novel insights into the pathogenesis and management of the metabolic syndrome. Pediatric gastroenterology, hepatology & nutrition. 2020;23(3):189.

4. Alberti KGMM, Zimmet P, Shaw J. Metabolic syndrome—a new world-wide definition. A consensus statement from the international diabetes federation. Diabetic medicine. 2006;23(5):469-80.

5. Alberti K, Eckel RH, Grundy SM, Zimmet PZ, Cleeman JI, Donato KA, et al. Harmonising the Metabolic Syndrome a Joint Interim Statement of the International Diabetes Federation Task Force on Epidemiology and Prevention; National Heart, Lung, and Blood Institute; American Heart Association; World Heart Federation; International Atherosclerosis Society; and International Association for the study of Obesity. Circulation. 2009;120(16):1640-5.

6. Alberti G, Zimmet P, Shaw J, Grundy SM. The IDF consensus worldwide definition of the metabolic syndrome. Brussels: International Diabetes Federation. 2006:1-23.

7. Okafor CI. The metabolic syndrome in Africa: Current trends. Indian journal of endocrinology and metabolism. 2012;16(1):56-66.

8. Bowo-Ngandji A, Kenmoe S, Ebogo-Belobo JT, Kenfack-Momo R, Takuissu GR, Kengne-Nde C, et al. Prevalence of the metabolic syndrome in African populations: A systematic review and meta-analysis. PloS one. 2023;18(7):e0289155.

9. Saklayen MG. The global epidemic of the metabolic syndrome. Current hypertension reports. 2018;20(2):1-8.

10. Noubiap JJ, Nansseu JR, Lontchi-Yimagou E, Nkeck JR, Nyaga UF, Ngouo AT, et al. Geographic distribution of metabolic syndrome and its components in the general adult population: A meta-analysis of global data from 28 million individuals. Diabetes research and clinical practice. 2022;188:109924.

11. Faijer-Westerink HJ, Kengne AP, Meeks KA, Agyemang C. Prevalence of metabolic syndrome in sub-Saharan Africa: A systematic review and meta-analysis. Nutrition, Metabolism and Cardiovascular Diseases. 2020;30(4):547-65.

12. Hwang Y-C, Jee J-H, Oh EY, Choi Y-H, Lee M-S, Kim K-W, et al. Metabolic syndrome as a predictor of cardiovascular diseases and type 2 diabetes in Koreans. International journal of cardiology. 2009;134(3):313-21.

13. Mohamed SM, Shalaby MA, El-Shiekh RA, El-Banna HA, Emam SR, Bakr AF. Metabolic syndrome: risk factors, diagnosis, pathogenesis, and management with natural approaches. Food Chemistry Advances. 2023;3:100335.

14. Handelsman Y, Butler J, Bakris GL, DeFronzo RA, Fonarow GC, Green JB, et al. Early intervention and intensive management of patients with diabetes, cardiorenal, and metabolic diseases. Journal of Diabetes and its Complications. 2023;37(2):108389.

15. Crowther NJ, Norris SA. The current waist circumference cut point used for the diagnosis of metabolic syndrome in sub-Saharan African women is not appropriate. PloS one. 2012;7(11):e48883.

16. Fang H, Berg E, Cheng X, Shen W. How to best assess abdominal obesity. Current opinion in clinical nutrition and metabolic care. 2018;21(5):360.

17. Ross R, Neeland IJ, Yamashita S, Shai I, Seidell J, Magni P, et al. Waist circumference as a vital sign in clinical practice: a Consensus Statement from the IAS and ICCR Working Group on Visceral Obesity. 2020. Report No.: 1759-5029 Contract No.: 3.

18. Misra A, Wasir JS, Vikram NK. Waist circumference criteria for the diagnosis of abdominal obesity are not applicable uniformly to all populations and ethnic groups. Nutrition. 2005;21(9):969-76.
19. Nicolaidis S. Environment and obesity. Metabolism. 2019;100:153942.
20. WHO. Waist circumference and waist-hip ratio: report of a WHO expert consultation, Geneva, 8-11 December 2008. Geneva; 2011.
21. Bener A, Yousafzai MT, Darwish S, Al-Hamaq AO, Nasralla EA, Abdul-Ghani M. Obesity index that better predict metabolic syndrome: body mass index, waist circumference, waist hip ratio, or waist height ratio. Journal of obesity. 2013;2013.
22. Gharipour M, Sarrafzadegan N, Sadeghi M, Andalib E, Talaie M, Shafie D, et al. Predictors of metabolic syndrome in the Iranian population: waist circumference, body mass index, or waist to hip ratio? Cholesterol. 2013;2013:198384.
23. Beydoun MA, Kuczmarski MTF, Wang Y, Mason MA, Evans MK, Zonderman AB. Receiver-operating characteristics of adiposity for metabolic syndrome: the Healthy Aging in Neighborhoods of Diversity across the Life Span (HANDLS) study. Public health nutrition. 2011;14(01):77-92.
24. Alberti G, Zimmet P, Shaw J, Grundy SM. The IDF consensus worldwide definition of the metabolic syndrome. Brussels: International Diabetes Federation. 2006;23(5):469-80.
25. Consultation WE. Waist circumference and waist-hip ratio. Report of a WHO Expert Consultation Geneva: World Health Organization. 2008;2008:8-11.
26. Grieger JA, Bianco-Miotto T, Grzeskowiak LE, Leemaqz SY, Poston L, McCowan LM, et al. Metabolic syndrome in pregnancy and risk for adverse pregnancy outcomes: A prospective cohort of nulliparous women. PLoS medicine. 2018;15(12):e1002710.
27. dos Prazeres Tavares H, Dos Santos DCDM, Abbade JF, Negrato CA, De Campos PA, Calderon IMP, et al. Prevalence of metabolic syndrome in non-diabetic, pregnant Angolan women according to four diagnostic criteria and its effects on adverse perinatal outcomes. Diabetology & Metabolic Syndrome. 2016;8:1-12.
28. Bo S, Menato G, Gallo M-L, Bardelli C, Lezo A, Signorile A, et al. Mild gestational hyperglycemia, the metabolic syndrome and adverse neonatal outcomes. Acta obstetricia et gynecologica Scandinavica. 2004;83(4):335-40.
29. Clayton GL, Borges MC, Lawlor DA. The impact of reproductive factors on the metabolic profile of females from menarche to menopause. Nature Communications. 2024;15(1):1103.
30. Mazza E, Troiano E, Ferro Y, Lisso F, Tosi M, Turco E, et al. Obesity, Dietary Patterns, and Hormonal Balance Modulation: Gender-Specific Impacts. Nutrients. 2024;16(11):1629.
31. Zheng L, Yang L, Guo Z, Yao N, Zhang S, Pu P. Obesity and its impact on female reproductive health: Unraveling the connections. Frontiers in Endocrinology. 2024;14:1326546.
32. Araújo J, Ramos E. Paediatric obesity and cardiovascular risk factors–A life course approach. Porto biomedical journal. 2017;2(4):102-10.
33. UBOS. Population and Censuses Kampala: UBOS; 2020 [Available from: https://www.ubos.org/explore-statistics/20/.
34. Sullivan K, Dean A. OPENEPI: A Web-based epidemiologic and statistical calculator for Public Health. 2009; 124: 471–474. 2009.
35. Kaduka LU, Kombe Y, Kenya E, Kuria E, Bore JK, Bukania ZN, et al. Prevalence of metabolic syndrome among an urban population in Kenya. Diabetes Care. 2012;35(4):887-93.
36. WHO. WHO STEPS surveillance manual: the WHO STEPwise approach to chronic disease risk factor surveillance. Geneva: World Health Organization; 2005. Report No.: 9241593830.
37. Petermann-Rocha F, Martínez-Sanguinetti MA, Ho FK, Celis-Morales C, Pizarro A. Optimal cut-off points for waist circumference in the definition of metabolic syndrome in Chile. Public Health Nutrition. 2020;23(16):2898-903.
38. Owolabi EO, Ter Goon D, Adeniyi OV, Ajayi AI. Optimal waist circumference cut-off points for predicting metabolic syndrome among low-income black South African adults. BMC research notes. 2018;11:1-5.

39. Nguyen KA, Peer N, de Villiers A, Mukasa B, Matsha TE, Mills EJ, et al. Optimal waist circumference threshold for diagnosing metabolic syndrome in African people living with HIV infection. Plos one. 2017;12(9):e0183029.
40. Lubogo D, Wamani H, Mayega RW, Mutungi G, Ssenyonga R, Orach CG. Metabolic syndrome and associated factors among females of reproductive age in Wakiso district, central Uganda: A cross sectional study. 2024.
41. StataCorp L. StataCorp stata statistical software: Release 14. StataCorp LP: College Station, TX, USA. 14.1 ed2015.
42. Fluss R, Faraggi D, Reiser B. Estimation of the Youden Index and its associated cutoff point. Biometrical Journal: Journal of Mathematical Methods in Biosciences. 2005;47(4):458-72.
43. Andreu A, Casals G, Vinagre I, Flores L. Obesity management in women of reproductive age. Endocrinología, Diabetes y Nutrición (English ed). 2023;70:85-94.
44. Grover S, Brandt J, Reddy U, Ananth C. Chronic hypertension, perinatal mortality and the impact of preterm delivery: a population-based study. BJOG: An International Journal of Obstetrics & Gynaecology. 2022;129(4):572-9.
45. Alberti, Kurt George Matthew Mayer, Zimmet Paul, Shaw. J. Metabolic Syndrome—a new world-wide definition. A consensus statement from the International Diabetes Federation. Diabetic medicine. 2006;23(5):469-80.
46. Magalhaes P, Capingana DP, Mill JG. Prevalence of the metabolic syndrome and determination of optimal cut-off values of waist circumference in university employees from Angola. South African Journal of Diabetes and Vascular Disease. 2015;12(1):35-40.
47. Cheong KC, Ghazali SM, Hock LK, Yusoff AF, Selvarajah S, Haniff J, et al. Optimal waist circumference cut-off values for predicting cardiovascular risk factors in a multi-ethnic Malaysian population. Obesity research & clinical practice. 2014;8(2):e154-e62.
48. Ogawa D, Kahara K, Shigematsu T, Fujii S, Hayakawa N, Okazaki M, et al. Optimal cut-off point of waist circumference for the diagnosis of metabolic syndrome in Japanese subjects. Journal of Diabetes Investigation. 2010;1(3):117-20.
49. Sinaga M, Worku M, Yemane T, Tegene E, Wakayo T, Girma T, et al. Optimal cut-off for obesity and markers of metabolic syndrome for Ethiopian adults. Nutrition journal. 2018;17(1):1-12.
50. Tladi D, Mokgatlhe L, Shaibu S, Nell T, Mitchell R, Mokgothu C, et al. Determination of optimal cut-off values for waist circumferences used for the diagnosis of the metabolic syndrome among Batswana adults (ELS 32). Cardiovascular Journal of Africa. 2020;31(6):314-8.
51. Motala AA, Esterhuizen T, Pirie FJ, Omar MA. The prevalence of metabolic syndrome and determination of the optimal waist circumference cutoff points in a rural South African community. Diabetes care. 2011;34(4):1032-7.
52. Casari S, Di Paola M, Banci E, Diallo S, Scarallo L, Renzo S, et al. Changing dietary habits: the impact of urbanization and rising socio-economic status in families from Burkina Faso in Sub-Saharan Africa. Nutrients. 2022;14(9):1782.
53. Subramoney S, BjöRkeluND C, Guo X, Skoog I, Bosaeus I, Lissner L. Age-related differences in recommended anthropometric cut-off point validity to identify cardiovascular risk factors in ostensibly healthy women. Scandinavian journal of public health. 2014;42(8):827-33.
54. St-Onge M-P, Gallagher D. Body composition changes with aging: the cause or the result of alterations in metabolic rate and macronutrient oxidation? Nutrition. 2010;26(2):152-5.
55. Kohn MA, Newman TB. The walking man approach to interpreting the receiver operating characteristic curve and area under the receiver operating characteristic curve. Journal of Clinical Epidemiology. 2023;162:182-6.
56. Shan G. Improved confidence intervals for the Youden index. PloS one. 2015;10(7):e0127272.

Supporting Information

S2 Fig: ROC curve for 18-49 years old females in Wakiso district, 2023

S3 Fig: ROC curve for 15-17 years old females in Wakiso district, 2023

S4 Fig: ROC curve for 18-24 years old females in Wakiso district, 2023

S5 Fig: ROC curve for 25-34 years old females in Wakiso district, 2023

S6 Fig: ROC curve for 35-44 years old females in Wakiso district, 2023

S7 Fig: ROC curve for 45-49 years old females in Wakiso district, 2023

 Click here to access/download;Figure;S7 Fig.tif

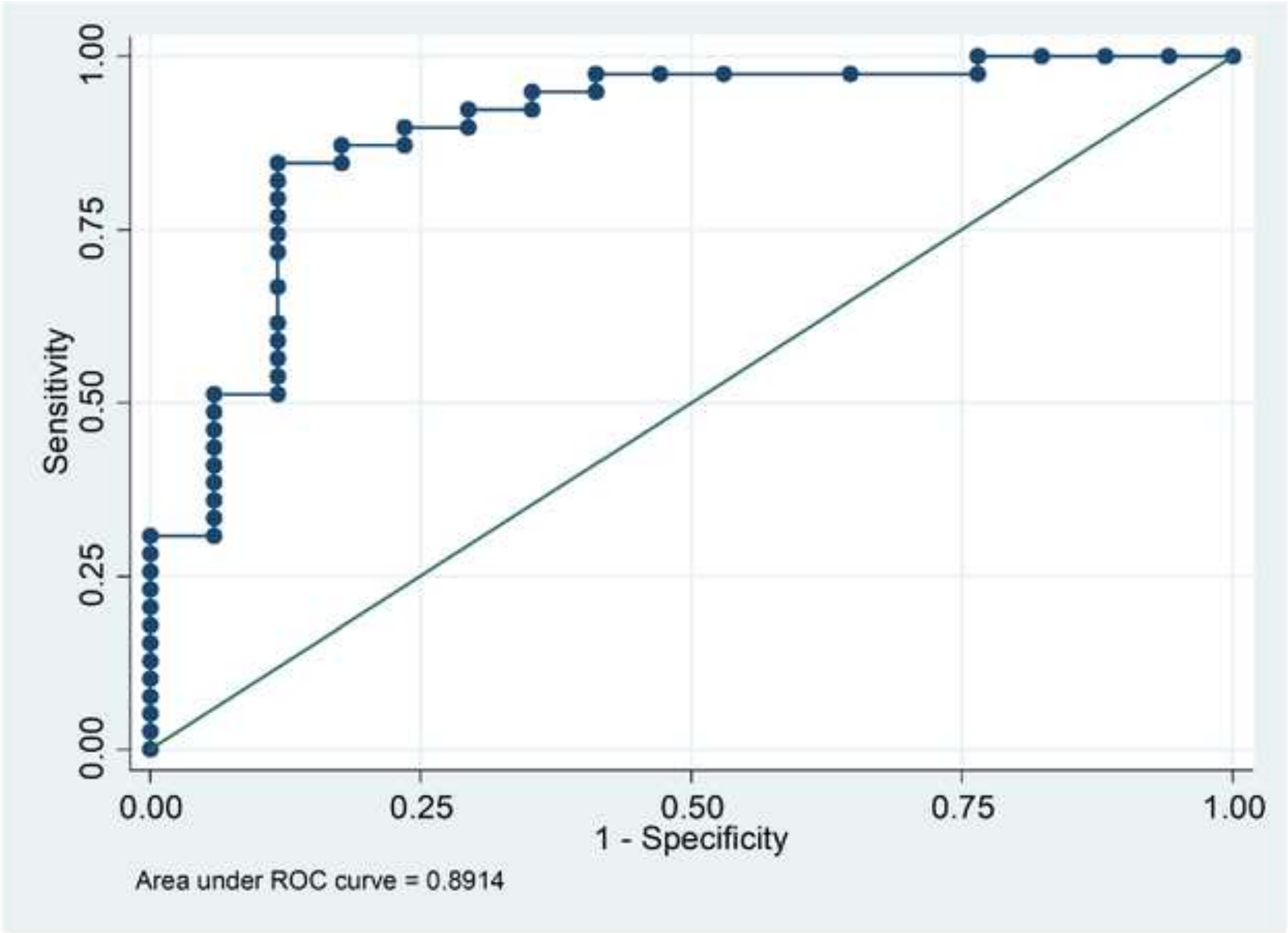

S7 Fig: ROC curve for WC to predict the presence of 2 or more MetS risk factors in females of reproductive age (45-49 years) in Wakiso district, 2023

Click here to access/download;Figure;S2 Fig.tif

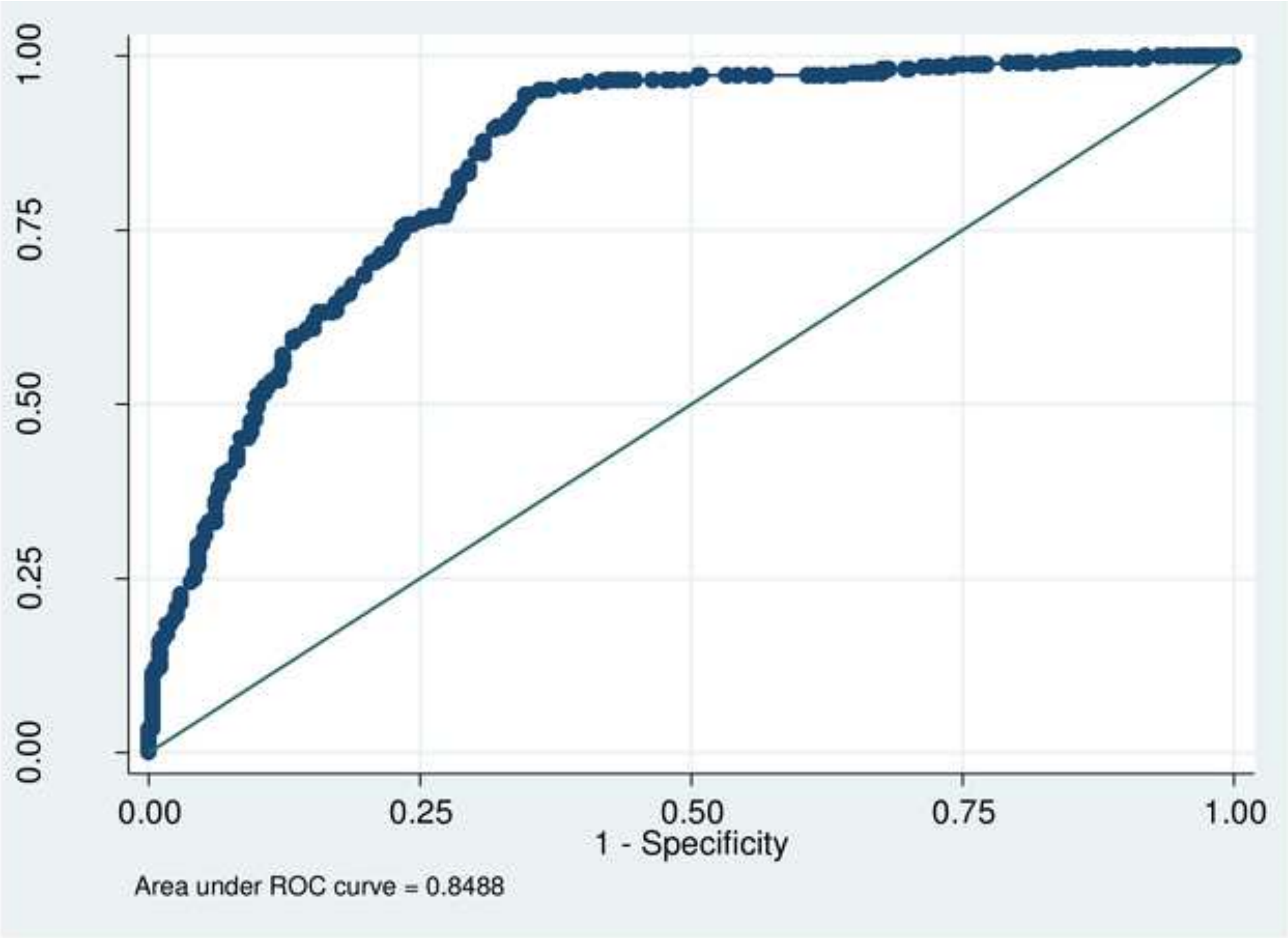

S2 Fig: ROC curve for WC to predict the presence of 2 or more MetS risk factors in females of reproductive age (18-49 years) in Wakiso district, 2023

Click here to access/download;Figure;S4 Fig.tif

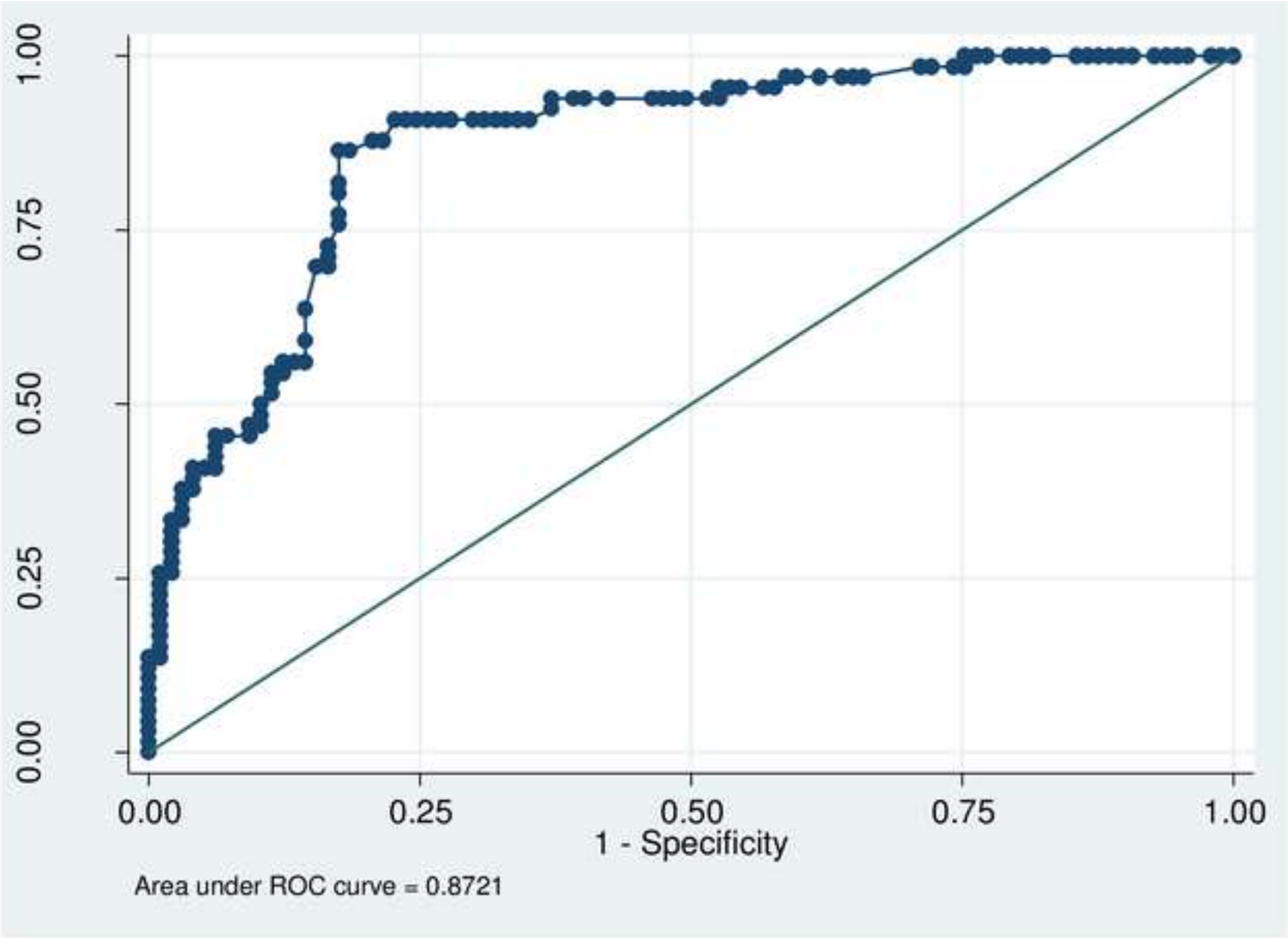

S4 Fig: ROC curve for WC to predict the presence of 2 or more MetS risk factors in females of reproductive age (18-24 years) in Wakiso district, 2023

Click here to access/download;Figure;S1 Fig.tif

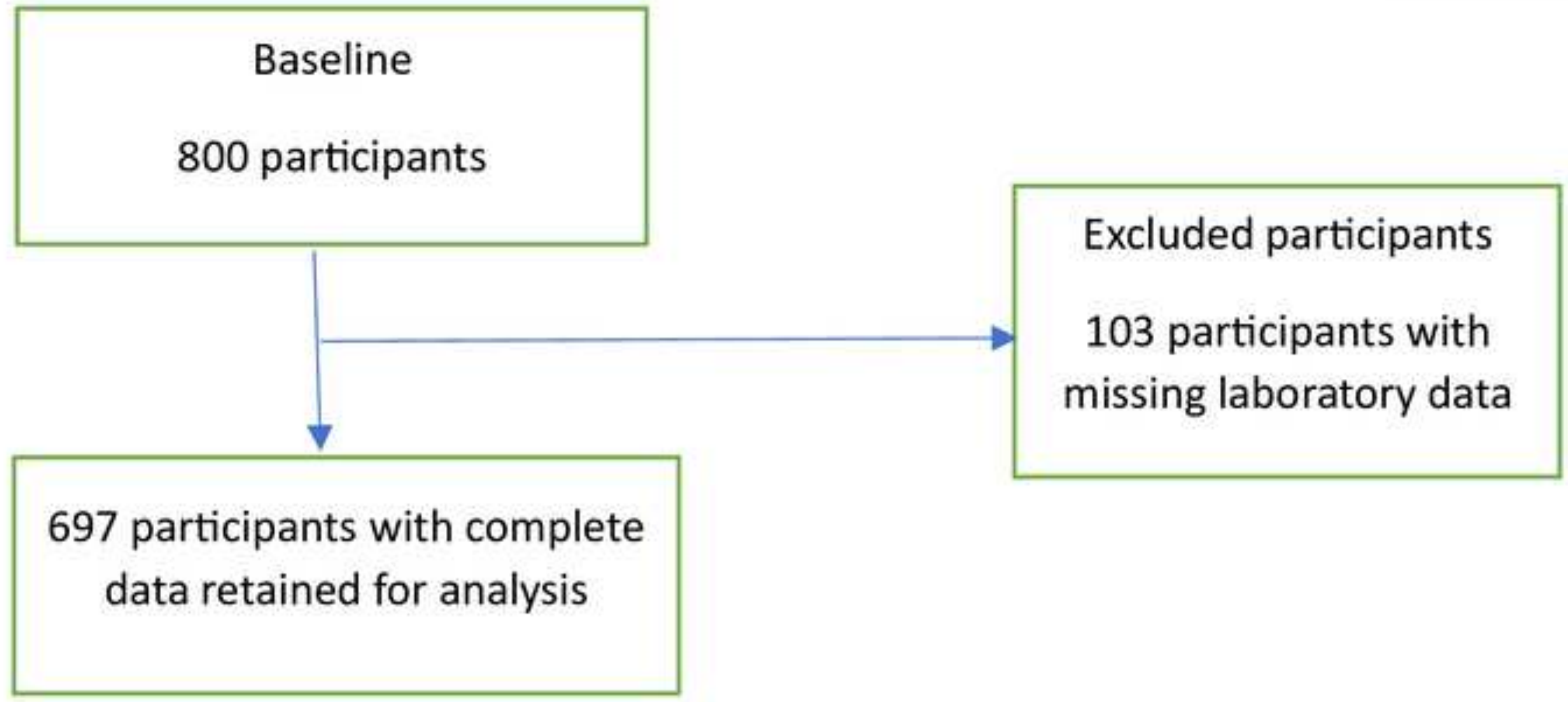

S1 Fig: Flow diagram of study participants

 Click here to access/download;Figure;S3 Fig.tif

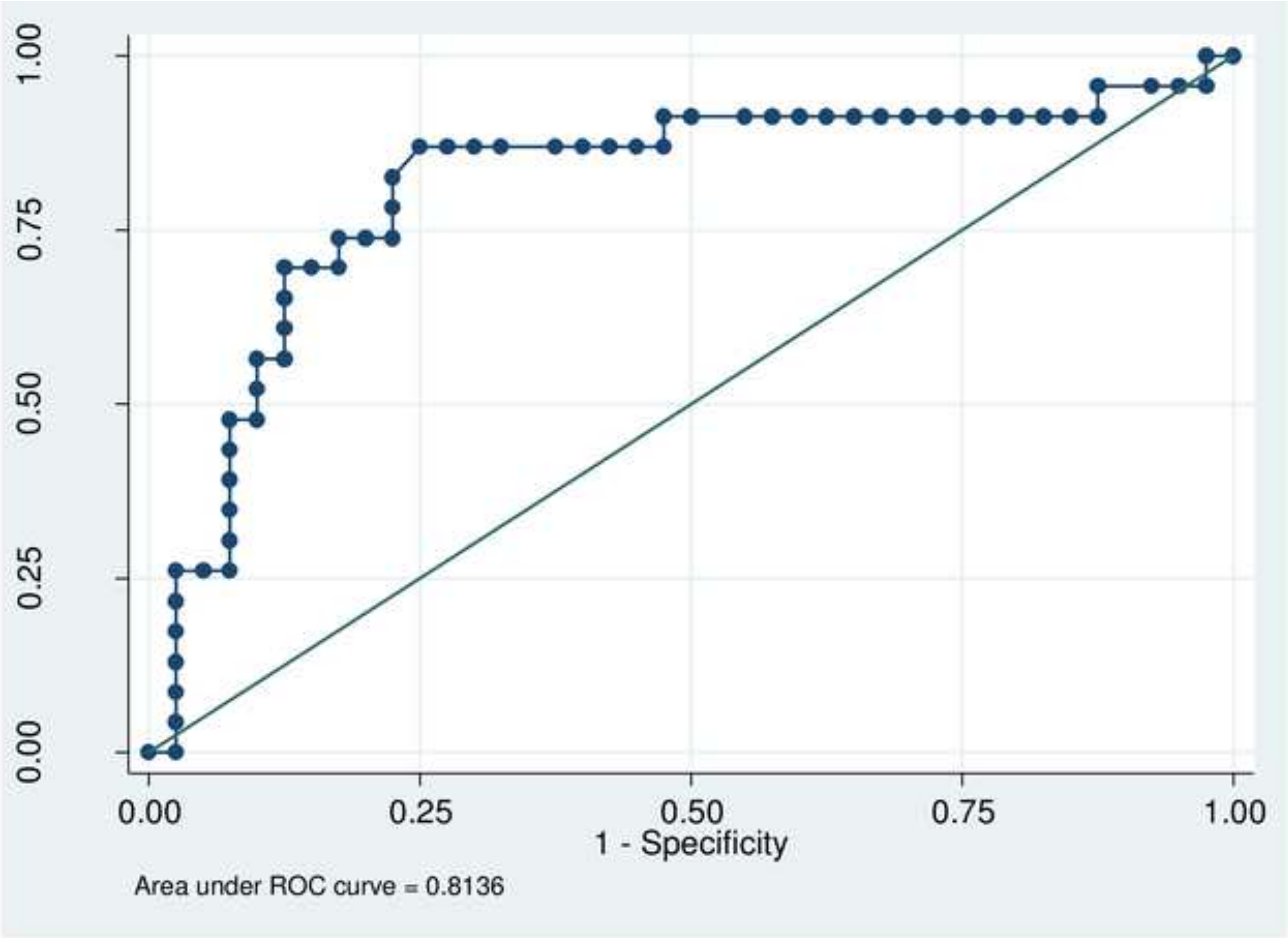

S3 Fig: ROC curve for WC to predict the presence of 2 or more MetS risk factors in females of reproductive age (15-17 years) in Wakiso district, 2023

Click here to access/download;Figure;S5 Fig.tif

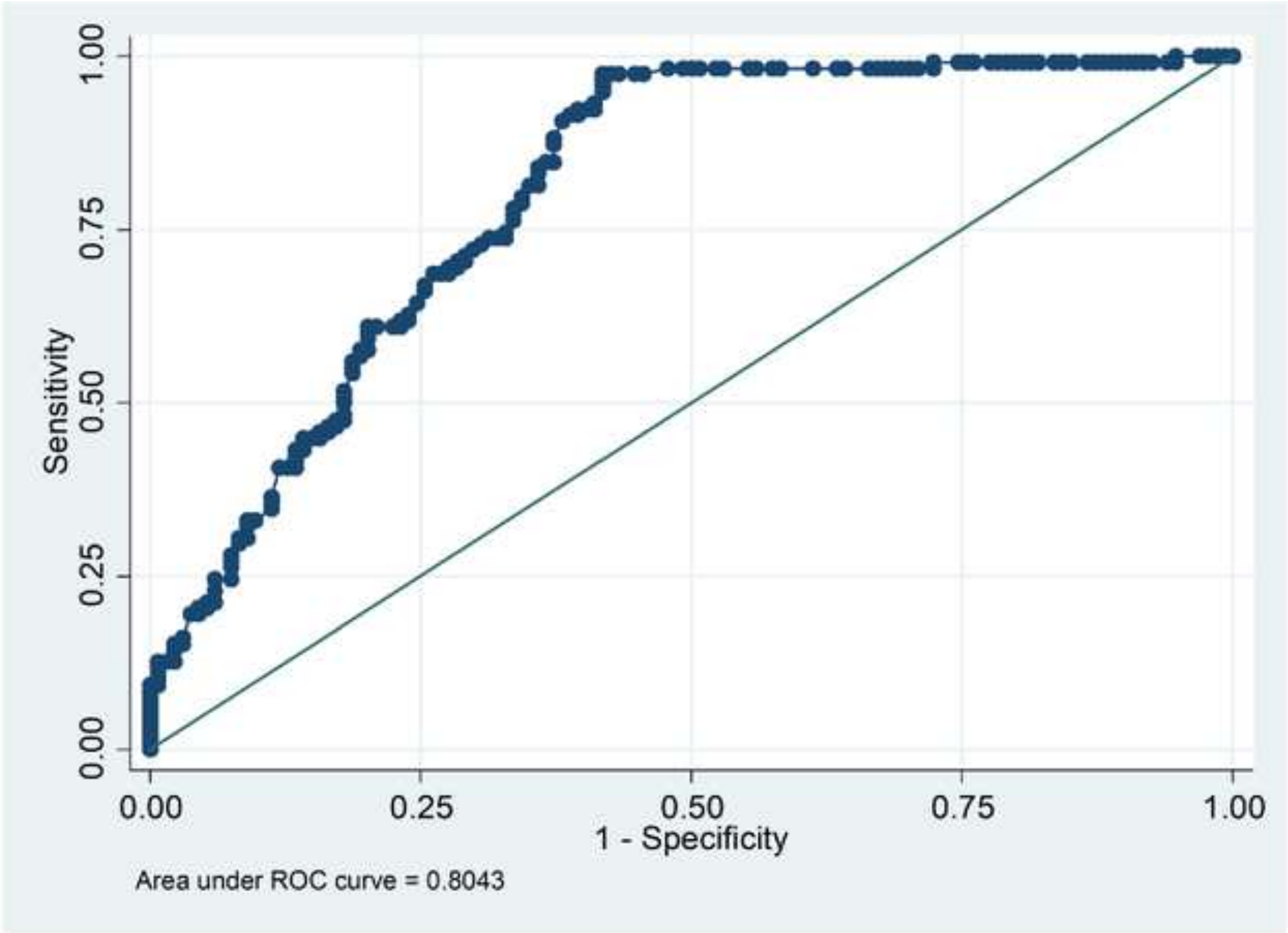

S5 Fig: ROC curve for WC to predict the presence of 2 or more MetS risk factors in females of reproductive age (25-34 years) in Wakiso district, 2023

 Click here to access/download;Figure;S6 Fig.tif

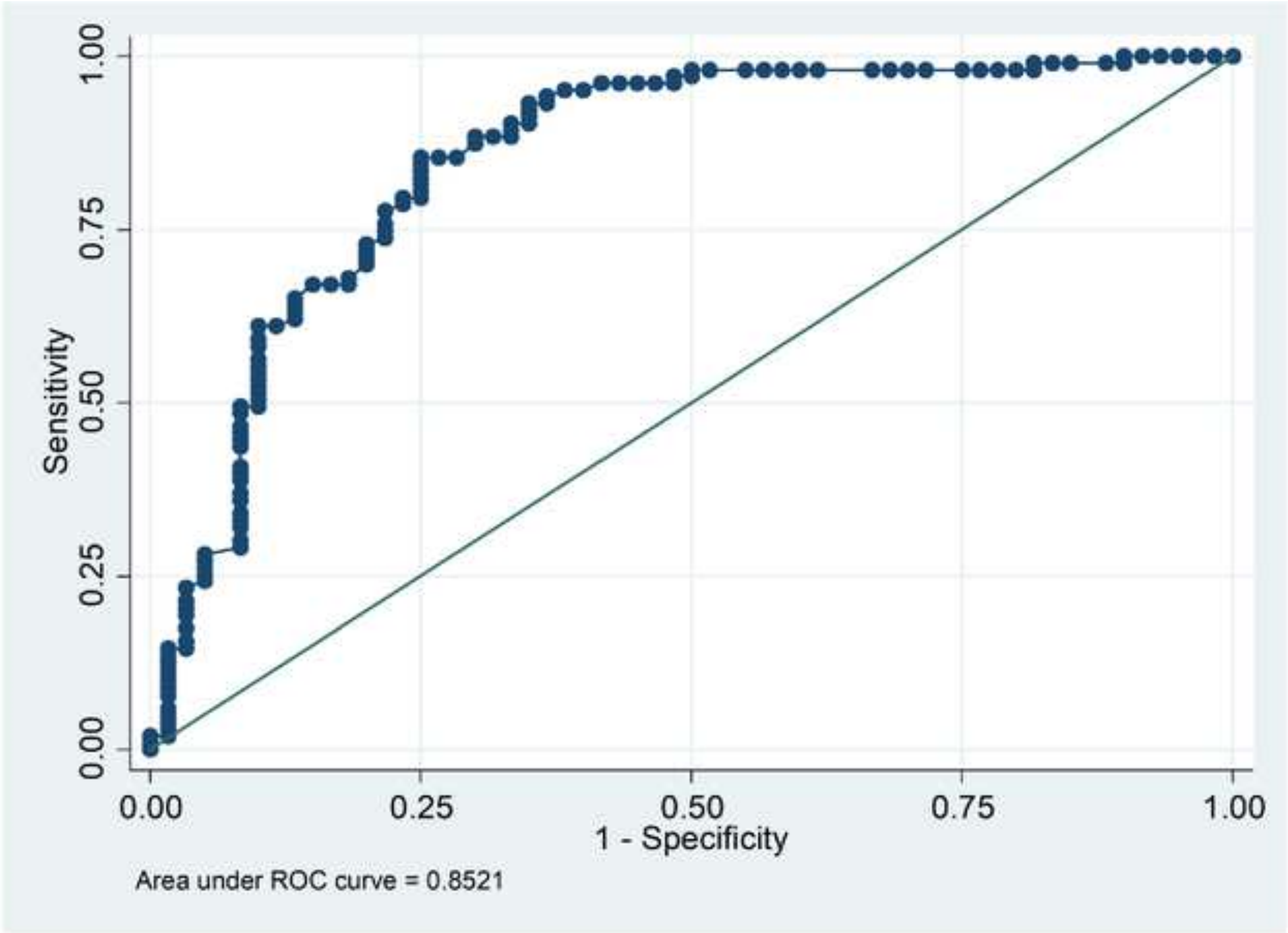

S6 Fig: ROC curve for WC to predict the presence of 2 or more MetS risk factors in females of reproductive age (35-44 years) in Wakiso district, 2023

Data set for optimal WC cut-off points

Click here to access/download
Supporting Information
Data for optimal WC cut off points.xlsx

Optimal waist circumference cut-off points for predicting m~~M~~etabolic s~~S~~yndrome among females of reproductive age in Wakiso district, central Uganda

Authors

David Lubogo[1*], Henry Wamani[1], Roy William Mayega[2], Christopher Garimoi Orach[1]

Affiliations

[1]Department of Community Health and Behavioural Sciences, School of Public Health, College of Health Sciences, Makerere University, Kampala, Uganda

[2]Department of Epidemiology and Biostatistics, School of Public Health, College of Health Sciences, Makerere University, Kampala, Uganda

* Corresponding author

Lubogodad@yahoo.com

Abstract

Background

Metabolic syndrome (MetS) poses a significant challenge to global public health, due to its strong association with Type 2 diabetes and cardiovascular disease. Waist circumference (WC) is a convenient metric for diagnosing MetS. Our study sought to establish waist circumference cut-off points that predict MetS among females of reproductive age in Wakiso district, central Uganda.

Methods:

The data collected were from a cross-sectional study conducted in Wakiso district, central Uganda, involving 697 randomly selected females aged 15 to 49, between 9th June 2021 and 17th August 2021. Data included MetS components: WC, High-Density Lipoprotein (HDL) Cholesterol, triglycerides, blood pressure, and fasting blood sugar. MetS status was identified based on two or more MetS components excluding WC. ROC analysis established the average optimal WC. The accuracy and performance of the cut-off points were evaluated through sensitivity, specificity, Positive likelihood ratio, and the Youden index.

Results:

Of the 697 participants, 50.1 % had two or more MetS risk factors. The average optimal WC cut-off for females aged 18-49 years was 80.3 cm. Variations in optimal WC thresholds were observed across different age groups: ~~78.9 cm for (15-17 years),~~ 80.4 cm (18-24 years), 79.9 cm (25-34 years), 85.6 cm (35-44 years), and 91.1 cm (45-49 years) respectively. The area under the ROC curve at the cut point for these age groups ranged from 0.78 to 0.86, indicating good discriminatory capability. The sensitivity ranged from 85% to 97%, specificity from 58% to 88%, and the Youden Index from 0.557 to 0.729.

Conclusions:

A waist circumference of 80.3 cm was the optimal threshold for identifying metabolic syndrome in females between the ages of 18 and 49 years in the setting. This finding concurs with the guidance set forth by the International Diabetes Federation.

Additionally, study participants had varied WC cut-offs ranging from 79.94 cm to 91.1 cm, depending on their age.

Keywords: Cut-off points, females, optimal, reproductive age, waist circumference

Introduction

Metabolic syndrome (MetS) corresponds to the combination of several disorders closely related to abdominal obesity, (1)~~,~~ which is a strong predictor of adverse metabolic outcomes including insulin resistance, hypertension, and dyslipidemia (2, 3).

There are several~~various~~ definitions for MetS. However, according to the International Diabetes Federation (IDF), a diagnosis of MetS requires the presence of abdominal (central) obesity, measured by waist circumference, with ethnic-specific thresholds of~~,~~ ≥ 94cm and ≥ 90 cm for European and Asian men, respectively, and ≥ 80 cm for women of all ethnic groups ~~in women~~, and at least two of the following four factors, reduced HDL Cholesterol, elevated triglycerides, high blood pressure, and elevated fasting blood sugar, (4-6).

Metabolic syndrome is a significant global public health challenge, contributing to high morbidity and mortality, with ~~and~~ its prevalence increasing in ~~occurrence is growing~~ in Africa (7-10). The failure to address MetS significantly increases the chances of developing Type 2 diabetes and cardiovascular disease (11-13). Therefore, early identification of cases and management are key in preventing disease progression and the associated long-term health consequences (14).

The measurement of waist circumference (WC) serves as a simple and practical indicator for assessing abdominal obesity, which is a key component of metabolic syndrome (15, 16). The World Health Organization (WHO) and other scientific organizations strongly recommend using waist circumference measurement in screening, diagnosis, and routine clinical care to guide patient management (5, 17). This requires an acceptable waist circumference cut-off for risk stratification. However, establishing universally accepted WC cut-offs has been challenging due to differences in abdominal tissue composition among ethnic groups (18). This is because genetics, environmental, and lifestyle elements impact abdominal obesity (19) and overall body composition (20).

Waist circumference is a simple, useful and superior (21-23) predictor for MetS compared to body mass index. Its simplicity and ability to capture central obesity make it a widely used tool (24) in both clinical and public health practice. Abdominal adipose tissue positively correlates with waist circumference and waist-hip ratio and is associated with several metabolic abnormalities including adverse lipid profiles, decreased insulin sensitivity, and decreased glucose tolerance all of which are risk factors for T2DM and CVD.

The current waist circumference cut-off points for predicting metabolic syndrome vary by population and guidelines. For instance, according to the International Diabetes Federation (IDF) guidelines, the waist circumference cut-off for men is ≥ 94 cm for Europeans and ≥ 90 cm for South Asians, Chinese and Japanese. For women, the waist circumference is ≥80 cm for all ethnicities (24). The Sub-Saharan Africans use European waist circumference cut-offs (24). On the other hand, the WHO Expert Consultation (2008) cut-off points for men are ≥ 94 cm and ≥80 cm for women (25).

Metabolic syndrome is more prevalent among females than males (8, 11), directly affects their reproductive roles, and contributes to adverse maternal (26) and perinatal (27, 28) outcomes.

Women of reproductive age (15-49 years) experience distinct physiological and hormonal changes that influence their health and metabolic health (29). Hormonal fluctuations due to the menstrual cycle, pregnancy, and menopause, as well as reproductive health conditions such as polycystic ovary diseases (PCOS), can alter body fat distribution (30), leading to abdominal obesity. This abdominal obesity, along with its associated metabolic abnormalities, can directly affect women's reproductive health (31). Obesity in childhood can also lead to the development of metabolic diseases such as diabetes or cardiovascular disease later in life (32).

Uganda and other countries in sub-Saharan Africa use WC cut-offs endorsed by international scientific organizations including the IDF and WHO (4, 20). Nevertheless, it is still unclear whether established thresholds, such as the ≥ 80 cm suggested for females, are valid and

appropriate for the African population. Hence, it the necessary to conduct research on the diverse African population groupings including Uganda and to establish sex-specific WC cut-offs as recommended by WHO and others (5, 20).

To date, most studies on optimal waist circumference cut-off points have been conducted in high-income countries. In low-income countries such as Uganda, the optimal ethnic WC cut-off has not been established particularly for females of reproductive age. Given the unique metabolic challenges faced by women of reproductive age, determining optimal WC cut-off points that are sensitive to their specific physiological context is warranted to improve early detection and management of the condition.

Therefore, this study aimed at determining the average waist circumference and age-specific cut-off points that are optimal for predicting MetS among females of reproductive age in Wakiso district, central Uganda. This may assist in the early detection, prevention and management of metabolic syndrome. The hypothesis for the study was that the optimal waist circumference for females aged 15-49 years might not be equal to 80.0cm.

Methods

Study design

We carried out a cross-section study in Wakiso district between 9th June 2021 and 17th August 2021. Our study involved a random sample of female participants from 15 to 49 years of age.

Study population

The study population included females aged 15-49 years residing in Wakiso district, central Uganda. Participants were randomly selected from urban and rural communities to ensure diversity. Eligible participants had resided in Wakiso for at least a year and were identified from a database of 800 individuals, who had anthropometric, physical, and biochemical tests carried out on them to determine the prevalence of MetS in the district.

We excluded participants who were pregnant, those within six weeks after childbirth, alcoholics, smokers, those with chronic diseases such as diabetes mellitus, hypertension, hyperlipidaemia, allergic disorders, depression, and those with known carrier states of hepatitis B, hepatitis C, or human Immunodeficiency virus.

Formatted: Font: Not Bold

Study setting

The study was conducted in Wakiso district, situated in central Uganda, with an estimated total population of 3,105,700, based on the 2014 census. More than half (52.7%) of the residents are females. Of these, 793,770 are in the reproductive age bracket of 15-49 years (33). The district comprises two counties, namely Kyaddondo and Busiro, and four Municipal Councils - Entebbe, Nansana, Kira, and Makindye-Ssabagabo that together constitute eight sub-counties, eight town councils, 148 parishes, and 704 villages. The Uganda Bureau of Statistics provided the sampling frame for the study, and it included 32 randomly selected enumeration areas in the district. Twenty-eight (28) households were sampled per enumeration area.

Sample size calculation

The sample size was computed using the OpenEpi, Version 3.01, open-source calculator for a proportion or descriptive study (34). Using a population size for a finite population of 1,000,000, confidence limits of 5%, a design effect of 2.0., and a prevalence (p) of 40.2% +/-5 obtained from a study conducted among females in an urban population in Kenya to determine MetS prevalence (35). A sample size of 739 was obtained; after adjusting for a response rate of 90%, the sample size was 813. The final sample used in the study was 800 respondents. We estimated a MetS prevalence of 15% and expected 120 respondents to be with MetS out of the total sample size of 800. This sample was considered appropriate for assessing the sensitivity and specificity of WC as a predictor for MetS in this sample.

Measurements and variables

We used the population sample data set from a prevalence study to obtain participant data on anthropometric measurements (waist circumference), physical measurements (blood pressure), and biochemical measurements (fasting plasma glucose, triglycerides, and low HDL Cholesterol) collected as per the WHO STEP wise approach (36).

Quality control measures were undertaken to ensure the data for the prevalence study were of quality. They included the employment of well-trained data collectors/research assistants (female nurses and graduate nutritionists) who were knowledgeable of the study protocol and had skills in using the Open Data Kit (ODK) for data collection. The data collection tools were field tested from a district near the study district. The data collected in the field were entered in the ODK tool embedded with skip patterns and constraints to ensure data quality and reduce bias. Quantitative data (ODK data) was downloaded from the server and exported to Stata version 14 for cleaning. This ensured reasonable quality control and data management for this study.

Study variables

Dependent variable:

Metabolic syndrome wa~~i~~s assessed by determining if an individual had~~s~~ three or more of the five components namely: low HDL cholesterol (<50 mg/dL or (1.3 mmol/l) in female) or treatment for it, high triglycerides (≥150 mg/dL (1.7 mmol/L) or treatment for it, elevated fasting blood sugar (≥ 100 mg/dL (5.6 mmol/l) or diabetes diagnosis, or diabetes treatment; high blood pressure (≥130/ ≥85 mmHg) or treatment for it, and increased waist circumference. These components we~~a~~re derived from the Joint Interim Statement (JIS) (5). This study assessed MetS using two of the four metabolic components, excluding waist circumference. This approach was used to evaluate the independent contribution of waist circumference to metabolic risk. Similar methods have been applied in previous studies (37-39).

Detailed information on the measurements of the variables used in the baseline characteristics can be found in Lubogo etal, 2024 (40).

Data analysis

Data were analysed using Stata statistical software (SE/14.0, StatCorp, College Station, TX, USA (41). Descriptive statistics were employed to summarise participant characteristics. Data on continuous variables were summarised using the independent sample T-test and categorical variables were analysed using the Chi-square test. The results were presented as means with standard deviation and frequencies/percentages.

Waist circumference cut-off points for various age groups (15-17, 18-24, 25-34, 35-44, 45-49, 15-49, 18-49, years) were evaluated using sensitivity and specificity techniques to identify the most effective predictive values for MetS within the study age groups.

The research investigated the Area under the Curve (AUC) for different components of MetS such as elevated fasting blood sugar, high triglycerides, high blood pressure and low HDL cholesterol. To conduct this analysis, an ROC curve was plotted for the association between waist circumference (WC) and two or more components of MetS.

The optimal cut-off points for WC, which achieve maximum accuracy, were determined by plotting sensitivity and (1-specificity) on a graph.

The study employed the Youden index method (42) expressed as [Maximum (sensitivity + specificity - 1)] to ascertain the optimal WC values and also calculated the positive predictive values (PPV) and negative predictive values (NPV).

Ethical considerations

We obtained approval from the Higher Degrees Research and Ethics Committee of the School of Public Health, College of Health Sciences, Makerere University (Protocal number: MakSPH-REC, 071) and the National Council for Science and Technology of Uganda (Registration number: UNCST, HS1281ES). All study participants provided written informed consent and were assured of the confidentiality of their information. Written informed consent was obtained from parents or guardians of participants under 18 years of age.

Results

Flow of participants

The figure in S1 Fig shows the flow diagram of the study participants.

S1 Fig: Flow diagram of study participants

Characteristics of study participants

The study involved 697 women of reproductive age with a mean age of 29.7 ± 9.07 years. Most participants (38.7%) completed primary school, and 51.5% were employed. ~~The mean body mass index (BMI) was 26.9 (±5.8), 43% of the participants had a normal BMI, and 27.6% were obese. See Table 1.~~

For participants aged 18-49 years, the mean body mass index (BMI) was 27.2 (±5.8), 40.1% had a normal BMI, and 28.9% were classified as obese. See Table 1.

Formatted: Font: Not Bold

Formatted: Font: Not Bold

I noticed that the results section is missing important details such as the distribution of BMI percentiles for young adult(15-17 yrs) education level, physical activity, and salt intake. These key aspects are mentioned but not clearly presented in the results. Could you please provide a more comprehensive presentation of these factors with the appropriate distribution and clarity? This would improve the completeness and readability of the results

Table 1: Characteristics of women of reproductive age in Wakiso district (N=697)

Characteristics	N	Percent
All participants	697	100.0
Age group (n) in years (mean, SD)	~~29.7 (SD=9.07)~~	
15-17 (n=63)	16.0 ± 0.77	
18-24 (n=163)	21.2 ± 2.06	
25-34 (n=252)	29.0 ± 2.7	
35-44 (n=163)	38.8 ± 2.87	
45-49 (n=56)	46.7 ± 1.70	
15-49 (n=697)	29.7 ~~(SD=~~±9.07)	
18-49 (n=634)	31.1± 8.36	
Level of Education attained		
Primary not completed	166	23.8
Primary School	270	38.7
0-Level[a]	154	22.1
A-Level[b] or above	107	15.4
Employment Status		
Paid and employed	359	51.5
Unpaid and Unemployed	338	48.5
BMI-for-age percentiles (n=63)*		
< 5 th percentile	0	0
5th-<85th percentile	37	58.8
85th-<95th percentile	13	20.6
≥ 95th percentile	13	20.6
BMI[a] (kg/m^2) (Mean, SD) (n=634)**	27.2~~26.9~~ ~~(~~± ~~SD=~~5.8~~)~~	
< 17.0	0	0
< 18.5	11~~20~~	1.7~~2.9~~
18.5–24.9	254~~300~~	40.1~~43.0~~
25.0–29.9	186~~185~~	29.3~~26.5~~
≥ 30	183~~192~~	28.9~~27.6~~
Fruits and vegetables consumed per day		
< 5 servings	488	70.0
≥ 5 servings	209	30.0
Physical Activity		
Insufficient physical activity	550	78.9
Moderate physical activity	61	8.8
Vigorous physical activity	86	12.3
Current alcohol users (Past 30 days)		
Non-current drinker	605	86.8
Lower end level (< 2 drinks /occasion)	27	3.9
Intermediate end level (2 - < 4 drinks per occasion)	52	7.4
High-end level ≥4 drinks per occasion)	13	1.9
Tobacco Use		

Question to the Author:
In your analysis, the age group (15–49 years) is presented with significant variation in sample size distribution across different age ranges (e.g., 15–17 years with 63 participants versus 25–34 years with 252 participants). Given the disparity in age group sizes, could this imbalance have affected the generalizability or representativeness of the findings? Additionally, was any weighting or stratification method applied to account for this variation? If not, it would be beneficial to address this limitation in the discussion section, as it could influence the overall conclusions.

Formatted: Right

Formatted: Right

Formatted: Font: (Default) Times New Roman, 12 pt

Formatted: Right

Formatted: Right

Formatted: Right

The inclusion of education levels in this paper may not be relevant, as no associations with abdominal obesity or other risk factors are being analyzed. Additionally, the mix of school-going individuals and early adults makes the classification unclear. Would it be more appropriate to remove this information to improve the clarity and focus of the manuscript?

Formatted: Superscript

Formatted: Superscript

Formatted: Left

Formatted Table

There is no need to mention the 0% category for BMI-for-age percentiles and BMI ranges. It would improve the clarity and conciseness of the data presentation if these values are omitted.

Your descriptive table can be more informative and supportive of the main analysis by aligning it with your hypothesis, which focuses on stratification. Consider presenting demographic information classified by age categories. This will help identify potential confounding factors, making it easier to adjust in association analyses. Additionally, the absence of association analysis is a significant limitation. Including this analysis would strengthen the manuscript's overall meaning and analytical depth.

Non-smokers (past 30 days)	689	98.9
Smokers (past 30 days)	8	1.1
Dietary Salt		
Adding salt or salty sauce at meal to food before or when eating		
Sometimes - never	571	82.0
Always or often	126	18.0

**BMI for age percentiles for participants aged 15-17 years old*
***BMI for participants aged 18-49 years old.*
0-Level[a] Ordinary level (lower secondary school)
A-Level[b] Advanced level (Upper secondary school)

Formatted: Font: 10 pt, Italic

Formatted: Line spacing: single

Age-specific evaluation of optimal thresholds for waist circumference using different classification metrics and prevalence of MetS components

For the age group 18~~5~~-49~~5~~ years, the optimal threshold was 80.3~~25~~ cm. The Youden I~~i~~ndex, specificity and sensitivity were 0.597 ~~0.604~~, 65 ~~67~~% and 94% respectively, and the AUC curve was 0.849~~8~~ (95% CI 0.819 - 0.879~~7~~).~~, while for 18-45 years, the optimal threshold was 80.25 cm. The Youden index, specificity and sensitivity were 0.591, 65% and 94% respectively, and the AUC curve was 0.843 (95% CI 0.812 – 0.875).~~

The optimal WC cut-off points varied among the various age groups, ranging from 78.9 cm for females aged 15 to 17 years, to 91.1 cm for those aged 45 to 49 years. Across different age groups, the area under the ROC curve at cut-point values fluctuated between 0.78 and 0.86, with the 45- 49-year-old age group showing the highest AUC of 0.86. The 45-49 years age group showed the highest discriminative power (AUC =0.891), with specificity (88 %), Youden index (0.729), and a likelihood ratio of (7.08).

The prevalence of various MetS components increased with age, particularly elevated waist circumference (p<0.001) and blood pressure (p<0.001) . Reduced HDL cholesterol (p=0.958) appeared consistent across all age groups as shown in Table 2.

The results show that HDL cholesterol, triglycerides, and fasting blood sugar (FBS) did not show any significant differences across the age groups (HDL: p=0.958, TG, and FBS not mentioned). Could you please clarify or update the results section to accurately reflect that these components did not show significant variation across age groups, as indicated by the statistical values? This would ensure the consistency and clarity of the reported findings.

Table 2: Age-stratified WC~~waist circumference~~ cut-off points, MetS risk classification metrics, and prevalence of MetS components by age group.

Variable	Age group						
	**15-17 years(n=63)**	**18-24 years (n=163)**	**25-34 years (n=252)**	**35-44 years (n=163)**	**45-49 years(n=56)**	**15-49 years(n=697)**	**18-49 years(n=634)**
**MetS risk Classification metrics**							
Empirical optimal WC cut-off point (cm)	78.9	80.4	79.9	85.6	91.1	80.3(80.25)	80.25
Area under the ROC curve at the cut point	0.81	0.84	0.78	0.80	0.86	0.80	0.80
Area under ROC curve (95% CI)	0.814 (0.690 - 0.937)	0.872 (0.817 -0.927)	0.804 (0.750 - 0.858)	0.852 (0.786 - 0.918)	0.891 (0.790 - 0.993)	0.848 (0.819 - 0.877)	0.849 (0.819 - 0.879)
Sensitivity at cut point (%)	87	86	97	85	85	94	94
Specificity at cut point (%)	75	82	58	75	88	67	65
Youden index (J)	0.620	0.688	0.557	0.604	0.729	0.604	0.597
LR+ = sensitivity / (1 - specificity)	3.480	4.778	2.3095	3.40	7.08	2.85	2.66
Presence of 2 or more metabolic syndrome risk factors (%)	36.5	40.5	46.8	63.2	69.6	50.1	51.4
**Metabolic syndrome components**							
Elevated waist circumference (%)[a]	35.9	48.0	66.8	77.3	82.8	63	66.1
Elevated triglycerides (%)[b]	10.1	13.1	12.3	15.9	24.4	14.1	14.6
Reduced HDL Cholesterol (%)[c]	44.7	48.9	48.6	47.0	41.2	47.3	47.6
Elevated blood pressure (%)[d]	14.0	21,0	19.5	35.2	42.8	25.0	26.2
Elevated fasting blood sugar (%)[e]	4.1	11.7	5.0	6.8	6.7	7.1	7.4

If you could add Height, waist hip ratio it will be use full to interpritn the data

Formatted: Font: 10 pt, Superscript

Formatted: Font: 10 pt, Superscript

Formatted: Font: 10 pt, Superscript

Formatted: Font: 10 pt, Superscript

Formatted: Font: 10 pt, Superscript

[a]($\chi2=50.94$, $df=4$,$p<0.001$), [b]($\chi2=6.62$,$df=4$,$p=0.157$), [c]($\chi2=0.65$,$df=4$,$p=0.958$), [d]($\chi2=35.88$,$df=4$,$p<0.001$), [e]($\chi2=2.91$,$df=4$,$p=0.572$)

($\chi2$= Pearson chi-square, df=degrees of freedom and p=p.value)

Formatted: Font: 10 pt, Superscript

Formatted: Font: (Default) Times New Roman, 9 pt

Formatted: Font: 9 pt

Formatted: Font: (Default) Times New Roman, 9 pt

Formatted: Font: 9 pt

Formatted: Font: 9 pt, Superscript

Formatted: Font: 10 pt, Superscript

Formatted: Font: 9 pt

Formatted: Font: 9 pt

Formatted: Font: 9 pt

Formatted: Font: 10 pt, Superscript

Formatted: Font: 9 pt

Formatted: Font: 9 pt

Formatted: Font: 9 pt

Formatted: Font: 10 pt, Superscript

Formatted: Font: 9 pt

Formatted: Font: (Default) Times New Roman, 9 pt

Formatted: Font: 9 pt

Formatted: Font: 9 pt

Formatted: Font: 10 pt, Superscript

Formatted: Font: 9 pt

Formatted: Font: 9 pt

Formatted: Space After: 8 pt, Line spacing: Multiple 1.08 li

Formatted: Font: 9 pt, Not Bold, Ligatures: Standard + Contextual

ROC curve analysis, for waist circumference in reproductive-age women in Wakiso district, for detecting MetS risk factors per age group

Figures in S2-S7 Figs show the ROC curves for WC to predict the presence of 2 or more **MetS risk factors among females of reproductive age for the various age groups.**

S2 Fig: ROC curve for 18-49 years old females in Wakiso district, 2023

S3 Fig: ROC curve for 15-17 years old females in Wakiso district, 2023

S4 Fig: ROC curve for 18-24 years old females in Wakiso district, 2023

S5 Fig: ROC curve for 25-34 years old females in Wakiso district, 2023

S6 Fig: ROC curve for 35-44 years old females in Wakiso district, 2023

S7 Fig: ROC curve for 45-49 years old females in Wakiso district, 2023

Discussion

The rise in the global prevalence of MetS calls for more research towards early and accurate diagnosis of MetS to provide preventive measures and timely intervention for MetS.

Our study revealed that the mean waist circumference cut-off points of 80.3 cm accurately predicted MetS in females aged 18-49 years in an urban population in Uganda. However, optimal waist circumference varied by age groups (15-17 years, 18-24 years, 25-34 years, 35-44 years, and 45-49 years) being 78.9 cm, 80.4 cm, 79.9 cm, 85.6 cm, and 91.1 cm respectively. This study is the first to use the Joint Interim Statement to establish these cut-off thresholds in Uganda.

The findings showed a strong link between age and the prevalence of several MetS components, particularly elevated waist circumference ($p<0.001$), and blood pressure ($p<0.001$), ~~,~~ both of which increased with age group. Reduced HDL cholesterol ($p=0.958$), appeared consistent across all age groups. Public health interventions for preventing metabolic syndrome should consider targeting abdominal obesity and hypertension, especially for older individuals within the reproductive age range. Research indicates that obesity (43) and hypertension (44) impact maternal and neonatal health.

Our study has identified an optimal WC threshold value that closely approximates the recommended cut-off of 80.0 cm as specified by the International Diabetes Federation for diagnosing metabolic syndrome (MetS) (45). Similar results were found in prior studies conducted across Africa and other locations. An optimal WC of 80.5 cm was found among female university employees in Angola (46). Elsewhere, some studies among a multiethnic Malaysian population (47) and in the Japanese population (48) found that 80.0 cm was an optimal WC for women. However, it should be noted that these two studies were conducted among ethnically different populations compared to Africans. The consistency of our findings

Formatted: Font: 12 pt

Formatted: Font: 12 pt

Formatted: Font: 12 pt

HDL cholesterol, triglycerides (TG), and fasting blood sugar (FBS) did not show significant differences across age groups. Please revise the results section to reflect this, instead of stating a reduction or change, to ensure accurate reporting of the findings. 713844e

with those of other researchers strengthens the validity of our findings and reinforces the global relevance of the established diagnostic criteria. From a public health perspective, policymakers and healthcare professionals can~~may~~ utilize these findings to implement cost-effective and efficient screening programs that facilitate~~to enable~~ the early detection and management of MetS.

ETHIOPIA	1.57 m	53.6 kg	21.8	78
BOTSWAN	1.62 m	69.8 kg	26.7	82.3
SAFRICA	1.58 m	74.8kg	29.8	92.0

~~Our findings,~~ However~~however~~, our findings differed from a study in Ethiopia, which revealed [redacted] across different geographical regions. For instance, countries with higher pre[redacted] obesity, such as South Africa, often have higher optimal WC cut-off points (15,[redacted] greater fat and different fat distribution. Economic conditions and urbanisation [redacted] lifestyle and dietary patterns (52), impacting obesity rates and waist cir[redacted] measurements.

This study also revealed varying age-specific WC thresholds for various age grou[redacted] for 15-17 years, 80.4 cm for people aged 18-24 years, 79.9 cm for those aged 25-34[redacted] cm for the 35-44 years age group, and 91.1 cm for those aged 45-49 years respectively. The age-specific cut-off points highlight the variability of MetS risk across various age groups. The younger individuals showed risk factors even with slightly lower waist circumference levels. This emphasizes the importance of early detection and timely intervention in this group. Conversely, elderly individuals exhibited higher waist circumference cut-off points. This is in agreement with other studies (53). Probable reasons for this could be due to changes in body

composition with age resulting in reduced muscle mass and gain in body fat, and a reduction in metabolic rate (54) which occurs with age resulting in weight gain if dietary intake and physical activity remained constant.

Therefore, to effectively combat the rising burden of cardiovascular diseases, healthcare providers and policymakers should include age-specific thresholds for waist circumference in screening and intervention protocols for metabolic syndrome.

Diagnostic accuracy of the waist circumference cut-off points to detect MetS risk factors

Different metrics were employed to assess the precision of detecting metabolic syndrome, including AUC, PPV, specificity and sensitivity, with reference to a specific waist circumference threshold.

When we measured the performance of the AUC values within the 15-17 years and the 18-24 age range, the AUC reached 0.81 and 0.84, respectively. This data suggests that the WC cut-off point can serve as a reasonably accurate indicator of MetS risk in individuals within this age range. For the 25-34 years age group, the AUC was 0.78, this demonstrates moderate performance. In the 35-44 years age category, the AUC was 0.80, this suggests a relatively good ability to distinguish those at risk for MetS. In the 45-49 years age group, the AUC was 0.86. This age group exhibited the highest AUC values, and thus the most robust discriminative power. This suggests that this group may be the most appropriate target for MetS screening using WC. Considering all individuals aged 15-49 years or 18-49 years, the overall AUC was 0.80, indicating good performance in identifying MetS. Normally, an AUC of 1 indicates a perfect classifier with the model yielding a 100% true positive rate and 0% false positive rate across all possible threshold values. An AUC of 0 shows that the classifier performs no better than chance, is not able to distinguish between two classes and yields a 0% true positive rate

and 100% false positive rate (55). Most classifiers aim for an AUC above 0.5, and that indicates predictive power.

These findings hold important implications for healthcare professionals and researchers, as they provide valuable guidance for developing protocols for screening MetS that are specific to different age groups.

Effectiveness of waist circumference cut-off points in terms of sensitivity and specificity

For the 15-17 age group, a waist circumference of 78.9 cm was associated with 87% sensitivity and 75% specificity. The high sensitivity suggests that it is effective in identifying metabolic syndrome. However, the moderate specificity indicates some potential for false positives in that age group.

Considering sensitivity and specificity values, ~~in the 18-24 age group,~~ a WC of 80.4 cm in the 18-24 age group showed ~~was obtained witha~~ 86% sensitivity and 82% specificity. The high sensitivity further~~also~~ suggests that its effectiveness~~ is effective~~ in identifying metabolic syndrome.

The 25-34 age group showed a WC cut-off of 79.9 cm and had the highest sensitivity of 97%. This means that nearly all individuals with metabolic syndrome in this age group are accurately identified. However, a lower 58% specificity may imply concerns about potential false positives, and thus there is a need to do confirmation tests.

The 35-44 years age category had an 85.6 cm cut-off, and balanced sensitivity (85%) and specificity (75%). This implies that it is reasonably practical in identifying those with metabolic syndrome and can minimize the risk of false positives, making it a valuable tool for screening.

In the 45-49 age group, there was a 91.1 cm cut-off point, with an 85% sensitivity and 88% specificity. This implies that this cut-off can accurately identify individuals at risk and can

minimise false positives. For the age range of 15-49 years, and 18-49 years, we obtained an 80.325 cm cut-off point, with 94% sensitivity and 67% specificity, and 94% sensitivity and 65% specificity, respectively. This shows thatthat it has the potential to serve as an efficient screening tool. However, the lower specificity of 65% and 67% calls for further evaluation to confirm diagnoses.

Generally, these age-specific WC cut-off points can be used to offer tailored strategies for MetS risk assessment and highlight the importance of age in risk prediction and patient management. Further research may be needed to validate and expand these findings for broader clinical and public health applications.

Youden Index and Positive Likelihood Ratios (LR+)

Our study evaluated age-specific waist circumference (WC) cut-off points and their associated Youden index (J) and Positive Likelihood Ratios (LR+) to determine the diagnostic performance across different age groups.

Our study identified distinctions among the different age categories. The 45-49-year-old age group showed the highest Youden index (J) of 0.729, indicating a higher overall accuracy than the other age groups. A value of one (1) indicates perfect accuracy, and the closer J is to 1, the better the test's overall performance (56). Furthermore, it showed the highest LR+ value of 7.08. This implies that among individuals within this age group, a positive test result is about 7.08 times more likely to occur in individuals with metabolic syndrome compared to those without the syndrome. This makes it a robust diagnostic tool.

The 25-34 years age group showed a slightly lower Youden index (J) of 0.557 (indicating slightly lower overall accuracy) compared to the 18-24 years group with a Youden index (J) of 0.688. The 25-34 years age group showed an LR+ of 2.3095, demonstrating valuable diagnostic utility. A LR+ of 2.3095 suggests that individuals diagnosed with metabolic syndrome have a

waist circumference above the cut-off point approximately 2.3 times more often than those without metabolic syndrome. Although this LR+ was slightly lower than the 18-24 years group LR+ (Likelihood Ratio Positive) of 4.788, this LR+ is still a valuable indicator of diagnostic strength. The 35-44 years and the broader 15-49 years age range both demonstrated intermediate Youden index (J) values of 0.604, with corresponding LR+ values of 3.4 and 2.85, respectively.

The study demonstrates that for urban Ugandan females aged 18-49 years, 80.3 cm waist circumference is the mean optimal threshold for MetS prediction. However, these findings were based on data from one district and should be interpreted cautiously when generalizing to other populations. Furthermore, waist circumference cut-off points varied depending on age group as indicated; 78.9 cm (15-17 years),80.4 cm (18-24 years), 79.9 cm (25-34 years), 85.6 cm (35-44 years), and 91.1 cm (45-49 years). Our findings provide useful information for healthcare professionals when considering age-tailored risk assessment strategies for metabolic syndrome to enhance the clinical relevance of WC measurements.

Study limitations

We used a cross-sectional study design. MetS is a condition that develops over time, and the measurement of WC in a single time point may not fully capture its dynamic nature. A longitudinal study would be more appropriate for confirming the temporal relationships observed in the study findings.

Our study focused exclusively on females of reproductive age in Wakiso district, central Uganda. Consequently, while the findings are relevant to women in a similar setting, the results may not be generalizable to the entire Ugandan population. Conducting nationally representative studies on optimal waist circumference in the future would help address this limitation and provide a better understanding of MetS in Uganda.

Our study sample was randomly sampled from an existing database. While random sampling ensures diversity, it may miss out on segments of the population not included in the database. This may have led to a potential selection bias.

In the computation of the sample size for our study, we used a prevalence of MetS obtained from a study among females in an urban population in Kenya. This may not accurately reflect the current study population characteristics. However, given that our population is female and in an urban setting, we think this limitation was minimised.

The sample size for the 15-17 years age group was relatively small. This may have influenced the confidence interval and performance metrics of waist circumference cut-off points in this age group. Future studies with larger and more representative samples of adolescents may provide more robust findings in this age group.

Defining MetS based on the presence of 2 or more components while excluding waist circumference, unlike using 3 or more components as in established guidelines can result in overestimation of the prevalence of MetS in the population.

Conclusions and recommendations

We found that a waist circumference of 80.3 cm was the mean optimal threshold for identifying metabolic syndrome in females between the ages of 18 and 49 years in Wakiso district, central Uganda. Furthermore, participants exhibited waist circumference cut-offs ranging from 78.9 cm to 91.1 cm based on age group. This study highlights the significance and precision of the existing waist circumference guidelines in healthcare. This study calls for the utilization of a waist circumference cut-off threshold to predict metabolic syndrome among reproductive-age females in Uganda, based on age group. The findings of this research may be relevant to healthcare providers, policymakers, public health practitioners and researchers. Further research in a wider range of populations encompassing both rural and urban settings is needed to validate these waist circumference cut-off points.

Acknowledgements

We appreciate all the study participants and research assistants who supported the data collection process. We thank all the local leaders and the district health management team members in Wakiso district who facilitated the research teams. We appreciate Dr Arthur Bagonza (PhD) and Dr Freddie Kitutu (PhD) for reviewing the manuscript.

References

1. Paley CA, Johnson MI. Abdominal obesity and metabolic syndrome: exercise as medicine? BMC Sports Science, Medicine and Rehabilitation. 2018;10:1-8.
2. Bovolini A, Garcia J, Andrade MA, Duarte JA. Metabolic syndrome pathophysiology and predisposing factors. International Journal of Sports Medicine. 2021;42(03):199-214.
3. Wang HH, Lee DK, Liu M, Portincasa P, Wang DQ-H. Novel insights into the pathogenesis and management of the metabolic syndrome. Pediatric gastroenterology, hepatology & nutrition. 2020;23(3):189.
4. Alberti KGMM, Zimmet P, Shaw J. Metabolic syndrome—a new world-wide definition. A consensus statement from the international diabetes federation. Diabetic medicine. 2006;23(5):469-80.
5. Alberti K, Eckel RH, Grundy SM, Zimmet PZ, Cleeman JI, Donato KA, et al. Harmonising the Metabolic Syndrome a Joint Interim Statement of the International Diabetes Federation Task Force on Epidemiology and Prevention; National Heart, Lung, and Blood Institute; American Heart Association; World Heart Federation; International Atherosclerosis Society; and International Association for the study of Obesity. Circulation. 2009;120(16):1640-5.
6. Alberti G, Zimmet P, Shaw J, Grundy SM. The IDF consensus worldwide definition of the metabolic syndrome. Brussels: International Diabetes Federation. 2006:1-23.
7. Okafor CI. The metabolic syndrome in Africa: Current trends. Indian journal of endocrinology and metabolism. 2012;16(1):56-66.
8. Bowo-Ngandji A, Kenmoe S, Ebogo-Belobo JT, Kenfack-Momo R, Takuissu GR, Kengne-Nde C, et al. Prevalence of the metabolic syndrome in African populations: A systematic review and meta-analysis. PloS one. 2023;18(7):e0289155.
9. Saklayen MG. The global epidemic of the metabolic syndrome. Current hypertension reports. 2018;20(2):1-8.
10. Noubiap JJ, Nansseu JR, Lontchi-Yimagou E, Nkeck JR, Nyaga UF, Ngouo AT, et al. Geographic distribution of metabolic syndrome and its components in the general adult population: A meta-analysis of global data from 28 million individuals. Diabetes research and clinical practice. 2022;188:109924.
11. Faijer-Westerink HJ, Kengne AP, Meeks KA, Agyemang C. Prevalence of metabolic syndrome in sub-Saharan Africa: A systematic review and meta-analysis. Nutrition, Metabolism and Cardiovascular Diseases. 2020;30(4):547-65.
12. Hwang Y-C, Jee J-H, Oh EY, Choi Y-H, Lee M-S, Kim K-W, et al. Metabolic syndrome as a predictor of cardiovascular diseases and type 2 diabetes in Koreans. International journal of cardiology. 2009;134(3):313-21.
13. Mohamed SM, Shalaby MA, El-Shiekh RA, El-Banna HA, Emam SR, Bakr AF. Metabolic syndrome: risk factors, diagnosis, pathogenesis, and management with natural approaches. Food Chemistry Advances. 2023;3:100335.
14. Handelsman Y, Butler J, Bakris GL, DeFronzo RA, Fonarow GC, Green JB, et al. Early intervention and intensive management of patients with diabetes, cardiorenal, and metabolic diseases. Journal of Diabetes and its Complications. 2023;37(2):108389.
15. Crowther NJ, Norris SA. The current waist circumference cut point used for the diagnosis of metabolic syndrome in sub-Saharan African women is not appropriate. PloS one. 2012;7(11):e48883.
16. Fang H, Berg E, Cheng X, Shen W. How to best assess abdominal obesity. Current opinion in clinical nutrition and metabolic care. 2018;21(5):360.
17. Ross R, Neeland IJ, Yamashita S, Shai I, Seidell J, Magni P, et al. Waist circumference as a vital sign in clinical practice: a Consensus Statement from the IAS and ICCR Working Group on Visceral Obesity. 2020. Report No.: 1759-5029 Contract No.: 3.

18. Misra A, Wasir JS, Vikram NK. Waist circumference criteria for the diagnosis of abdominal obesity are not applicable uniformly to all populations and ethnic groups. Nutrition. 2005;21(9):969-76.
19. Nicolaidis S. Environment and obesity. Metabolism. 2019;100:153942.
20. WHO. Waist circumference and waist-hip ratio: report of a WHO expert consultation, Geneva, 8-11 December 2008. Geneva; 2011.
21. Bener A, Yousafzai MT, Darwish S, Al-Hamaq AO, Nasralla EA, Abdul-Ghani M. Obesity index that better predict metabolic syndrome: body mass index, waist circumference, waist hip ratio, or waist height ratio. Journal of obesity. 2013;2013.
22. Gharipour M, Sarrafzadegan N, Sadeghi M, Andalib E, Talaie M, Shafie D, et al. Predictors of metabolic syndrome in the Iranian population: waist circumference, body mass index, or waist to hip ratio? Cholesterol. 2013;2013:198384.
23. Beydoun MA, Kuczmarski MTF, Wang Y, Mason MA, Evans MK, Zonderman AB. Receiver-operating characteristics of adiposity for metabolic syndrome: the Healthy Aging in Neighborhoods of Diversity across the Life Span (HANDLS) study. Public health nutrition. 2011;14(01):77-92.
24. Alberti G, Zimmet P, Shaw J, Grundy SM. The IDF consensus worldwide definition of the metabolic syndrome. Brussels: International Diabetes Federation. 2006;23(5):469-80.
25. Consultation WE. Waist circumference and waist-hip ratio. Report of a WHO Expert Consultation Geneva: World Health Organization. 2008;2008:8-11.
26. Grieger JA, Bianco-Miotto T, Grzeskowiak LE, Leemaqz SY, Poston L, McCowan LM, et al. Metabolic syndrome in pregnancy and risk for adverse pregnancy outcomes: A prospective cohort of nulliparous women. PLoS medicine. 2018;15(12):e1002710.
27. dos Prazeres Tavares H, Dos Santos DCDM, Abbade JF, Negrato CA, De Campos PA, Calderon IMP, et al. Prevalence of metabolic syndrome in non-diabetic, pregnant Angolan women according to four diagnostic criteria and its effects on adverse perinatal outcomes. Diabetology & Metabolic Syndrome. 2016;8:1-12.
28. Bo S, Menato G, Gallo M-L, Bardelli C, Lezo A, Signorile A, et al. Mild gestational hyperglycemia, the metabolic syndrome and adverse neonatal outcomes. Acta obstetricia et gynecologica Scandinavica. 2004;83(4):335-40.
29. Clayton GL, Borges MC, Lawlor DA. The impact of reproductive factors on the metabolic profile of females from menarche to menopause. Nature Communications. 2024;15(1):1103.
30. Mazza E, Troiano E, Ferro Y, Lisso F, Tosi M, Turco E, et al. Obesity, Dietary Patterns, and Hormonal Balance Modulation: Gender-Specific Impacts. Nutrients. 2024;16(11):1629.
31. Zheng L, Yang L, Guo Z, Yao N, Zhang S, Pu P. Obesity and its impact on female reproductive health: Unraveling the connections. Frontiers in Endocrinology. 2024;14:1326546.
32. Araújo J, Ramos E. Paediatric obesity and cardiovascular risk factors–A life course approach. Porto biomedical journal. 2017;2(4):102-10.
33. UBOS. Population and Censuses Kampala: UBOS; 2020 [Available from: https://www.ubos.org/explore-statistics/20/.
34. Sullivan K, Dean A. OPENEPI: A Web-based epidemiologic and statistical calculator for Public Health. 2009; 124: 471–474. 2009.
35. Kaduka LU, Kombe Y, Kenya E, Kuria E, Bore JK, Bukania ZN, et al. Prevalence of metabolic syndrome among an urban population in Kenya. Diabetes Care. 2012;35(4):887-93.
36. WHO. WHO STEPS surveillance manual: the WHO STEPwise approach to chronic disease risk factor surveillance. Geneva: World Health Organization; 2005. Report No.: 9241593830.
37. Petermann-Rocha F, Martínez-Sanguinetti MA, Ho FK, Celis-Morales C, Pizarro A. Optimal cut-off points for waist circumference in the definition of metabolic syndrome in Chile. Public Health Nutrition. 2020;23(16):2898-903.
38. Owolabi EO, Ter Goon D, Adeniyi OV, Ajayi AI. Optimal waist circumference cut-off points for predicting metabolic syndrome among low-income black South African adults. BMC research notes. 2018;11:1-5.

39. Nguyen KA, Peer N, de Villiers A, Mukasa B, Matsha TE, Mills EJ, et al. Optimal waist circumference threshold for diagnosing metabolic syndrome in African people living with HIV infection. Plos one. 2017;12(9):e0183029.
40. Lubogo D, Wamani H, Mayega RW, Mutungi G, Ssenyonga R, Orach CG. Metabolic syndrome and associated factors among females of reproductive age in Wakiso district, central Uganda: A cross sectional study. 2024.
41. StataCorp L. StataCorp stata statistical software: Release 14. StataCorp LP: College Station, TX, USA. 14.1 ed2015.
42. Fluss R, Faraggi D, Reiser B. Estimation of the Youden Index and its associated cutoff point. Biometrical Journal: Journal of Mathematical Methods in Biosciences. 2005;47(4):458-72.
43. Andreu A, Casals G, Vinagre I, Flores L. Obesity management in women of reproductive age. Endocrinología, Diabetes y Nutrición (English ed). 2023;70:85-94.
44. Grover S, Brandt J, Reddy U, Ananth C. Chronic hypertension, perinatal mortality and the impact of preterm delivery: a population-based study. BJOG: An International Journal of Obstetrics & Gynaecology. 2022;129(4):572-9.
45. Alberti, Kurt George Matthew Mayer, Zimmet Paul, Shaw. J. Metabolic Syndrome—a new world-wide definition. A consensus statement from the International Diabetes Federation. Diabetic medicine. 2006;23(5):469-80.
46. Magalhaes P, Capingana DP, Mill JG. Prevalence of the metabolic syndrome and determination of optimal cut-off values of waist circumference in university employees from Angola. South African Journal of Diabetes and Vascular Disease. 2015;12(1):35-40.
47. Cheong KC, Ghazali SM, Hock LK, Yusoff AF, Selvarajah S, Haniff J, et al. Optimal waist circumference cut-off values for predicting cardiovascular risk factors in a multi-ethnic Malaysian population. Obesity research & clinical practice. 2014;8(2):e154-e62.
48. Ogawa D, Kahara K, Shigematsu T, Fujii S, Hayakawa N, Okazaki M, et al. Optimal cut-off point of waist circumference for the diagnosis of metabolic syndrome in Japanese subjects. Journal of Diabetes Investigation. 2010;1(3):117-20.
49. Sinaga M, Worku M, Yemane T, Tegene E, Wakayo T, Girma T, et al. Optimal cut-off for obesity and markers of metabolic syndrome for Ethiopian adults. Nutrition journal. 2018;17(1):1-12.
50. Tladi D, Mokgatlhe L, Shaibu S, Nell T, Mitchell R, Mokgothu C, et al. Determination of optimal cut-off values for waist circumferences used for the diagnosis of the metabolic syndrome among Batswana adults (ELS 32). Cardiovascular Journal of Africa. 2020;31(6):314-8.
51. Motala AA, Esterhuizen T, Pirie FJ, Omar MA. The prevalence of metabolic syndrome and determination of the optimal waist circumference cutoff points in a rural South African community. Diabetes care. 2011;34(4):1032-7.
52. Casari S, Di Paola M, Banci E, Diallo S, Scarallo L, Renzo S, et al. Changing dietary habits: the impact of urbanization and rising socio-economic status in families from Burkina Faso in Sub-Saharan Africa. Nutrients. 2022;14(9):1782.
53. Subramoney S, BjöRkeluND C, Guo X, Skoog I, Bosaeus I, Lissner L. Age-related differences in recommended anthropometric cut-off point validity to identify cardiovascular risk factors in ostensibly healthy women. Scandinavian journal of public health. 2014;42(8):827-33.
54. St-Onge M-P, Gallagher D. Body composition changes with aging: the cause or the result of alterations in metabolic rate and macronutrient oxidation? Nutrition. 2010;26(2):152-5.
55. Kohn MA, Newman TB. The walking man approach to interpreting the receiver operating characteristic curve and area under the receiver operating characteristic curve. Journal of Clinical Epidemiology. 2023;162:182-6.
56. Shan G. Improved confidence intervals for the Youden index. PloS one. 2015;10(7):e0127272.

Supporting Information

S2 Fig: ROC curve for 18-49 years old females in Wakiso district, 2023

S3 Fig: ROC curve for 15-17 years old females in Wakiso district, 2023

S4 Fig: ROC curve for 18-24 years old females in Wakiso district, 2023

S5 Fig: ROC curve for 25-34 years old females in Wakiso district, 2023

S6 Fig: ROC curve for 35-44 years old females in Wakiso district, 2023

S7 Fig: ROC curve for 45-49 years old females in Wakiso district, 2023

20th December 2024

Dear Editor and Reviewers,

We thank the Editor and the Reviewers for taking the time to assess our manuscript. We appreciate the detailed and insightful comments. A point-by-point response to the reviewer's comments is below

PLOS Global Public Health Decision: Revision required [PGPH-D-24-00473R1] - [EMID:a9d1fcfcb0c82d27]

PLOS Global Public Health

CC: sangeetha.shyam@urv.cat, sangeeshyam@gmail.com
PGPH-D-24-00473R1
Optimal waist circumference cut-off points for predicting Metabolic Syndrome among females of reproductive age in Wakiso district, central Uganda
PLOS Global Public Health

Dear Dr. Lubogo,

Thank you for submitting your manuscript to PLOS Global Public Health. After careful consideration, we feel that it has merit but does not fully meet PLOS Global Public Health's publication criteria as it currently stands. Therefore, we invite you to submit a revised version of the manuscript that addresses the points raised during the review process.

Response:
Thank you for your consideration.

Please submit your revised manuscript by Jan 02 2025 11:59PM. If you will need more time than this to complete your revisions, please reply to this message or contact the journal office at globalpubhealth@plos.org. When you're ready to submit your revision, log on to https://www.editorialmanager.com/pgph/ and select the 'Submissions Needing Revision' folder to locate your manuscript file.

Please include the following items when submitting your revised manuscript:

- A rebuttal letter that responds to each point raised by the editor and reviewer(s). You should upload this letter as a separate file labeled 'Response to Reviewers'.
- A marked-up copy of your manuscript that highlights changes made to the original version. You should upload this as a separate file labeled 'Revised Manuscript with Track Changes'.
- An unmarked version of your revised paper without tracked changes. You should upload this as a separate file labeled 'Manuscript'.

Guidelines for resubmitting your figure files are available below the reviewer comments at the end of this letter.

We look forward to receiving your revised manuscript.
Kind regards,
Sangeetha Shyam, M.Sc., PhD
Academic Editor
PLOS Global Public Health
Journal Requirements:

Please review your reference list to ensure that it is complete and correct. If you have cited papers that have been retracted, please include the rationale for doing so in the manuscript text, or remove these references and replace them with relevant current references. Any changes to the reference list should be mentioned in the rebuttal letter that accompanies your revised manuscript. If you need to cite a retracted article, indicate the article's retracted status in the References list and also include a citation and full reference for the retraction notice.

Response:

We reviewed and verified the reference list to ensure its completeness and correctness. We added one reference to the reference list. The reference addresses the reviewer's comment regarding the description of physical activity levels and alcohol use levels (low, intermediate, and high) and how the measurement standards were defined. Please see lines 176 to 177

Additional Editor Comments (if provided):

The authors are commended for addressing the reviewer comments in this version of the manuscript. They are encouraged to address the minor comments from the reviewer

[Note: HTML markup is below. Please do not edit.]

Reviewers' comments:

Reviewer's Responses to Questions

Comments to the Author
1. If the authors have adequately addressed your comments raised in a previous round of review and you feel that this manuscript is now acceptable for publication, you may indicate that here to bypass the "Comments to the Author" section, enter your conflict of interest statement in the "Confidential to Editor" section, and submit your "Accept" recommendation.

Reviewer #2: All comments have been addressed

2. Does this manuscript meet PLOS Global Public Health's publication criteria? Is the manuscript technically sound, and do the data support the conclusions? The manuscript must describe methodologically and ethically rigorous research with conclusions that are appropriately drawn based on the data presented.

Reviewer #2: Yes

3. Has the statistical analysis been performed appropriately and rigorously?

Reviewer #2: Yes

4. Have the authors made all data underlying the findings in their manuscript fully available (please refer to the Data Availability Statement at the start of the manuscript PDF file)?

The PLOS Data policy requires authors to make all data underlying the findings described in their manuscript fully available without restriction, with rare exception. The data should be provided as part of the manuscript or its supporting information, or deposited to a public repository. For example, in addition to summary statistics, the data points behind means, medians and variance measures should be available. If there are restrictions on publicly sharing data—e.g. participant privacy or use of data from a third party—those must be specified.

Reviewer #2: Yes

5. Is the manuscript presented in an intelligible fashion and written in standard English?

PLOS Global Public Health does not copyedit accepted manuscripts, so the language in submitted articles must be clear, correct, and unambiguous. Any typographical or grammatical errors should be corrected at revision, so please note any specific errors here.

Reviewer #2: Yes

6. Review Comments to the Author

Please use the space provided to explain your answers to the questions above. You may also include additional comments for the author, including concerns about dual publication, research ethics, or publication ethics. (Please upload your review as an attachment if it exceeds 20,000 characters)

Reviewer #2: Dear Author
Thank you for the opportunity to review your manuscript on MetS prevalence and WC as a predictor. I commend your work and have a few suggestions for improving clarity.

Response:

Thank you so much for the positive comments.

- BMI Classification: Use standard categories like WHO's cut-offs. However, since adolescents are analyzed in Table 1, the BMI categorization should be done separately for adolescents based on age- and sex-specific percentiles (WHO guidelines) or remove adolescent throughout analysis [because I also saw in table 2 results that reveal that for the 15-17 age group, the AUC value of 0.81 indicates good performance, but the broad confidence interval (0.690-0.937) may be due to the small sample size (n=63). Sensitivity (87%) is high, but specificity (75%) is moderate, and the Youden index (0.620) is relatively low. larger sample sizes in future studies could improve the results.

Response:

Thank you for your valuable comments. We re-analysed the data for adults (18-49 years) after excluding participants aged 15 to 17 years. We used the standard BMI cut-off points as per the WHO recommendations. Please see Table 1 on page 12.

For adolescents (15 to 17 years), as per your recommendation, we used the BMI for age percentiles for girls aged 5-19 years as recommended by WHO. Please see page 12 and 13 for the updated analysis.

We have acknowledged the limitation of the small sample size for the 15-17 years age group and its potential effect on the confidence intervals and performance metrics. We have also suggested that future studies with larger sample sizes among adolescents could enhance the robustness of the findings and improve other performance metrics. Please see lines 406 to 409 on page 22.

- Physical Activity Levels: Specify the classification method, e.g., WHO guidelines, PAL, or GPAQ.

- Alcohol Use Levels: Define low, intermediate, and high levels, and mention the measurement standard used.

Response:

Thank you for the comment. The physical activity levels and alcohol use levels (low, intermediate, and high) and the measurement standards were defined in detail in a previous manuscript currently under peer review. To avoid repetition, we have referred the reader to that manuscript for further information. Please see lines 176 to 177. Thank you.

These comments are also highlighted in the attached draft.

Response:

We have reviewed the comments highlighted in the draft shared and addressed all of them. Thank you

Final Remarks
With these updates, the paper will be clearer and more informative. Thank you for considering these suggestions. I look forward to the impact of your research.

Best regards,

Response:

Thank you so much for the insightful comments. We hope that we have addressed all the comments to the satisfaction of the reviewer and the Editor.

---

7. PLOS authors have the option to publish the peer review history of their article (what does this mean?). If published, this will include your full peer review and any attached files.

Do you want your identity to be public for this peer review? If you choose “no”, your identity will remain anonymous but your review may still be made public.

For information about this choice, including consent withdrawal, please see our Privacy Policy.

Reviewer #2: **Yes:** Lakshmi priya Nagarajan

[NOTE: If reviewer comments were submitted as an attachment file, they will be attached to this email and accessible via the submission site. Please log into your account, locate the manuscript record, and check for the action link "View Attachments". If this link does not appear, there are no attachment files.]

While revising your submission, please upload your figure files to the Preflight Analysis and Conversion Engine (PACE) digital diagnostic tool, https://pacev2.apexcovantage.com/. PACE helps ensure that figures meet PLOS requirements. To use PACE, you must first register as a user. Registration is free. Then, login and navigate to the UPLOAD tab, where you will find detailed instructions on how to use the tool. If you encounter any issues or have any questions when using PACE, please email PLOS at figures@plos.org. Please note that Supporting Information files do not need this step.

***Response**: This was done. Thank you.*

In compliance with data protection regulations, you may request that we remove your personal registration details at any time. (Remove my information/details). Please contact the publication office if you have any questions.
