# Supplementary material for: Optimal waist circumference cut-off points for predicting metabolic syndrome among females of reproductive age in Wakiso district, central Uganda
Source: PLOS Glob Public Health. 2025 Apr 9;5(4):e0003059. doi: 10.1371/journal.pgph.0003059 (PMC11981207; doi:10.1371/journal.pgph.0003059)
Supplement: Attachment — Submitted filename: Response_to_Reviewers_auresp_3.pdf [file pgph.0003059.s014.pdf]

RESPONSE TO REVIEWERS

PGPH-D-24-00473R2
Optimal waist circumference cut-off points for predicting metabolic syndrome among females of reproductive age in Wakiso district, central Uganda
PLOS Global Public Health

Reviewer #2: Dear Author
We believe the manuscript can be further strengthened by enhancing its scientific clarity and depth of analysis. Specifically: Strengthening the Analysis: Incorporating detailed association analyses while accounting for potential confounding factors, particularly by age stratification, would provide stronger support for the conclusions.

Response:

Thank you for your comment. Considering that the primary objective of our study was to determine optimal waist circumference cut-off points for predicting metabolic syndrome among women of reproductive age, we have conducted a detailed analysis of the data including age stratification, as presented in Table 1 and Table 2 on pages 12,13 and 15, 16 respectively. we believe this sheds light on age-related trends and associations. Thank you.

Broader Anthropometric Insights: Including waist-to-height ratio (WHtR) as an additional measure and discussing its relevance in the context of regional variations in mean height within African populations will enhance the interpretation of central obesity and related risks in the discussion part.

Response:

We have added a section including the waist-to-height ratio and discussed its relevance in the context of regional variation in mean height within African populations. We also suggested a recommendation for future research on the waist-to-height ratio. Please see pages 23 and 24 pages 396 to 418.

Clarifying Context: Providing a thoughtful discussion of the study's scope and constraints, framed with supporting evidence, will help improve the manuscript's scientific impact.

Response:

We have considered the study's scope and constraints in our work extensively as reflected in the study limitations on page 24 (lines 419 to 436) and page 22 line 389 to 391, which we have updated for clarity. Thank you for the comments.

We appreciate your valuable contribution to this field of research and encourage you to consider these suggestions to further enhance your manuscript.

Response:

Thank you for the suggestions and comments. We have considered them and we believe they have enhanced the quality and clarity of our manuscript.

I have attached my detailed comments directly within the manuscript for your reference.

Detailed comments within the manuscript:

I noticed that the results section is missing important details such as the distribution of BMI percentiles for young adults (15-17 yrs) education level, physical activity, and salt intake. These key aspects are mentioned but not clearly presented in the results. Could you please provide a more comprehensive presentation of these factors with the appropriate distribution and clarity? This would improve the completeness and readability of the results

Response:

Thank you for the comments. We have added a detailed and comprehensive presentation of the key characteristics/variables with their appropriate distribution from the Table of Characteristics (Table 1). Please see pages 12-13 lines 234 to 237. Thank you.

Question to the Author:

In your analysis, the age group (15–49 years) is presented with significant variation in sample size distribution across different age ranges (e.g., 15–17 years with 63 participants versus 25–34 years with 252

participants). Given the disparity in age group sizes, could this imbalance have affected the generalizability or representativeness of the findings? Additionally, was any weighting or stratification method applied to account for this variation? If not, it would be beneficial to address this limitation in the discussion section, as it could influence the overall conclusions.
Best wishes for your continued research endeavours.

Response:

Thank you for your comment. Sampling weights were applied to correct selection probabilities at three levels including enumeration area, household, and individual levels and response rates to ensure that the final sample (697) was representative of the target population (15-49 years). We indicated this on page 7, lines 120 to 126.

Since our objective was to determine the average waist circumference and age-specific cut-off points for predicting MetS, we believe these weights could have accounted for differences in age distribution.

While we acknowledge that certain age groups had smaller sample sizes, we believe the applied weights mitigated potential biases in estimating age-specific cut-offs. We have addressed this in the discussion section and acknowledged that variations in sample sizes across age groups might have affected the precision of some estimates. Please see page 24 lines 429 to 433. Thank you.

The inclusion of education levels in this paper may not be relevant, as no associations with abdominal obesity or other risk factors are being analyzed. Additionally, the mix of school-going individuals and early adults makes the classification unclear. Would it be more appropriate to remove this information to improve the clarity and focus of the manuscript?

Response:

We agree with the reviewer's suggestion. We have removed information on education levels, as well as the school-going individuals (15-17 years) to improve the clarity and focus of the manuscript. Please see Table 1 on page 12. Thank you.

There is no need to mention the 0% category for BMI-for-age percentiles and BMI ranges. It would improve the clarity and conciseness of the data presentation if these values are omitted.

Response:

We agree with the reviewer. We have removed the 0% category for BMI-for-age percentiles and BMI ranges. Please see Table 1 page 12. Thank you.

Your descriptive table can be more informative and supportive of the main analysis by aligning it with your hypothesis, which focuses on stratification. Consider presenting demographic information classified by age categories. This will help identify potential confounding factors, making it easier to adjust in association analyses. Additionally, the absence of association analysis is a significant limitation. Including this analysis would strengthen the manuscript's overall meaning and analytical depth.

Response:

We agree with the reviewer. We have presented the demographic information classified by age categories. Due to the scope of the paper, we restricted ourselves from doing association analysis to keep in line with the primary objectives of the paper. Please see Table 1, page 12. Thank you.

The results show that HDL cholesterol, triglycerides, and fasting blood sugar (FBS) did not show any significant differences across the age groups (HDL: p=0.958, TG:p=0.157), , and FBS ,p=0.572) not mentioned). Could you please clarify or update the results section to accurately reflect that these components did not show significant variation across age groups, as indicated by the statistical values? This would ensure the consistency and clarity of the reported findings.

Response:

Thank you. We have updated the results section and included the suggested information. Please see page 14, lines 252-253. Thank you.

Please include a discussion of the regional variation in mean height across different African regions (East, North, South, and Central). Highlight how these variations may influence waist circumference (WC) cut-offs used for assessing central obesity. Given that shorter populations tend to exhibit higher waist-to-height ratios (WHtR) for the same WC, failing to adjust for height differences could result in misclassification of obesity risk. Consider

citing studies or data demonstrating these height differences and their impact on abdominal fat distribution and metabolic risk.

Response:

Thank you for this suggestion. We have considered the suggestion and included a section in the discussion in line with the study's primary objectives and discussed the variation of height and how it may influence waist circumference, and subsequently, the need to consider waist to-height ratio. We have also cited studies demonstrating these height differences. We related this to our study and recommended research to divulge into this concept in future. Thank you. Please see pages 23 and 24, lines 396 to 418.

Incorporation of WHtR in Tables and Analysis:

Tables 1 and 2: Add WHtR as a variable, showing the proportion of participants with WHtR <0.5 and ≥ 0.5. This provides a better representation of obesity-related health risks adjusted for height.

<u>Response:</u>

We have added WHtR as a variable in Tables 1 and 2 as suggested. Thank you.

Thank you for the detailed comments. We have read and attended to the comments as outlined in the manuscript. We hope that this revised version meets your and the editor's expectations. Thank you so much.

Dr David Lubogo

Corresponding author
