# Supplementary material for: Optimal waist circumference cut-off points for predicting metabolic syndrome among females of reproductive age in Wakiso district, central Uganda
Source: PLOS Glob Public Health. 2025 Apr 9;5(4):e0003059. doi: 10.1371/journal.pgph.0003059 (PMC11981207; doi:10.1371/journal.pgph.0003059)
Supplement: Attachment — Submitted filename: Reviewer comments 24.8.24.docx [file pgph.0003059.s008.docx]

**Abstract Corrections**

**Page 2, Lines 31-32**:

**The data collected were from a cross-sectional study conducted in Wakiso district, central Uganda, involving 697 randomly selected females aged 15 to 49, between June 9, 2021, and August 17, 2021.**

**Q1: Age Range Impact on Metabolic Syndrome**

The study includes females aged 15 to 49, covering the reproductive age range. As metabolic risk increases with age, older participants are at higher risk. This broad age range could potentially dilute the overall prevalence of metabolic syndrome (MS) or alter the waist circumference (WC) cut-offs, as the younger age group typically has a lower risk compared to adults. Visceral fat distribution is significantly higher in adults than in teenagers or younger women, which may influence WC measurements and their association with MS. Additionally, hormonal changes and variations in body composition during different life stages, such as puberty and menopause, can affect fat distribution patterns, leading to varying MS risk profiles across age groups.

**Q2: Optimal WC Cut-offs**

**Page 2, Lines 39-41**:

**Variations in optimal WC thresholds were observed across different age groups: 79.4 cm for 15-24 years, 79.9 cm for 25-34 years, 85.6 cm for 35-44 years, and 91.1 cm for 45-54 years.**

**Q2a**: It seems there may be a discrepancy; the correct value appears to be 79.4 cm rather than 97.4 cm.

**Q2b**: Adulthood is generally defined as starting at 18 years of age. International standards set by the IDF, NCEP, and WHO provide waist circumference cut-offs primarily applicable to adults aged 18 years and older and do not include cut-offs for teenagers or adolescents. In this analysis, adolescents and adults aged 15-24 years were grouped together, which may affect the results for this group. Analyzing the 15-17 years age group separately could provide more accurate and relevant assessments.

**Q3: Criteria for Metabolic Syndrome**

**Page 2, Lines 33-34**:
**MetS status was identified based on two or more MetS components excluding WC.**

**Q3a**: Could you clarify the criteria used to exclude WC from MetS status? If you referred to any internal criteria, please provide the reference.

**Q3b**: According to Alberti et al. (2009), Metabolic Syndrome (MetS) should be classified based on the presence of any three abnormalities, including ethnic-specific waist circumference criteria. However, in your study, only two abnormalities were considered, and WC was excluded. This might lead to potential misclassification if an individual with a normal WC but two other abnormalities is classified as having MetS. Did you assess this potential misclassification by comparing your criteria with international standards?

"I recommend using the approach by Cook et al. (2003) and Xi et al. (2020), which defines central obesity in adolescents by the 90th percentile of waist circumference (WC). Analyzing adolescent data separately with this method could improve the accuracy of Metabolic Syndrome (MetS) risk assessment and provide valuable age-specific insights, enhancing the precision of your study's findings

**Q4: Enhancing the Introduction**

**Revised Text**:
To enhance the depth and relevance of the introduction, consider including the following points:

1. "Obesity, with its associated metabolic and endocrine issues, can have a direct impact on women's reproductive health."
2. "Childhood obesity is a significant risk factor for developing metabolic diseases such as cardiovascular disease (CVD) and diabetes in later life."
3. "These conditions can exacerbate reproductive health issues, including polycystic ovary syndrome (PCOS), menstrual disturbances, and infertility."

**Q5: Inconsistency in MetS Criteria**

**Page 8, Lines 141-142**:
**Metabolic Syndrome was assessed by determining if an individual had three or more of the five components.**

**Page 2, Lines 33-34**:
**MetS status was identified based on two or more MetS components excluding WC.**

**Q5**: The definitions of MetS in these two sentences differ, indicating an inconsistency. Please review and, if necessary, include a table highlighting the differences.

**Figures and Tables**

**Page 10, Line 181**:

**Q6.** Is this figure S6 or S1?

**Page 10, Lines 185-186: Table 1 indicates that almost half of the participants (49.9%) exhibited two or more risk factors linked to MetS (≥ 2 MetS factors).**

**Q7.** Does this approach include Waist Circumference (WC) in the diagnosis, or was WC excluded?

**Table 2**: There is a typographical error in the table for the age category 15-45.

**Q8.** Should this be 15-54?

**Q9**. Provide the number (n) for each category in the columns.

**Q10**. Include the JIS and IDF criteria along with your current WC cut-off points (if any differences). Additionally, provide the prevalence percentage of each metabolic component overall and within each age category.

**Optimal WC Cut-offs and Regional Variations in various African studies.**

| Country [references] | n | Mean WC | Mean age of the study population | Cut-off point for women (cm |
| --- | --- | --- | --- | --- |
| Uganda [[24](https://bmcresnotes.biomedcentral.com/articles/10.1186/s13104-018-3136-9#ref-CR24)] | 6136 | 77.5(MALE & FEMALE) | >=18 year | ≥ 82 to ≥ 85 |
| South Africa [[19](https://bmcresnotes.biomedcentral.com/articles/10.1186/s13104-018-3136-9#ref-CR19)] | 123 | 93.48±15.68 | 25–65 years | 98 |
| Benin [[23](https://bmcresnotes.biomedcentral.com/articles/10.1186/s13104-018-3136-9#ref-CR23)] | 452 | 88.6±13.6 | 25 to 60 | 94 |
| **Angola [**[**31**](https://bmcresnotes.biomedcentral.com/articles/10.1186/s13104-018-3136-9#ref-CR31)**]** | **615** | **83.9 ± 13.5** | **>20** | **80.5** |
| South Africa [[18](https://bmcresnotes.biomedcentral.com/articles/10.1186/s13104-018-3136-9#ref-CR18)] | 947 | 86.0 ± 13.4 | >15 years | 92 |
| Benin [[37](https://bmcresnotes.biomedcentral.com/articles/10.1186/s13104-018-3136-9#ref-CR37)] | 541 | 87.6 ± 13.0 | 25- 60 | 90 |
| South Africa [[21](https://bmcresnotes.biomedcentral.com/articles/10.1186/s13104-018-3136-9#ref-CR21)] | 152 | - | 24–65 | 94 |
| South Africa [[38](https://bmcresnotes.biomedcentral.com/articles/10.1186/s13104-018-3136-9#ref-CR38)] | 1099 | - | 55–64 y | 94 |
| South Africa [[22](https://bmcresnotes.biomedcentral.com/articles/10.1186/s13104-018-3136-9#ref-CR22)] | 920 | - | 25–74-y | 94.6 |
| South Africa (present study) | 998 | - | ≥ 18 | ≥ 89.45 |

**Page 14, Lines 222-226**:

**Our study has identified an optimal WC threshold value that closely approximates the recommended cut-off of 80.0 cm as specified by the International Diabetes Federation for diagnosing Metabolic Syndrome (MetS) (27). An optimal WC of 80.5 cm was found among female university employees in Angola (28).**

**Q11a**: Except for the Angola study, optimal waist circumference (WC) cut-offs derived from various African regions suggest that the currently used 80 cm cut-off for women may overestimate abdominal obesity. Additionally, many of the studies included adults as volunteers, which may affect the results. It is advisable to recheck the analysis by either removing adolescents or providing a separate analysis for this group or do the age specific cutoff (use 90^th^ percentile).

**Q11b**It is my guess that the mean WC of your study population (86.4±13.8), including adolescents. The cut-off may be higher than the present 80.5 cm due to potential misclassification caused by the inclusion of adolescents. Please consider using age-specific categories and redoing the analysis. Kindly check.

**Page 14, Lines 226-228**:

**Elsewhere, some studies among a multiethnic Malaysian population (29) and in a Japanese population (30) found that 80.0 cm was an optimal WC for women.**

**Q12.**However, these studies involved ethnically different populations compared to Africans. Comparing your findings with those of your ethnic population and justifying the differences by considering factors such as age, socioeconomic status, lifestyle, dietary patterns, physical activity, and urbanization could provide a more accurate assessment. In the future, conducting a meta-analysis could help derive ethnicity- and age-specific WC cut-offs for Africans.

**Page 14, Lines 226-228**:

**The observed variation might be attributed to differences in factors such as genetics and body composition across different geographical regions. For instance, countries with higher prevalences of obesity, such as South Africa, often have higher optimal WC cut-off points (15, 33).**

**Q13**. The variation in waist circumference (WC) cut-off points for defining central obesity across regions is influenced by genetics, body composition, and socio-environmental factors. For instance, countries with higher obesity rates, like South Africa, often have higher WC cut-offs due to greater body fat and different fat distribution. Economic conditions and urbanization also affect lifestyle and dietary patterns, impacting obesity rates and WC measurements. Including these factors in your discussion can enhance the understanding and interpretation of WC variations in your results.

**In Pages 15-18, Lines 249-250**:

**Probable reasons for the variation could be due to changes in body composition with age, resulting in reduced muscle mass and increased body fat, and a reduction in metabolic rate with age, leading to weight gain if dietary intake and physical activity remain the same. Do you have data on physical activity and diet? If so, please include this information in your descriptive table. Add relevant references for these sentences.**

**In Pages 15-18, Lines 254-324**:

**Q14**. The discussion part repeats the results section, and the discussion points are limited. If your secondary objective is to focus on age and women health, then more information should be included in your manuscript. This will strengthen the discussion.
